# Neutrophils trigger a NF-κB dependent polarization of tumor-supportive stromal cells in germinal center B-cell lymphomas

**DOI:** 10.18632/oncotarget.4106

**Published:** 2015-05-12

**Authors:** Murielle Grégoire, Fabien Guilloton, Céline Pangault, Frédéric Mourcin, Phaktra Sok, Maelle Latour, Patricia Amé-Thomas, Erwan Flecher, Thierry Fest, Karin Tarte

**Affiliations:** ^1^ INSERM, UMR U917, Equipe Labellisée Ligue Contre le Cancer, Rennes, France; ^2^ Université Rennes 1, UMR917, Rennes, France; ^3^ EFS Bretagne, Rennes, France; ^4^ CHU de Rennes, Pôle Biologie, Rennes, France; ^5^ CHU de Rennes, Service de Médecine de L'enfant et de L'adolescent, Rennes, France; ^6^ CHU de Rennes, Service de Chirurgie Thoracique et Cardiovasculaire, Rennes, France

**Keywords:** B-cell lymphoma, tumor microenvironment, cell interaction, cell differentiation, lymph node

## Abstract

Both tumor-associated neutrophils (TAN) and cancer-associated fibroblasts (CAFs) display specific phenotypic and functional features and contribute to tumor cell niche. However, their bidirectional crosstalk has been poorly studied, in particular in the context of hematological malignancies. Follicular lymphomas (FL) and diffuse large B-cell lymphomas (DLBCL) are two germinal center-derived lymphomas where various cell components of infiltrating microenvironment, including TAN and CAFs, have been demonstrated to favor directly and indirectly malignant B-cell survival, growth, and drug resistance. We show here that, besides a direct and contact-dependent supportive effect of neutrophils on DLBCL B-cell survival, mediated through the BAFF/APRIL pathway, neutrophils and stromal cells cooperate to sustain FL B-cell growth. This cooperation relies on an overexpression of IL-8 by lymphoma-infiltrating stromal cells that could thereafter efficiently promote neutrophil survival and prime them to neutrophil extracellular trap. Conversely, neutrophils are able to activate stromal cells in a NF-κB-dependent manner, inducing their commitment towards an inflammatory lymphoid stroma phenotype associated with an increased capacity to trigger malignant B-cell survival, and to recruit additional monocytes and neutrophils through the release of CCL2 and IL-8, respectively. Altogether, a better understanding of the lymphoma-supporting effects of neutrophils could be helpful to design new anti-tumor therapeutic strategies.

## INTRODUCTION

Tumorigenesis is widely recognized as a non-cell autonomous process depending on the continuous active crosstalk between malignant cells and various stromal and immune cell subsets of their surrounding microenvironment. Tumor infiltrating neutrophils (TAN) have initially received less attention than their macrophage counterpart (TAM) until the demonstration that they could persist within inflamed tissues where they exhibit both phenotypic and functional heterogeneity, including the production of a wide range of cytokines and chemokines [1, 2]. TAN have been recently shown to exert both pro-and antitumor activities, including triggering of genomic instability, angiogenesis, immunosuppression, and tumor cell metastasis on the one hand *versus* direct cytotoxic effect and recruitment or activation of other effectors of innate and adaptive antitumor immunity on the other hand [3]. In turn, TAN recruitment and polarization are triggered by tumor cell-derived signals [4, 5]. Cancer-associated fibroblasts (CAFs) also contribute to tumor-supportive cell niche and have been shown to display tumor-specific transcriptomic, phenotypic, and functional features compared to normal tissue fibroblasts [6]. CAFs could support directly tumor cell survival, growth, metastasis, and drug resistance but they have also been involved in reshaping tumor microenvironment. Resident mesenchymal stromal cells (MSCs) are believed to be the major precursors of CAFs *in situ* and to acquire their tumor promoting properties after exposition to tumor-derived activating stimuli. Whereas their impact on neutrophil activation remains controversial, bone marrow (BM)-MSCs have been repeatedly shown to sustain neutrophil survival, in particular following activation by inflammatory stimuli and TLR ligands [7, 8]. IL-6 was proposed as the underlying molecular effector for this stroma-dependent anti-apoptotic activity. Recently, gastric cancer-derived MSCs have been specifically shown to promote neutrophil chemotaxis, survival, and activation through an IL-6/STAT-3 pathway [9]. However, few data are available concerning reciprocal interactions between TAN and CAFs in solid and hematological malignancies.

Follicular lymphoma (FL) and diffuse large B-cell lymphomas (DLBCL) result from the malignant transformation of germinal center (GC) B cells and are the two most frequent B-cell non-Hodgkin's lymphomas [10]. Both FL and DLBCL are generally disseminated diseases with frequent involvement of the BM that represents an ectopic supportive cell niche where CAFs display a specific gene expression profile (GEP) [11]. Transcriptomic signatures reflecting specific features of tumor microenvironment were shown to predict patient survival in FL and DLBCL [12, 13]. Phenotypic and functional alterations of infiltrating T cells and TAM have been described in both diseases [14, 15]. Furthermore, stromal cells prevent lymphoma B-cell apoptosis *in vitro* [16, 17] through various contact-dependent and independent mechanisms including the production of B cell-activating factor (BAFF) [18]. BAFF and a proliferation-inducing ligand (APRIL) are closely related ligands of the TNF superfamily and have been shown to trigger lymphoma B-cell survival through their receptors BAFFR, TACI, and BCMA [19, 20]. Activated neutrophils are well known producers of soluble BAFF [21] and are supposed to trigger the survival and differentiation of normal B cells into immunoglobulin-producing plasma cells [22]. In addition, a novel subset of BAFF- and APRIL-producing neutrophils able to stimulate immunoglobulin class switching, somatic hypermutation, and production by marginal zone B cells has been recently described in spleen [23] even if these results are disputable [24]. Whereas neutrophils are largely excluded from lymph nodes (LN) in steady state conditions, they could enter inflamed lymphoid organs and modulate adaptive immune response [25, 26]. Interestingly, DLBCL TAN overexpress APRIL *in situ* allowing its accumulation on tumor B cells *via* proteoglycan binding [27]. Accordingly, the neutrophil to lymphocyte ratio in blood is an independent prognostic factor in patients with DLBCL [28]. However, despite these promising results, functional interactions between neutrophils and malignant B cells remain to be explored.

The potential role of TAN and stromal cells in B-cell lymphomagenesis and the emerging field of stroma-neutrophil interaction led us to investigate whether these two cell subsets establish a bidirectional crosstalk in the context of B-cell lymphomas. We have previously demonstrated that MSCs obtained from FL-infiltrated BM (FL-MSCs) produce higher level of CCL2 compared to BM-MSCs from healthy donors (HD-MSCs) in association with increased monocyte recruitment and polarization into TAM-like cells [11]. We explored here the interplay between malignant B cells, stroma, and neutrophils. We demonstrated that FL-MSCs overexpressed IL-8 and triggered peripheral blood neutrophil recruitment. Moreover neutrophils and stromal cells cooperated to support malignant B-cell growth, owing to both a protection of neutrophils from spontaneous apoptosis, and an activation of stromal cells that acquired an inflammatory lymphoid stroma phenotype in contact with neutrophils. Finally, this crosstalk was associated with an activation of NF-κB pathway in stromal cells that sensitize neutrophils to neutrophil extracellular trap (NET) formation.

## RESULTS

### Neutrophils directly sustain malignant B-cell growth

**Figure 1:**
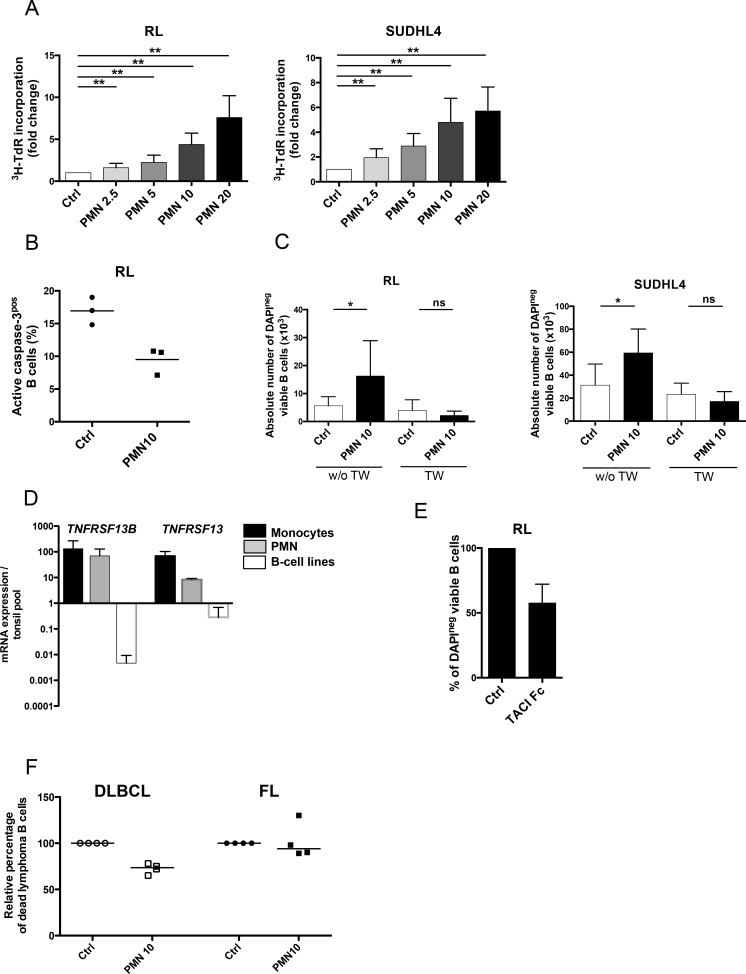
Neutrophils directly sustain the growth of malignant B-cell lines (**A**) GC-derived B cell lines were cultured in low serum concentration alone (Ctrl) or in the presence of different ratios of neutrophils (PMN)/B cells (2.5/1, 5/1, 10/1 or 20/1). Cell growth was evaluated by tritiated thymidine incorporation (^3^HTdR) at day 2 for RL (left) and day 3 for SUDHL4 (right). B cell alone proliferation was arbitrary assigned to 1. Results represent the mean ± SD from 10 experiments. ** *P* < 0.01. (**B**) Apoptosis was evaluated at day 1 on RL co-cultured or not with neutrophils as the percentage of active caspase-3^pos^ cells gated on CD19/CD20^pos^CD15^neg^ B cells. (**C**) GC-derived B cell lines were separated or not from neutrophils by a transwell (TW) insert, and B-cell growth was evaluated by determining the absolute number of DAPI^neg^CD19/CD20^pos^CD66b^neg^ viable B cells using FlowCount beads at day 3 for RL (left) and day 4 for SUDHL4 (right). Results represent the mean ± SD from 6 experiments. * *P* < 0.05. (**D**) *TNFRSF13B* and *TNFRSF13* gene expression were compared in purified CD14^pos^ monocytes (n=3), purified CD15^pos^ PMN (n=3), and RL, SUDHL4 and DOHH2 B-cell lines. The arbitrary value of 1 was assigned to a pool of five whole tonsil cells. Bars: mean+/−SD. (**E**) GC-derived B cell line were cultured in low serum concentration in the presence of neutrophils with or without (Ctrl) TACI-Fc. B-cell survival was evaluated by determining the percentage of DAPI^neg^CD19/CD20^pos^CD66b^neg^ viable B cells at day 3, and compared to that of B cells cultured with PN without TACI-Fc, arbitrary assigned to 100%. Results represent the mean ± SD from 3 experiments. (**F**) Purified B cells sorted from 4 DLBCL and 4 FL LN were cultured in the presence or not of neutrophils. B-cell death was evaluated by determining the percentage of TOPRO-3^pos^CD19/CD20^pos^CD66b^neg^ B cells at day 1 for DLBCL and day 3 for FL and compared to that of B cells cultured alone, arbitrary assigned to 100%. Bars: median.

We first decided to test whether neutrophils directly modified the growth of GC-derived lymphoma B-cell lines. Purified resting neutrophils did not display any cytotoxic effect towards malignant B cells but were conversely able to reverse serum deprivation-induced growth arrest of RL and SUDHL4 in a dose-dependent manner (Figure 1A). Similar results were obtained with DOHH2 (data not shown). This supportive effect was associated with a decreased B-cell apoptosis (Figure 1B). Interestingly, separation of B cells and neutrophils by a 0.4-μm transwell filter completely abrogated lymphoma-promoting activity of neutrophils, indicating a key role for direct cell contact (Figure 1C). In agreement, whereas RL and SUDHL4 expressed BAFFR, TACI, BCMA and the APRIL co-receptor syndecan-4, addition of soluble BAFF or APRIL did not increase malignant B-cell growth (data not shown). However, circulating neutrophils, unlike malignant B cells, constitutively express significant amounts of *TNFRSF13B/BAFF* and *TNFRSF13/APRIL* (Figure 1D) and, in our co-culture experiments, simultaneous blockade of BAFF and APRIL activity by TACI-Fc significantly reduced the malignant B-cell supportive effect of neutrophils suggesting a role for membrane-bound molecules (Figure 1E). Finally, we explored the direct effect of neutrophils on primary malignant B cells obtained from FL and DLBCL including the two major molecular subtypes have been defined in this disease, the GC B cell-like (GCB) and the non-GCB DLBCL that differ by their response to chemotherapy and cure rate [29]. Interestingly, we were able to confirm the survival effect of neutrophils on B cells purified from 2 GCB and 2 non-GCB DLBCL samples, but not on 4 FL samples (Figure 1F).

To further investigate this discrepancy, and since APRIL-expressing TAN have been described as intermingled with DLBCL B cells [27], we decided to check by immunofluorescence the presence of neutrophils in invaded FL LN compared to chronically inflamed tonsils. The number of CD15^pos^ cells is high and rather similar in inflamed and malignant samples. Most importantly, neutrophils remained essentially restricted to interfollicular and perifollicular zones, suggesting limited direct contact with malignant B cells but potential interactions with transglutaminase-expressing fibroblastic reticular cells (FRC), a lymphoid stromal cell subset recently involved in the survival of malignant FL B cells and in the homeostasis of normal B cells [11, 17, 30] (Supplemental Figure S1). Collectively, these data demonstrated that neutrophils could directly support *in vitro* DLBCL malignant B-cell growth and raised the question of their indirect effect through the crosstalk with lymphoid stroma in FL.

### Infiltrating stromal cells recruit neutrophils and protect them from apoptosis

**Figure 2:**
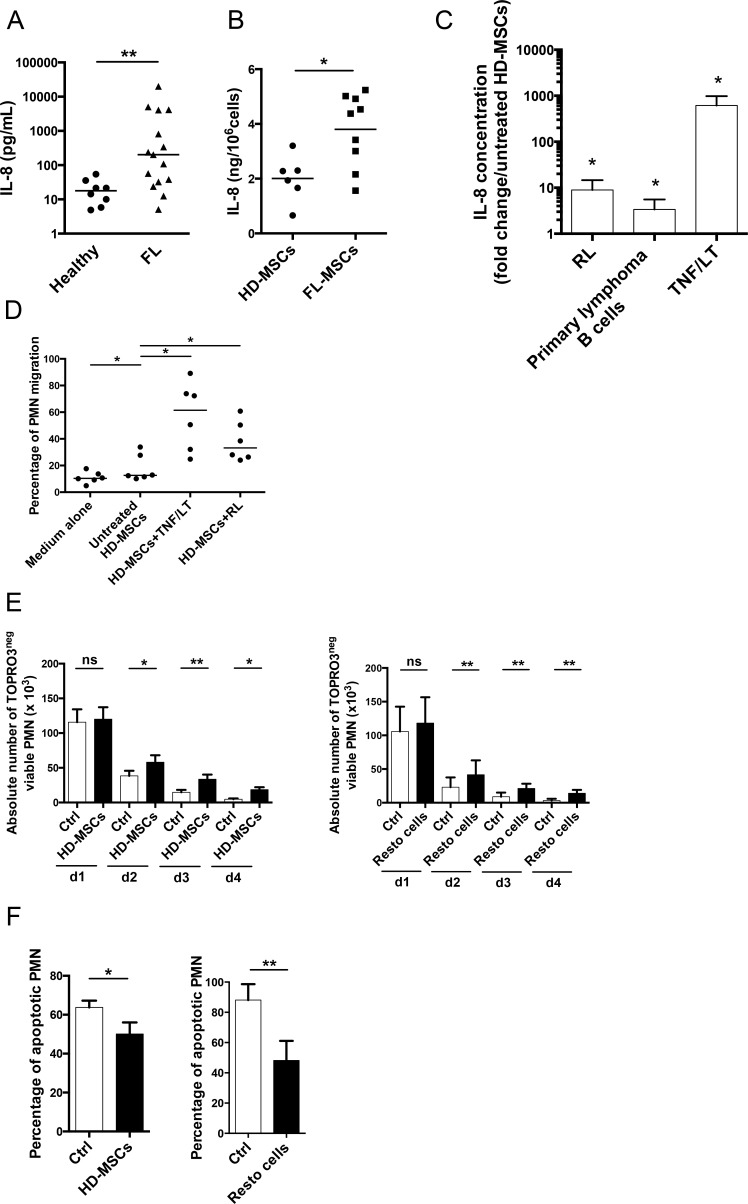
Infiltrating stromal cells recruit neutrophils and protect them from apoptosis (**A**) IL-8 was quantified by ELISA in the BM plasma obtained from HD (n=8) and FL patients (n=15). ** *P* < 0.01. (**B**) IL-8 production in culture supernatants from HD-MSCs (n=6) and FL-MSCs (n=9) was quantified by ELISA at the end of P1. Data are normalized by the number of cultured MSCs. * *P* < 0.05. (**C**) HD-MSCs were stimulated for 3 days by TNF/LT (n=5) or were co-cultured with RL (n=6), or with purified primary FL B cells (n=6). IL-8 was measured in cell supernatants by ELISA. Results are expressed as the mean fold change ± SD compared with untreated HD-MSCs.* *P* < 0.05 (**D**) Migration of neutrophils (PMN) in response to medium alone, supernatants of HD-MSCs stimulated or not by TNF/LT for 3 days, or supernatants of HD-MSCs maintained during 3 days in coculture with RL. The percentage of neutrophil migration is calculated as the number of TOPRO-3^neg^CD66b^pos^ viable neutrophils migrating in response to cell supernatant divided by their initial number. Results represent the mean ± SD from 6 experiments. * *P* < 0.05. (**E**) Purified neutrophils were cultured alone (Ctrl), in the presence of HD-MSCs (n=6, left), or Resto cells (n=9, right). The absolute number of TOPRO-3^neg^CD66b^pos^CD105^neg^viable neutrophils was assessed using FlowCount beads. * *P* <0.05; ** *P* < 0.01. (**F**) Percentage of CD66b^pos^CD105^neg^ neutrophil apoptosis was evaluated at day 1 by the use of active caspase-3 staining for neutrophils cultured alone (Ctrl), in the presence of HD-MSCs (n=6, left), or Resto cells (n=9, right). * *P* < .05; ** *P* < .01.

IL-8 is the major neutrophil recruiting chemokine and is overexpressed by malignant cells in several solid tumors [3]. Strikingly, whereas neither GC-derived B cell lines nor FL and DLBCL primary B cells secreted IL-8 (data not shown), IL-8 was significantly increased in FL-invaded BM plasma compared with normal BM plasma (202.4 pg/mL [7.5-20.000] *vs* 17.9 pg/mL [7.5-54.2]; Figure 2A). We have previously demonstrated that FL-MSCs displayed a specific GEP making them a useful tool to study lymphoma-driven alterations of stromal microenvironment [11]. As described for CCL2, IL-8 was produced at a higher level in the supernatant of BM FL-MSCs, known to be ectopically committed to lymphoid stroma [11], compared with BM HD-MSCs (Figure 2B). We next tried to evaluate whether malignant B cells could contribute to this overexpression of IL-8 by FL-MSCs. For that purpose, HD-MSCs were cultured with TNF/LT, a well-known inducer of lymphoid stroma differentiation [17], or with primary FL B cells and RL cell line. This experiment revealed an increase in IL-8 secretion in the presence of malignant B cells (Figure 2C). Finally, we explored the functional relevance of such IL-8 induction and pinpointed that conditioned media from HD-MSCs treated by TNF/LT or primed by malignant B cells recruited more efficiently purified neutrophils than unstimulated HD-MSC supernatant (Figure 2D).

BM-MSCs were recently reported to support neutrophil survival *in vitro* [7, 8]. We validated here this result by demonstrating a significant increase in the number of viable neutrophils after 2 to 4 days of culture in the presence of BM-MSCs. Moreover, we extended these data to tonsil-derived Resto stromal cells (Figure 2E). This supportive activity was associated with a decrease in neutrophil apoptosis (Figure 2F). Of note, similar results were obtained with HD-MSCs and FL-MSCs (Supplemental Figure S2). These data indicated that although lymphoma-infiltrating stromal cells produced higher levels of IL-8, probably in part through contact with malignant B cells, various stromal cell subsets could trigger neutrophil survival.

### Neutrophils and stromal cells cooperate to trigger malignant B-cell growth

Since BM and tonsil-derived stromal cells triggered neutrophil recruitment and survival, we decided to test the tripartite co-culture between stromal cells, neutrophils, and B cells.

**Figure 3:**
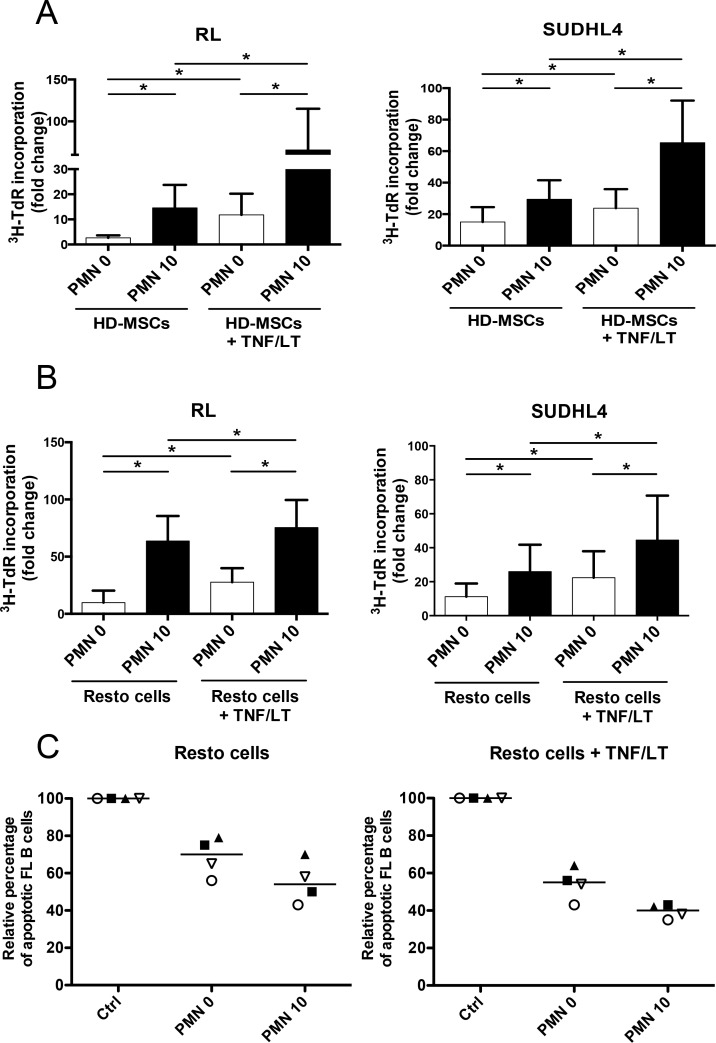
Neutrophils and stromal cells cooperate to sustain malignant B-cell growth (**A-B**) GC-derived B-cell lines were cultured in low serum concentration with BM-MSCs (**A**) or tonsil Resto cells (**B**) in the presence or not of neutrophils (PMN). Stromal cells were pretreated or not with TNF/LT before co-culture. B-cell growth was evaluated by tritiated thymidine (^3^H-TdR) incorporation at day 2 for RL and at day 3 for SUDHL4. Stromal cells cultured alone always showed a ^3^H-TdR incorporation <500 cpm. ^3^HTdR incorporation of B-cell alone was assigned to 1. Results represent the mean ± SD from 6 to 7 experiments. * *P* < 0.05. (**C**) Purified FL B cells were cultured with or without (Ctrl) Resto cells in the presence or not of PMN. Resto cells were pretreated or not with TNF/LT before co-culture. B-cell apoptosis was evaluated by determining the percentage of active caspase-3^pos^CD19/CD20^pos^CD66b^neg^ B cells at day 2 and compared to that of FL B cells cultured alone, arbitrary assigned to 100%. Each symbol corresponds to an individual patient sample. Bars: median.

Neutrophils cooperated with both HD-MSCs and Resto cells to promote the growth of malignant B-cell lines (Figure 3A-3B). As previously reported, treatment of stromal cells with TNF/LT reinforced their B-cell supportive capacity through commitment into functional lymphoid stroma [17]. Interestingly, combination of neutrophils and TNF/LT-stimulated stromal cells also improved B-cell growth compared to neutrophils or activated stromal cells alone. Since neutrophils were not able to directly sustain FL B-cell growth *in vitro* but were found in close contact with FL lymphoid stromal cells *in situ*, we tested how neutrophil/stromal cell cooperation could favor primary FL B-cell survival. Importantly, neutrophils increased the anti-apoptotic activity of both resting and TNF/LT-pretreated stromal cells towards purified FL B cells (Figure 3C), suggesting that such crosstalk could favor the organization of a fully supportive FL cell niche.

### Neutrophils drive stromal cells into a B-cell supportive inflammatory lymphoid stroma phenotype

**Figure 4:**
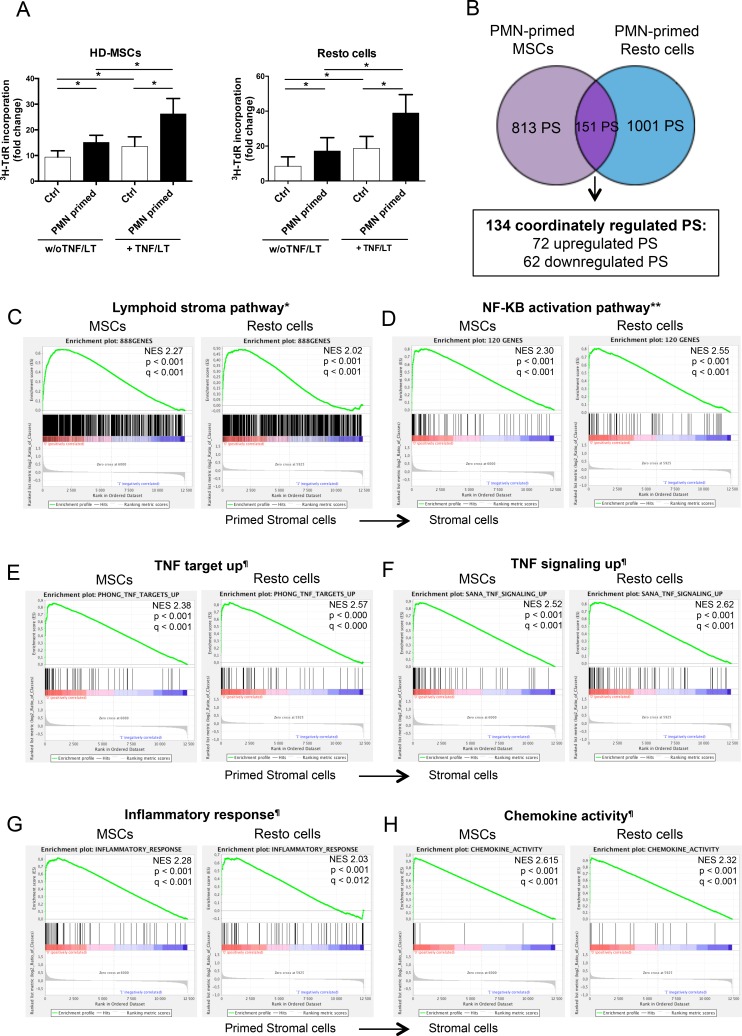
Neutrophils induce a specific B-cell supportive inflammatory profile in stromal cells (**A**) RL cell line was cultured in low serum concentration in the presence of BM-MSCs or tonsil Resto cells primed or not (Ctrl) by neutrophils (ratio stromal cells/neutrophils: 1:1.5) and/or TNF/LT before co-culture. RL cell growth was evaluated by tritiated thymidine (^3^H-TdR) incorporation at day 2. Stromal cells cultured alone always showed a ^3^H-TdR incorporation <500 cpm. ^3^HTdR incorporation of B-cell alone was assigned to 1. Results represent the mean ± SD from 6 experiments. * *P* < 0.05. (**B**) Schematic representation of the statistical analysis used to highlight the minimal neutrophil-primed stromal cells signature defined as the intersection of the 2 gene lists obtained for neutrophil-primed MSCs (964 probesets) and neutrophil-primed Resto cells (1152 probesets) by paired *t*-test (absolute log_2_ fold change >1.2 and *P*< 0.05). (**C–H**) Selected plots from a GSEA based on the comparison of neutrophil-primed *versus* unprimed stromal cell signatures. Normalized enrichment score (NES), nominal *p*-value (p), and FDR (q) are given for each plot. Primed stromal cells are shown on the left (red) of the plots, normal stromal cells on the right side (blue). * TNF/LT lymphoid stroma signature [11], ** NF-κB target gene set [53], ¶MySigDB gene set (GSEA software).

Neutrophil half-life is short compared to that of resident stromal cells *in vivo*, leading us to explore the hypothesis that neutrophils and stromal cells established a bidirectional crosstalk where neutrophils newly recruited by stromal cells contributed in turn to their polarization. Priming of stromal cells by neutrophils increased their capacity to further support B-cell growth at a similar level than activation by TNF/LT (Figure 4A). In addition, neutrophils exhibited a strong activity on TNF/LT-conditioned stroma, mimicking FRC lymphoid cells, and converted them into highly powerful malignant B-cell supportive cells.

To understand the molecular basis of this neutrophil-dependent priming of stromal cells, we decided to study the GEP of both BM-MSCs and Resto cells after co-culture with neutrophils. Importantly, no residual neutrophil, as validated by RQ-PCR for the neutrophil specific gene *ELANE*, was detected in primed stromal cell samples after neutrophil removal. We underlined 3 signatures: the neutrophil-primed MSC signature comprising 964 probesets (PS), the neutrophil-primed Resto signature comprising 1152 PS, and the neutrophil-primed stroma signature obtained by comparing the 6 unprimed stromal cell samples to the 6 neutrophil-primed stromal cell samples and comprising 577 PS (Supplemental Tables S1-S3). Among the 151 PS that were simultaneously found with in the neutrophil-primed MSC signature and the neutrophil-primed Resto signature (Figure 4B), 134 were coordinately up-regulated or down-regulated (Supplemental Table S4).

**Figure 5:**
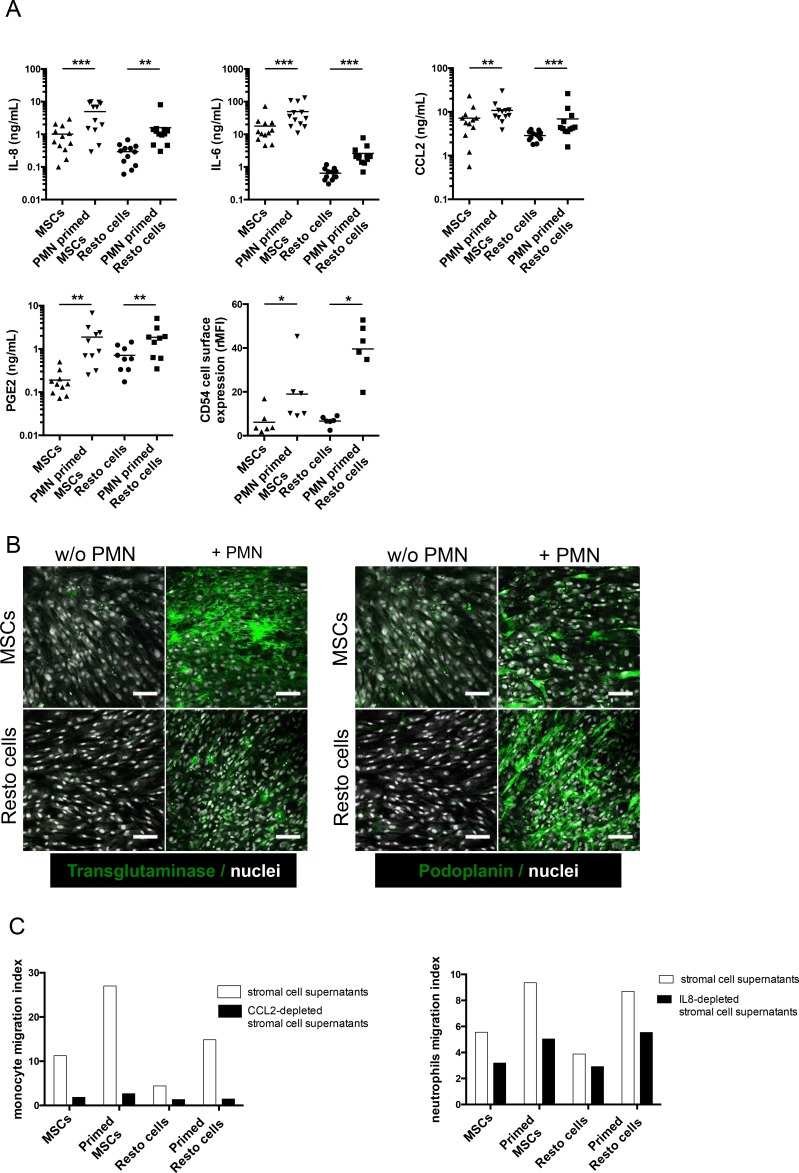
Functional validation of the inflammatory lymphoid stroma phenotype triggered by neutrophils in stromal cells (**A**) Production of IL-8, IL-6, CCL2, and PGE2 was evaluated by ELISA in the supernatant of stromal cells primed or not with neutrophils (PMN) for 3 days before neutrophil removal and additional 3-day culture of stromal cells alone (n = 10 to 12). CD54 expression was evaluated by flow cytometry on stromal cells primed or not with neutrophils as the ratio of mean fluorescence intensity (rMFI) compared with isotype control. * *P* < 0.05; ** *P* < .001; *** *P* < 0.001. (**B**) Stromal cells were cultured for 3 days with or without neutrophils before fixation and transglutaminase and podoplanin staining. Scale bars, 100 μm. (**C**) Migration of purified peripheral blood monocytes (left) or neutrophils (right) in response to supernatants of stromal cells primed or not with neutrophils and specifically depleted or not from CCL2 or IL-8 with magnetic beads. Cell migration index is calculated as the number of DAPI^neg^CD14^pos^ (monocytes) or DAPI^neg^CD66b^pos^ (neutrophils) viable cells migrating in response to cell supernatant divided by their numbers in response to migration medium. Shown is one representative experiment out of 3.

We next performed gene set enrichment analysis (GSEA) approach based on previously published signatures to highlight the major pathways activated by neutrophils in stromal cells. Interestingly, neutrophil-primed MSCs and Resto cells showed signs of lymphoid stroma polarization and inflammatory response, with a strong activation of TNF and NF-κB signaling pathways (Figure 4C-4H). Among the most strongly up-regulated genes in neutrophil-primed stromal cells we selected a set of 5 genes previously described as overexpressed in human lymphoid stromal cells [11, 31] (Table 1) and confirmed the significant overexpression of *IL-8, IL-6, CCL2, PTGS2/COX-2*, and *ICAM* by RQ-PCR in both neutrophil-primed MSCs and neutrophil-primed Resto cells (Supplemental Figure S3A). Accordingly, we demonstrated that stromal cells primed with neutrophils for 3 days before neutrophil removal and maintained again in culture for 3 days secreted significantly higher levels of IL-8 (6.4-fold for MSCs and 3.8-fold for Resto cells), IL-6 (2.9-fold for MSCs and 3.5-fold for Resto cells), CCL2 (1.5-fold for MSCs and 1.5-fold for Resto cells), and PGE2 (6.5-fold for MSCs and 1.9-fold for Resto cells) than their unstimulated counterparts (Figure 5A). They also displayed a stronger membrane expression of CD54 (4-fold for MSCs and 6-fold for Resto cells). To definitively conclude on the lymphoid stroma polarization of neutrophil-primed stromal cells, we checked for their capacity to secrete and organize a meshwork of transglutaminase-positive fibers and to express podoplanin/gp38, two well-described markers of human FRCs. Neutrophil-primed stromal cells produced a dense transgutaminase-positive extracellular reticular network with a very similar pattern to that obtained following FRC-commitment by TNF/LT [17], and expressed high levels of podoplanin (Figure 5B). Of note, as previously reported after TNF/LT-mediated activation [17], transglutaminase expression was not regulated at the transcriptional level in stromal cells by neutrophils (data not shown). However, *PDPN* was significantly induced in both MSCs and Resto cells after priming by neutrophils (Supplemental Figure S3B).

**Table 1:** Expression of lymphoid stromal cell genes in primed versus unprimed stromal cell subsets

Gene Symbol	Primed MSCs		Primed Resto cells		Primed stromal cells^§^	
	P-value	Fold Change*	P-value	Fold Change*	*P*-value	Fold Change*
*IL8*	0.027	205.7	0.011	114.6	6.64e-005	153.6
*IL6*	ns (0.058)	7.1	0.029	17.4	0.006	11.1
*CCL2*	0.006	7.8	0.046	7.2	0.002	7.5
*PTGS2*	ns (0.150)	7.4	0.021	2.7	0.021	4.0
*ICAM1*	0.013	16.7	0.004	30.7	1.49e-005	14.2

* Fold Change corresponds to the ratio of median expression in neutrophil-primed / unprimed stroma

§ Primed stromal cells represent the data obtained by comparing the group of neutrophil-primed MSCs and Resto cells to the group of unprimed MSCs and Resto cells.

We next sought to determine whether these neutrophil-primed FRC-like cells became fully competent not only as tumor B-cell feeders, but also as organizers of lymphoma B-cell niche. We decided to focus on monocyte and neutrophil migration since FL-MSCs have been shown to recruit both cell subsets more efficiently than HD-MSCs. We first revealed that supernatants of neutrophil-primed MSCs and Resto cells triggered an improved chemotaxis of monocytes and neutrophils, compared to resting stromal cells (Figure 5C). To ascertain whether CCL2 and IL-8 contributed to stroma-dependent myeloid cell migration, we specifically depleted them from stromal cell conditionned media. Interestingly, CCL2 and IL-8 depletion strongly decreased monocyte and neutrophil recruitment, respectively.

Taken together, these results support the hypothesis that neutrophil-primed stromal cells were engaged toward lymphoid stroma differentiation, in association with an increased capacity to support malignant B-cell growth and to trigger monocyte and neutrophil recruitment.

### Neutrophil-dependent stroma polarization is associated with NF-κB activation and NETosis

**Figure 6:**
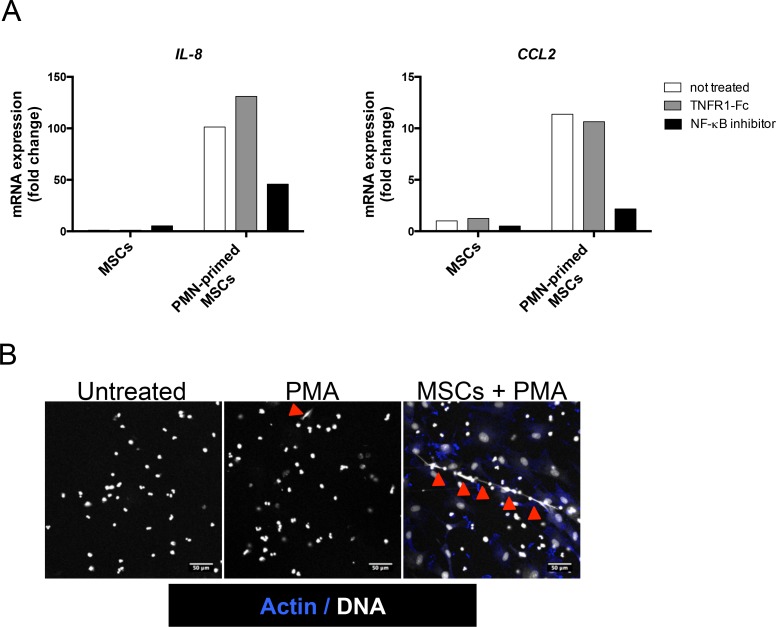
NF-κB pathway is involved in neutrophil-dependent stromal cell priming (**A**) MSCs were cultured with TNFR1-Fc or wedelolactone in the presence or not of neutrophils (PMN) for 1 day and RQ-PCR was performed on stromal cells to analyze *IL8*, and *CCL2* expression. Each sample was normalized to *PUM1* expression and compared to expression levels in untreated MSCs. Shown is one representative experiment out of 3. (**B**) Neutrophils were cultured with or without MSCs for 12h and thereafter treated or not for 3h with PMA before staining of actin with phalloidin (blue) and DNA with Sytox (white). Arrows indicate NET formation. Scale bars, 50 μm.

Since the GEP of neutrophil-activated stromal cells revealed enrichment for genes belonging to NF-κB and TNF signaling pathways, we decided to evaluate the role of NF-κB activation and TNF in neutrophil-dependent stroma polarization. We validated that TNFR1-Fc completely abrogated the upregulation of *IL-8* and *CCL2* induced by addition of exogeneous TNF (data not shown). Interestingly, inhibition of IKK by wedelolactone, unlike the specific blockade of TNFR1, strongly reduced the neutrophil-mediated induction of *IL-8* and *CCL2* in stromal cells (Figure 6A).

Recently, neutrophils were reported to trigger NF-κB activation in CD5^pos^ B cells in a mouse model of chronic lymphocytic leukemia and this activation relied on their capacity to undergo NETosis [32]. We thus tested whether MSCs could influence NETosis. For that purpose, freshly isolated neutrophils were seeded onto poly-D-lysine (a permissive substrate for NET) and stimulated by a low dose of PMA, a well-known NET inducer. Interestingly, whereas MSCs did not directly promote NET formation, they favored PMA-triggered NETosis (Figure 6B).

## DISCUSSION

In this paper, we highlighted for the first time both direct and indirect tumor promoting effects of neutrophils in GC-derived B-cell lymphomas, a group of diseases in which TAN have been poorly studied even if microenvironment is supposed to play a key role. We first extended a previous work revealing that neutrophil-derived APRIL is concentrated within DLBCL cell niches [27] by showing that neutrophils could favor DLBCL B-cell growth *in vitro*, in a BAFF/APRIL dependent manner. We could not detect any activity of soluble exogenous BAFF or APRIL on malignant B cells and direct neutrophil/B-cell contact was required. Since APRIL, unlike BAFF, requires concentration and aggregation by heparan sulfate proteoglycans to be active and is poorly detected in DLBCL peripheral blood samples despite a high concentration at the TAN/B-cell interface inside tumors [27], it is tempting to speculate that APRIL plays a preeminent role over BAFF in this system, thus arguing for the development of APRIL antagonist mAbs for the treatment of B-cell lymphomas [33].

In FL samples, we could not detect a direct effect of neutrophils on B-cell survival but neutrophils and stromal cells cooperate to inhibit FL B-cell apoptosis. FL-TAN exhibited essentially perifollicular localization in close contact with FRC, raising the question of the recruitment and role of these peritumoral neutrophils that have been proposed as promoters of angiogenesis in hepatocellular carcinomas [34]. Interestingly, FRC meshwork is expanded and activated within FL-invaded LN, and FL BM infiltration is characterized by the ectopic development of lymphoid-like stromal cells that form aggregates with malignant B cells [16]. The mechanisms underlying such activation of tumor-infiltrating stroma remain incompletely understood. We have previously demonstrated that FL-MSCs overexpressed CCL2 in response to B-cell derived TNF [11] and identified here IL-8 as another marker of tumor-educated MSCs. These data argue for a crucial role for malignant cells in stroma activation. Of note, PGE2 is also produced at higher level by FL-infiltrating stromal cells in LN and BM but the mechanism of this overexpression is unknown [35]. Importantly, CCL2, IL-8, and PGE2 were all induced by the crosstalk between neutrophils and stromal cells. Moreover, neutrophils were able to convert tonsil and BM-derived stromal cells into lymphoid stroma with transcriptomic, phenotypic, and functional features of FRC-like cells, a property that has been only described until now for lymphoid precursors, some mature CD4^pos^ T cells, and malignant B cells [17, 36, 37]. These results suggest that non-malignant cells, in particular TAN, contribute to the acquisition of lymphoma-specific stroma features, as recently proposed in gastric cancer, where neutrophils favor the transition of MSCs towards CAF-like cells promoting gastric cell migration [9]. Conversely, as already reported for BM-MSCs [7, 8], we confirmed that LN and BM stromal cells support survival of neutrophils. Besides this anti-apoptotic activity, CAFs have been recently demonstrated to activate neutrophils in both solid tumors and lymphoid malignancies. In particular, based on a genetically-induced remodeling of LN stroma in autoimmune prone mice, an elegant study has underlined how stromal cells could trigger lymphoma transformation by increasing TAN activation [32]. Moreover, such reshaping of stroma architecture, induced by a loss of expression of the extracellular matrix protein SPARC, was detected *in situ* in early-stage FL, suggesting that neutrophil/stromal cell/B cell tripartite interactions take place within FL cell niche.

The molecular mechanisms of the neutrophil-mediated stroma activation clearly involved NF-κB pathway, as highlighted by the transcriptomic analysis, and by the inhibition of CCL2 and IL-8 expression by IKK inhibitor. It has long been assumed that NF-κB is the central effector of lymphoid stroma differentiation in normal and pathological settings [36]. More surprisingly, blockade of TNFR1 did not abrogate the effect of neutrophils on stromal cells, suggesting that other NF-κB-activating molecules could be involved. As an example, neutrophils have been described to express functional CD40L [23] whereas CD40 is detected on inflammatory stromal cells [38]. However, neutrophils also induced a strong expression of TNF in stromal cells (data not shown) and such autocrine loop is very difficult to inhibit by blocking antibodies. We could thus not exclude an involvement of stroma-derived TNF in the lymphoid stroma differentiation process. Another interesting observation was that stromal cells sensitized neutrophils to activation-induced NETosis. NET formation has been associated with the capacity of neutrophils to activate NF-κB pathway in chronic lymphocytic leukemia B cells [32]. It is thus tempting to speculate that NET also contributes to neutrophil-mediated activation of NF-κB in stromal cells. Activated platelets were recently reported to simultaneously protect neutrophils from apoptosis and prime them for NET through expression of HMGB1 [39]. How stromal cells favor both neutrophil survival and extrusion of NET is a key unsolved issue.

Bidirectional crosstalk between stromal cells and neutrophils has several tumor-promoting functional consequences. First, neutrophil-primed stromal cells supported more efficiently the growth of malignant B cells, a property shared with stromal cells committed towards lymphoid stroma by TNF/LT or by co-culture with malignant B cells [17]. We previously demonstrated that stromal cells and macrophages synergized to promote lymphoma cell growth [11] and our current data reveal that such cell collaboration is also effective between stromal cells and neutrophils. Second, neutrophil-primed stromal cells produced high amounts of CCL2 and IL-8 that triggered monocyte and neutrophil recruitment, respectively. Multiple lines of evidence support a role for TAM in the biology of GC-derived lymphoma, in particular through their capacity to produce BAFF and IL-15 [40, 41]. A high number of STAT1^pos^ FL-TAM is associated with an adverse outcome [42] and STAT1 activation triggers CCR2 induction in macrophages [43] thus favoring their recruitment by stromal CCL2. Neutrophils could thus contribute to the stroma/macrophage crosstalk. Moreover, CCL2, IL-8, and PGE2 have been shown to inhibit neutrophil apoptosis [3, 44]. Finally, neutrophils could also participate to the development of an immunosuppressive microenvironment in B-cell lymphomas. TGF-β is overexpressed in B-cell lymphomas and contributes to effector memory T cell exhaustion [45]. TGF-β is usually sequestered as an inactive complex, and becomes activated through enzymes and reactive oxygen free radicals, all produced by activated neutrophils [3]. Besides IL-8, several other ligands of CXCR2, including *CXCL1*, *CXCL3*, and *CXCL5*, were induced by neutrophils in stromal cells whereas macrophage migration inhibitory factor (MIF) expression was not modulated. Interestingly, *CXCL5* is also upregulated during lymphoid stroma differentiation [11]. These chemokines could thus contribute to the stroma-dependent migration of neutrophils that was only partly inhibited by IL-8-depletion. Altogether, neutrophils display a wide range of tumor-supporting activity through their interaction with stromal cells and this crosstalk should be considered, in addition to the previously characterized TAM/stroma crosstalk, for the design of new therapeutic strategies.

One of the most interesting feature of FL-TAM is their opposite predictive value depending on the treatment schedule. In fact, FL-TAM could favor tumor progression but also contribute to the clinical efficacy of antibody-based anti-lymphomadrugs [14]. Whether such dual activity exists for lymphoma-infiltrating neutrophils has not been precisely explored. However, besides the well-known roles of NK and macrophages in anti-CD20 antibody activity, neutrophils have already been shown to contribute to the antitumor efficacy of Rituximab [46] and more recently of obinutuzumab through CD16B-dependent phagocytosis [47]. In addition, anti-CD20 IgA have been proposed as highly efficient alternative to classical IgG1 in particular through their capacity to trigger lymphoma cell phagocytosis by CD89-expressing neutrophils [48]. Finally, both GM-CSF and G-CSF, two neutrophil growth factors, have been suggested to improve rituximab efficacy in relapsed/refractory low-grade lymphoma patients [49, 50]. A better understanding of the role of TAN in the intricate network of cell interactions that drives B-cell lymphoma survival, growth, and drug resistance/sensitivity is a highly relevant issue with potential clinical consequences for the design of new anti-lymphoma approaches.

## MATERIALS AND METHODS

### Patient samples and cell lines

Samples came from subjects recruited under institutional review board approval and informed consent process according to the Declaration of Helsinki. BM aspirates were obtained from FL patients at diagnosis and age-matched patients undergoing cardiac surgery, tonsils from children undergoing routine tonsillectomy, LN biopsies from FL and DLBCL patients, and peripheral blood (PB) from adult healthy volunteers. BM plasma were collected by centrifugation and frozen until use. BM-MSCs and tonsil-derived stromal cells (Resto) were obtained as previously described [11, 17] and used for functional studies at passages 1 to 3 and 8 to 15; respectively. Peripheral blood (PB) neutrophils, PB monocytes, and malignant B cells from frozen FL and DLBCL biopsies were purified using whole blood CD15 microbeads, monocyte isolation kit II, and B-cell isolation kit II (Miltenyi Biotech), respectively. Purified cell fractions with at least 98% cell purity as evaluated by flow cytometry were used for further experiments. GC-derived lymphoma B-cell lines RL, SUDHL4, and DOHH2 were obtained from the DSMZ. Classification of DLBCL samples into GCB versus non-GCB subtypes was performed as described [51].

### Antibodies and flow cytometry

FITC-conjugated monoclonal antibodies (mAbs) to CD14, CD19, CD20, and CD66b; PE-conjugated mAbs to CD19, CD20, CD54, and CD105; PC7-conjugated mAbs to CD19, and CD20; and PB-conjugated mAbs to CD15 were provided by Beckman Coulter. Alexa-647-cojugated mAb to CD105 was provided by ebioscience. Appropriate isotype-matched mAbs were used as negative controls and all analyses were performed using a Gallios (Beckman Coulter) flow cytometer. Cell death was checked using TOPRO-3 or DAPI (Life Technologies) staining. Apoptosis evaluation was performed using PE-conjugated active caspase-3 apoptosis kit (Becton Dickinson).

### B-cell growth and apoptosis

After serum deprivation, RL and SUDHL4 were seeded with low serum concentration in the presence of purified neutrophils and/or on a confluent stromal cell monolayer. B-cell growth was evaluated by tritiated thymidine incorporation or by counting the number of viable B cells using FlowCounts beads (Beckman Coulter). B-cell apoptosis was analyzed by the use of active caspase-3 staining. When indicated, neutrophils and B cells were separated by a 0.4-μm pore Transwell filter or were co-cultured in the presence of polyclonal human immunoglobulin (Tegeline^®^, LFB Biomedicaments) and TACI-Fc inhibitor (100 ng/mL, R&D Systems). Stromal cells were pretreated with TNF-α (TNF, 20 ng/mL) and lymphotoxin-α1β2 (LT, 100 ng/mL; R&D Systems) and/or primed with neutrophils before washing and co-culture with B cells. For primary lymphoma samples, purified malignant B cells were seeded in the presence or not of stromal cells and/or neutrophils before analysis of B-cell death.

### Cytokine quantification

IL-8 was quantified by ELISA (R&D Systems) in BM plasma, in the supernatants of HD-MSCs and FL-MSCs collected at the end of passage 1, and in the supernatants of HD-MSCs stimulated with TNF/LT or cocultured with RL or purified FL B cells for 3 days. In addition, we measured by ELISA IL-8, IL-6, CCL2 (R&D Systems) and PGE2 (Cayman Chemical) levels in the supernatants of stromal cells primed or not by neutrophils for 3 days before neutrophil removal and additional 3-day culture period. To confirm the stromal origin of secreted cytokines after priming with neutrophils, we checked by RQ-PCR that the mRNA level of *ELANE*, the neutrophil-specific elastase coding gene, was negative after neutrophil removal. When indicated, inhibitors of NF-κB pathway were used during the priming of stromal cells by neutrophils, including TNFR1-Fc (100 ng/mL, R&D Systems) or wedelolactone (50 μM, Calbiochem). The dose of wedelolactone was chosen as efficient to inhibit NF-κB signaling [52] without any cytotoxic effect on stromal cells.

### Migration assays

Purified PB neutrophils or monocytes (10^5^ cells/100μL) were added in RPMI-0.1% human serum albumin (neutrophil migration medium) or RPMI-1% FCS (monocyte migration medium) to the upper compartment of Transwell chambers with 5-μm pore filters. Lower chambers contained supernatants of stromal cells obtained after 3 days of priming by TNF/LT, RL, or neutrophils. For IL-8 and CCL2 depletion, 2.10^7^ Dynabeads pan mouse IgG (Life Technologies) were conjugated to 10 μg anti-IL-8 or anti-CCL2 mAbs (R&D Systems) before incubation with supernatants. Supernatants were thereafter seeded inside a magnetic field to allow immune complex retention. We checked by ELISA that IL-8 or CCL2 were undetectable in corresponding depleted supernatants. Conversely, the use of an isotype-matched control mAb did not modify the concentration of IL-8 or CCL2. The absolute number of DAPI^neg^CD66b^pos^ viable neutrophils or DAPI^neg^CD14^pos^ viable monocytes was quantified using FlowCount beads in the lower chamber after 1 and 2 hours, respectively.

### Neutrophil survival and apoptosis

Purified neutrophils were cultured alone or in the presence of stromal cells. Cell apoptosis was analyzed using active caspase-3 staining gated on CD66b^pos^CD105^neg^ neutrophils, and the number of TOPRO-3^neg^CD66b^pos^CD105^neg^ viable neutrophils was determined each day for 4 days.

### Gene expression profiling (GEP) study

GEP was performed on 3 HD-MSCs and 3 tonsil-derived Resto cells primed or not with neutrophils for 1 day (ratio 1:1.5). RNA was extracted after neutrophils removal by extensive washes, using RNeasy Mini Kit, (Qiagen) and the purity and integrity of RNA were checked with the Bioanalyzer 2100 (Agilent Technologies). We checked for the absence of residual neutrophils by RQ-PCR for *ELANE*. Biotinylated cRNA were amplified according to the IVT express labeling protocol and hybridized on GeneChip HG-U133 Plus2 oligonucleotide microarrays (Affymetrix). Microarray data were deposited at NCBI GEO data set under accession number GSE62782. Data were analyzed with the Partek Genomics Suite (Partek). Expression signal values and *P*-values were obtained for each probeset (PS) using the Partek Genomics Suite software (Partek) after normalization by Robust Multichip Averaging algorithm with GC content adjustment (GC-RMA). Background noise was decreased by eliminating PS with a low standard deviation to mean ratio. To compare stromal cells primed or not by neutrophils, supervised analysis were carried out by paired Student *t*-test with Partek Genomics Suite software allowing the selection of PS with absolute log_2_ fold change >1.2 and an adjusted *P*-value less than 0.05. Gene Set Enrichment Analysis (GSEA) software was used to determine the specific enrichment of specific gene sets in the PMN-primed stroma signatures.

### Real-time quantitative PCR

An additional set of neutrophil-stromal cell coculture experiments was performed for validation of GEP data and RNA was generated from resting and neutrophil-primed stromal cells. cDNA synthesis was performed with the Superscript II reverse transcriptase and random hexamers (Life Technologies). For quantitative RQ-PCR, we used assay-on-demand primers and probes, and Taqman Universal Master Mix (Life Technologies). Gene expression was measured using the StepOnePlus (Life Technologies) based on the ΔC_t_ calculation method. *PUM1* was determined as the appropriate internal standard gene using TaqMan Endogenous Control Assays (Life Technologies). Quantification of *TNFRSF13* and *TNFRSF13B* was performed using appropriate assay-on-demand primers and probes and *ABL1* as internal standard gene in GC B-cell lines, purified CD14^pos^ monocytes, and purified CD15^pos^ neutrophils. For each sample, the Ct value for the gene of interest was determined, normalized to its respective value for *ABL1*, and results were then standardized by comparison to gene expression of a pool of five whole tonsil cells.

### Immunofluorescence

BM-MSCs and Resto cells were seeded on chamber coverslips and cultured with or without neutrophils for 3 days before neutrophil removal by extensive washes and staining for transglutaminase and podoplanin [17]. Cells were fixed in 2% paraformaldehyde-PBS and stained with antibodies against transglutaminase and podoplanin (Abcam) followed by labeling with anti-mouse Alexa 488 and anti-rat Alexa 594 secondary antibodies (Jackson Immunoresearch). Coverslips were mounted with mowiol mounting medium including Sytox blue (Life Technologies) and examined using a confocal microscope (SP5X, Leica Microsystem) equipped with a 40 x oil objective. Digital images were processed using ImageJ software.

For NET formation, purified neutrophils were seeded onto slides coated with poly-D-lysine or HD-MSC monolayer for 12 hours before stimulation for 3 hours with 20 ng/ml PMA (Sigma-Aldrich). Cells were fixed with 4% paraformaldehyde-PBS and stained with Texas Red-X Phalloidin (Life Technologies). Coverslips were mounted with mowiol mounting medium including Sytox blue and examined using a confocal microscope.

### Statistical analyses

Statistical analyses were performed with GraphPad Prism 6.0 software using the non-parametric Wilcoxon test for matched pairs or the Mann-Whitney nonparametric U test as appropriate.

## SUPPLEMENTARY MATERIALS TABLES AND FIGURES


