# Supplementary material for: Neutrophils trigger a NF-κB dependent polarization of tumor-supportive stromal cells in germinal center B-cell lymphomas
Source: Oncotarget. 2015 May 12;6(18):16471–87. doi: 10.18632/oncotarget.4106 (PMC4599283; doi:10.18632/oncotarget.4106)
Supplement: Supplementary file 1 [file oncotarget-06-16471-s001.pdf]

Neutrophils trigger a NF-κB dependent polarization of tumor-supportive stromal cells in germinal center B-cell lymphomas

Supplementary Material

Supplemental Table 1: Genes differentially expressed in primed MSC compared with unprimed MSC. Green line indicates genes overexpressed in unprimed MSC and red line indicates genes overexpressed in primed MSC. Fold Change corresponds to the ratio of median expression in PMN-primed / unprimed MSC.

UniGene ID	Probeset ID	Gene Symbol	Gene Title	FoldChange
Hs.507755	215303_at	DCLK1	doublecortin-like kinase 1	-3.54742
Hs.207631	242722_at	LMO7	LIM domain 7	-3.52521
Hs.312485	1555564_a_at	CFI	complement factor I	-3.18608
Hs.728862	216869_at	PDE1C	phosphodiesterase 1C, calmodulin-dependent 70kDa	-3.16192
Hs.480378	228057_at	DDIT4L	DNA-damage-inducible transcript 4-like	-3.0992
Hs.128199	230071_at	sept-11	septin 11	-3.08646
Hs.573143	233261_at	EBF1	Early B-cell factor 1	-3.054
Hs.335079	214577_at	MAP1B	microtubule-associated protein 1B	-3.00739
Hs.623782	1556606_at	NAV2	neuron navigator 2	-2.90344
Hs.477128	225241_at	CCDC80	coiled-coil domain containing 80	-2.77843
Hs.308628	230261_at	ST8SIA4	ST8 alpha-N-acetyl-neuraminide alpha-2,8-sialyltransferase 4	-2.76967
Hs.626544	231183_s_at	JAG1	Jagged 1	-2.73982
Hs.150444	221683_s_at	CEP290	centrosomal protein 290kDa	-2.71977
Hs.469244	224563_at	WASF2	WAS protein family, member 2	-2.53879
Hs.271667	212650_at	EHBP1	EH domain binding protein 1	-2.53303
Hs.405156	209355_s_at	PPAP2B	phosphatidic acid phosphatase type 2B	-2.49452
Hs.128199	201308_s_at	sept-11	septin 11	-2.48958
Hs.644633	201149_s_at	TIMP3	TIMP metallopeptidase inhibitor 3	-2.47042
Hs.121575	227863_at	IFITM10	interferon induced transmembrane protein 10	-2.42733
Hs.532091	203141_s_at	AP3B1	adaptor-related protein complex 3, beta 1 subunit	-2.35341
Hs.292925	219387_at	CCDC88A	coiled-coil domain containing 88A	-2.31477
Hs.234478	228503_at	RPS6KA6	ribosomal protein S6 kinase, 90kDa, polypeptide 6	-2.31252
Hs.430551	213446_s_at	IQGAP1	IQ motif containing GTPase activating protein 1	-2.30405
Hs.521568	239761_at	GCNT1	glucosaminyl (N-acetyl) transferase 1, core 2	-2.29224
Hs.585374	224771_at	NAV1	neuron navigator 1	-2.24463
Hs.490203	201615_x_at	CALD1	caldesmon 1	-2.21587
Hs.292689	201831_s_at	USO1	USO1 vesicle docking protein homolog (yeast)	-2.21552
Hs.600086	223882_at	FAM172A	family with sequence similarity 172, member A	-2.18955
Hs.517941	220946_s_at	SETD2	SET domain containing 2	-2.15987
Hs.203691	213865_at	DCBLD2	discoidin, CUB and LCCL domain containing 2	-2.14964
Hs.190544	1552274_at	PXK	PX domain containing serine/threonine kinase	-2.14798
Hs.593614	220386_s_at	EML4	echinoderm microtubule associated protein like 4	-2.09388
Hs.632604	224037_at	SDAD1	SDA1 domain containing 1	-2.08568
Hs.509736	1557910_at	HSP90AB1	heat shock protein 90kDa alpha (cytosolic), class B member 1	-2.07563
Hs.431101	1555240_s_at	GNG12	guanine nucleotide binding protein (G protein), gamma 12	-2.03325
---	227115_at	LOC100506870	uncharacterized LOC100506870	-2.03306
Hs.527881	210335_at	RASSF9	Ras association (RalGDS/AF-6) domain family (N-terminal) member 9	-2.01308
Hs.368203	226875_at	DOCK11	dedicator of cytokinesis 11	-2.00987
Hs.151220	200906_s_at	PALLD	palladin, cytoskeletal associated protein	-2.00707
Hs.181300	202062_s_at	SEL1L	sel-1 suppressor of lin-12-like (C. elegans)	-1.99982
Hs.592490	203032_s_at	FH	fumarate hydratase	-1.99924
Hs.471119	209920_at	BMPR2	bone morphogenetic protein receptor, type II (serine/threonine kinase)	-1.9852
Hs.728857	1558015_s_at	ACTR2	ARP2 actin-related protein 2 homolog (yeast)	-1.95961
Hs.50749	1554287_at	TRIM4	tripartite motif containing 4	-1.94503
Hs.614080	229115_at	DYNC1H1	dynein, cytoplasmic 1, heavy chain 1	-1.94456
Hs.171054	210602_s_at	CDH6	cadherin 6, type 2, K-cadherin (fetal kidney)	-1.91918
Hs.584933	220022_at	ZNF334	zinc finger protein 334	-1.91467
Hs.438072	214169_at	SUN1	Sad1 and UNC84 domain containing 1	-1.90689
Hs.201858	201151_s_at	MBNL1	muscleblind-like splicing regulator 1	-1.89744
Hs.283869	219633_at	TTPAL	tocopherol (alpha) transfer protein-like	-1.89538
Hs.263671	212398_at	RDX	radixin	-1.89425
Hs.457407	1556695_a_at	FLJ42709	uncharacterized LOC441094	-1.88457

Hs.440168	203498_at	RCAN2	regulator of calcineurin 2	-1.86533
Hs.48589	236328_at	ZFP112 /// ZNF285	zinc finger protein 112 homolog (mouse) /// zinc finger protein 285	-1.85376
Hs.591650	219239_s_at	ZNF654	zinc finger protein 654	-1.85352
Hs.655967	1567032_s_at	ZNF160	zinc finger protein 160	-1.85256
Hs.709187	205371_s_at	DBT	dihydrolipoamide branched chain transacylase E2	-1.84993
Hs.405156	212226_s_at	PPAP2B	phosphatidic acid phosphatase type 2B	-1.84761
Hs.200644	1555980_a_at	LOC100130417	Uncharacterized LOC100130417	-1.84707
Hs.478125	223681_s_at	INADL	InaD-like (Drosophila)	-1.83528
Hs.40582	208022_s_at	CDC14B	CDC14 cell division cycle 14 homolog B (S. cerevisiae)	-1.83325
Hs.732391	219158_s_at	NAA15	N(alpha)-acetyltransferase 15, NatA auxiliary subunit	-1.83278
Hs.515162	212952_at	CALR	Calreticulin	-1.81929
Hs.740523	203532_x_at	CUL5	cullin 5	-1.81703
Hs.731417	219028_at	HIPK2	homeodomain interacting protein kinase 2	-1.81594
Hs.454528	222387_s_at	VPS35	vacuolar protein sorting 35 homolog (S. cerevisiae)	-1.81551
Hs.15106	217188_s_at	C14orf1	chromosome 14 open reading frame 1	-1.8143
Hs.40582	221555_x_at	CDC14B	CDC14 cell division cycle 14 homolog B (S. cerevisiae)	-1.80763
Hs.144795	221583_s_at	KCNMA1	potassium large conductance calcium-activated channel, subfamily M, alpha member 1	-1.80615
Hs.523852	208711_s_at	CCND1	cyclin D1	-1.80159
Hs.592591	203319_s_at	ZNF148	zinc finger protein 148	-1.76211
Hs.531005	241359_at	TLCD2	TLC domain containing 2	-1.75901
Hs.521432	47550_at	LZTS1	leucine zipper, putative tumor suppressor 1	-1.75866
Hs.577256	223701_s_at	USP47	ubiquitin specific peptidase 47	-1.75726
Hs.605153	212382_at	TCF4	transcription factor 4	-1.75427
Hs.8769	209655_s_at	TMEM47	transmembrane protein 47	-1.74602
Hs.511265	226492_at	SEMA6D	sema domain, transmembrane domain (TM), and cytoplasmic domain, (semaphorin) 6D	-1.74576
Hs.593803	1554473_at	SRGAP1	SLIT-ROBO Rho GTPase activating protein 1	-1.74173
Hs.740395	222027_at	NUCKS1	Nuclear casein kinase and cyclin-dependent kinase substrate 1	-1.73919
Hs.525600	211968_s_at	HSP90AA1	heat shock protein 90kDa alpha (cytosolic), class A member 1	-1.73737
Hs.335079	226084_at	MAP1B	microtubule-associated protein 1B	-1.73718
Hs.665307	1555363_s_at	LOC284440	uncharacterized LOC284440	-1.73519
Hs.282998	213901_x_at	RBFOX2	RNA binding protein, fox-1 homolog (C. elegans) 2	-1.73247
Hs.729202	206829_x_at	ZNF430	zinc finger protein 430	-1.72405
Hs.132225	212239_at	PIK3R1	phosphoinositide-3-kinase, regulatory subunit 1 (alpha)	-1.72176
Hs.293970	204290_s_at	ALDH6A1	aldehyde dehydrogenase 6 family, member A1	-1.71783
Hs.371249	209466_x_at	LOC100287705 /// PTN	uncharacterized LOC100287705 /// pleiotrophin	-1.70971
Hs.608111	222544_s_at	WHSC1L1	Wolf-Hirschhorn syndrome candidate 1-like 1	-1.70846
Hs.257970	228278_at	NFIX	nuclear factor I/X (CCAAT-binding transcription factor)	-1.70797
Hs.371856	226897_s_at	ZC3H7A	zinc finger CCCH-type containing 7A	-1.70403
Hs.607943	219685_at	TMEM35	transmembrane protein 35	-1.70007
Hs.101014	203491_s_at	CEP57	centrosomal protein 57kDa	-1.69689
Hs.730695	200760_s_at	ARL6IP5	ADP-ribosylation-like factor 6 interacting protein 5	-1.69575
Hs.593614	228674_s_at	EML4	echinoderm microtubule associated protein like 4	-1.68997
Hs.159066	229399_at	C10orf118	chromosome 10 open reading frame 118	-1.68958
Hs.500775	229765_at	ZNF207	zinc finger protein 207	-1.68866
Hs.728759	217478_s_at	HLA-DMA	major histocompatibility complex, class II, DM alpha	-1.68706
Hs.476517	226697_at	FAM114A1	family with sequence similarity 114, member A1	-1.68629
Hs.167679	217257_at	SH3BP2	SH3-domain binding protein 2	-1.68142
Hs.435369	214505_s_at	FHL1	four and a half LIM domains 1	-1.68084
Hs.532593	243502_at	GJC1	gap junction protein, gamma 1, 45kDa	-1.6793
Hs.331268	1560017_at	TMTC3	transmembrane and tetratricopeptide repeat containing 3	-1.67926
Hs.469473	241017_at	RPL31 /// TBC1D8	ribosomal protein L31 /// TBC1 domain family, member 8 (with GRAM domain)	-1.67066

Hs.435051	210240_s_at	CDKN2D	cyclin-dependent kinase inhibitor 2D (p19, inhibits CDK4)	-1.66753
Hs.461117	205315_s_at	SNTB2	syntrophin, beta 2 (dystrophin-associated protein A1, 59kDa, basic component 2)	-1.66727
Hs.427449	1556427_s_at	LRRN4CL	LRRN4 C-terminal like	-1.65936
Hs.548088	222587_s_at	GALNT7	UDP-N-acetyl-alpha-D-galactosamine:polypeptide N-acetylgalactosaminyltransferase 7 (Gal	-1.65904
Hs.167700	205187_at	SMAD5	SMAD family member 5	-1.65627
---	213267_at	DOPEY1	dopey family member 1	-1.65427
Hs.132257	210458_s_at	TANK	TRAF family member-associated NFKB activator	-1.64729
Hs.654363	243189_at	NRF1	nuclear respiratory factor 1	-1.64067
Hs.312485	203854_at	CFI	complement factor I	-1.63605
Hs.484412	219349_s_at	EXOC2	exocyst complex component 2	-1.63523
Hs.149252	204920_at	CPS1	carbamoyl-phosphate synthase 1, mitochondrial	-1.63365
Hs.476179	201072_s_at	SMARCC1	SWI/SNF related, matrix associated, actin dependent regulator of chromatin, subfamily c	-1.62895
Hs.132858	217457_s_at	RAP1GDS1	RAP1, GTP-GDP dissociation stimulator 1	-1.62634
Hs.647409	202935_s_at	SOX9	SRY (sex determining region Y)-box 9	-1.62604
Hs.211426	204776_at	THBS4	thrombospondin 4	-1.61607
Hs.654519	210078_s_at	KCNAB1	potassium voltage-gated channel, shaker-related subfamily, beta member 1	-1.6154
Hs.102735	210829_s_at	SSBP2	single-stranded DNA binding protein 2	-1.61296
Hs.35086	202412_s_at	USP1	ubiquitin specific peptidase 1	-1.60802
Hs.368944	225798_at	JAZF1	JAZF zinc finger 1	-1.60595
Hs.151220	200907_s_at	PALLD	palladin, cytoskeletal associated protein	-1.6051
Hs.715026	222634_s_at	TBL1XR1	transducin (beta)-like 1 X-linked receptor 1	-1.60472
Hs.529357	240246_at	LOC642236	FSHD region gene 1 pseudogene	-1.60321
Hs.621695	224559_at	LOC100507645 /// MALAT1	uncharacterized LOC100507645 /// metastasis associated lung adenocarcinoma transcript 1	-1.60171
---	213002_at	MARCKS	myristoylated alanine-rich protein kinase C substrate	-1.59628
Hs.317304	225490_at	ARID2	AT rich interactive domain 2 (ARID, RFX-like)	-1.59582
Hs.654616	209790_s_at	CASP6	caspase 6, apoptosis-related cysteine peptidase	-1.59139
Hs.56	209440_at	PRPS1	phosphoribosyl pyrophosphate synthetase 1	-1.58592
Hs.149367	212634_at	UFL1	UFM1-specific ligase 1	-1.58573
Hs.502223	230556_at	IMMP1L	IMP1 inner mitochondrial membrane peptidase-like (S. cerevisiae)	-1.58192
Hs.718875	210251_s_at	RUFY3	RUN and FYVE domain containing 3	-1.58178
Hs.440833	212629_s_at	PKN2	protein kinase N2	-1.57809
Hs.11355	209753_s_at	TMPO	thymopoietin	-1.57769
Hs.497492	225742_at	MDM4	Mdm4 p53 binding protein homolog (mouse)	-1.57714
Hs.11637	243656_at	LOC642852	uncharacterized LOC642852	-1.5765
Hs.301526	242056_at	TRIM45	tripartite motif containing 45	-1.57647
Hs.567832	208663_s_at	TTC3 /// TTC3P1	tetratricopeptide repeat domain 3 /// tetratricopeptide repeat domain 3 pseudogene 1	-1.57368
Hs.674365	220200_s_at	SETD8	SET domain containing (lysine methyltransferase) 8	-1.56664
Hs.389452	221027_s_at	PLA2G12A	phospholipase A2, group XIIA	-1.56299
Hs.533977	201008_s_at	TXNIP	thioredoxin interacting protein	-1.56187
Hs.445030	216048_s_at	RHOBTB3	Rho-related BTB domain containing 3	-1.55988
Hs.148778	223879_s_at	OXR1	oxidation resistance 1	-1.55976
Hs.509343	214212_x_at	FERMT2	fermitin family member 2	-1.55514
Hs.304362	1554472_a_at	PHF20L1	PHD finger protein 20-like 1	-1.55497
Hs.1342	213736_at	COX5B	Cytochrome c oxidase subunit Vb	-1.55313
Hs.158932	203527_s_at	APC	adenomatous polyposis coli	-1.55271
Hs.35433	214464_at	CDC42BPA	CDC42 binding protein kinase alpha (DMPK-like)	-1.5521
Hs.647971	1556787_s_at	PDE5A	Phosphodiesterase 5A, cGMP-specific	-1.55115
Hs.125180	205498_at	GHR	growth hormone receptor	-1.5499
Hs.159161	201167_x_at	ARHGDIA	Rho GDP dissociation inhibitor (GDI) alpha	-1.54985
Hs.263671	204969_s_at	RDX	radixin	-1.54903
Hs.269654	225041_at	MPHOSPH8	M-phase phosphoprotein 8	-1.54888

Hs.613761	205809_s_at	WASL	Wiskott-Aldrich syndrome-like	-1.54828
Hs.725347	235173_at	LOC401093	uncharacterized LOC401093	-1.54678
Hs.520048	210982_s_at	HLA-DRA	major histocompatibility complex, class II, DR alpha	-1.54537
Hs.190544	1552275_s_at	PXK	PX domain containing serine/threonine kinase	-1.54528
Hs.408461	204909_at	DDX6	DEAD (Asp-Glu-Ala-Asp) box helicase 6	-1.54364
Hs.154163	221564_at	PRMT2	protein arginine methyltransferase 2	-1.54097
Hs.192374	200598_s_at	HSP90B1 /// MIR3652	heat shock protein 90kDa beta (Grp94), member 1 /// microRNA 3652	-1.53949
Hs.571177	209024_s_at	SYNCRIP	synaptotagmin binding, cytoplasmic RNA interacting protein	-1.53768
Hs.264482	204833_at	ATG12	autophagy related 12	-1.53695
Hs.25155	201830_s_at	NET1	neuroepithelial cell transforming 1	-1.53356
Hs.144696	237654_at	PPP1R36	protein phosphatase 1, regulatory subunit 36	-1.53262
Hs.216226	210613_s_at	SYNGR1	synaptogyrin 1	-1.53239
Hs.486508	244353_s_at	SLC2A12	solute carrier family 2 (facilitated glucose transporter), member 12	-1.53139
Hs.660232	221218_s_at	TPK1	thiamin pyrophosphokinase 1	-1.52989
Hs.599703	1554512_a_at	CEP89	centrosomal protein 89kDa	-1.52889
Hs.409989	211089_s_at	NEK3	NIMA (never in mitosis gene a)-related kinase 3	-1.5261
Hs.494614	230618_s_at	PRRC2C	Proline-rich coiled-coil 2C	-1.52425
Hs.58992	201663_s_at	SMC4	structural maintenance of chromosomes 4	-1.52193
Hs.567832	1569472_s_at	TTC3 /// TTC3P1	tetratricopeptide repeat domain 3 /// tetratricopeptide repeat domain 3 pseudogene 1	-1.51985
Hs.659873	221884_at	MECOM	MDS1 and EVI1 complex locus	-1.51978
Hs.283416	205991_s_at	PRRX1	paired related homeobox 1	-1.51919
Hs.458986	209741_x_at	SCAPER	S-phase cyclin A-associated protein in the ER	-1.51793
Hs.595933	235030_at	NXPE3	neurexophilin and PC-esterase domain family, member 3	-1.5157
Hs.497575	1568957_x_at	SRGAP2 /// SRGAP2C	SLIT-ROBO Rho GTPase activating protein 2 /// SLIT-ROBO Rho GTPase activating protein 2	-1.51304
Hs.520710	209256_s_at	KLHDC10	kelch domain containing 10	-1.5098
Hs.288773	233819_s_at	LTN1	listerin E3 ubiquitin protein ligase 1	-1.50909
Hs.522752	1554577_a_at	PSMD10	proteasome (prosome, macropain) 26S subunit, non-ATPase, 10	-1.50544
Hs.531106	212028_at	RBM25	RNA binding motif protein 25	-1.50505
Hs.280695	212835_at	FAM175B	family with sequence similarity 175, member B	-1.50235
Hs.656361	1555913_at	GON4L	gon-4-like (C. elegans)	-1.50152
Hs.530597	1568877_a_at	ACBD5	acyl-CoA binding domain containing 5	-1.49981
Hs.444472	216591_s_at	SDHC	succinate dehydrogenase complex, subunit C, integral membrane protein, 15kDa	-1.49899
Hs.371609	228315_at	ZMAT3	zinc finger, matrin-type 3	-1.49879
Hs.319438	206178_at	PLA2G5	phospholipase A2, group V	-1.494
Hs.435775	230051_at	C10orf47	chromosome 10 open reading frame 47	-1.4935
Hs.87889	213229_at	DICER1	dicer 1, ribonuclease type III	-1.49305
Hs.525600	211969_at	HSP90AA1	heat shock protein 90kDa alpha (cytosolic), class A member 1	-1.49163
Hs.408676	213644_at	CEP112	centrosomal protein 112kDa	-1.49067
Hs.371563	211503_s_at	RAB14	RAB14, member RAS oncogene family	-1.48838
Hs.207776	204332_s_at	AGA	aspartylglucosaminidase	-1.48676
Hs.655033	205111_s_at	PLCE1	phospholipase C, epsilon 1	-1.48577
Hs.498890	244835_at	C16orf52	Chromosome 16 open reading frame 52	-1.48517
Hs.445893	201488_x_at	KHDRBS1	KH domain containing, RNA binding, signal transduction associated 1	-1.48225
Hs.435237	205162_at	ERCC8	excision repair cross-complementing rodent repair deficiency, complementation group 8	-1.48155
Hs.658497	231726_at	PCDHB14	protocadherin beta 14	-1.48052
Hs.729380	219785_s_at	C16orf95	chromosome 16 open reading frame 95	-1.47903
Hs.629008	206562_s_at	CSNK1A1	casein kinase 1, alpha 1	-1.47468
Hs.654581	230352_at	PRPS2	Phosphoribosyl pyrophosphate synthetase 2	-1.47442
Hs.740440	219437_s_at	ANKRD11	ankyrin repeat domain 11	-1.47245
Hs.410231	229520_s_at	C14orf118	chromosome 14 open reading frame 118	-1.47005
Hs.498317	222158_s_at	DESI2	desumoylating isopeptidase 2	-1.4697

	Hs.187569	235409_at	MGA	MAX gene associated	-1.46882
	Hs.4998	223078_s_at	TMOD3	tropomodulin 3 (ubiquitous)	-1.46641
	Hs.374201	231875_at	KIF21A	kinesin family member 21A	-1.46359
	Hs.89029	223624_at	ZFAND4	zinc finger, AN1-type domain 4	-1.46172
	Hs.492407	200641_s_at	YWHAZ	tyrosine 3-monooxygenase/tryptophan 5-monooxygenase activation protein, zeta polypeptid	-1.4602
	Hs.702872	216804_s_at	PDLIM5	PDZ and LIM domain 5	-1.4595
	Hs.501012	205882_x_at	ADD3	adducin 3 (gamma)	-1.45827
	Hs.515266	208947_s_at	UPF1	UPF1 regulator of nonsense transcripts homolog (yeast)	-1.45435
	Hs.612385	1552309_a_at	NEXN	nexilin (F actin binding protein)	-1.45427
	Hs.463017	231106_at	BMS1P2 /// BMS1P6	BMS1 pseudogene 2 /// BMS1 pseudogene 6	-1.45332
	Hs.21631	212599_at	AUTS2	autism susceptibility candidate 2	-1.45296
	Hs.130491	203553_s_at	MAP4K5	mitogen-activated protein kinase kinase kinase kinase 5	-1.45006
	Hs.478383	211801_x_at	MFN1	mitofusin 1	-1.44878
	Hs.18616	219588_s_at	NCAPG2	non-SMC condensin II complex, subunit G2	-1.44755
	Hs.440776	224060_s_at	DPH5	DPH5 homolog (S. cerevisiae)	-1.44722
	Hs.654816	212492_s_at	KDM4B	lysine (K)-specific demethylase 4B	-1.44541
	Hs.436166	223464_at	OSBPL5	oxysterol binding protein-like 5	-1.44282
	Hs.593614	223068_at	EML4	echinoderm microtubule associated protein like 4	-1.44244
	Hs.503886	204812_at	ZW10	ZW10, kinetochore associated, homolog (Drosophila)	-1.44125
	Hs.190086	1555978_s_at	MYL12A	Myosin, light chain 12A, regulatory, non-sarcomeric	-1.44086
	Hs.520506	234863_x_at	FBXO5	F-box protein 5	-1.4405
	Hs.396178	1553122_s_at	LOC389458 /// RBAK /// RBAK-LOC389458	uncharacterized LOC389458 /// RB-associated KRAB zinc finger /// RBAK-LOC389458 readthr	-1.44043
	Hs.43697	203348_s_at	ETV5	ets variant 5	-1.4364
	Hs.471234	227280_s_at	CCNYL1	cyclin Y-like 1	-1.43583
	Hs.293970	221588_x_at	ALDH6A1	aldehyde dehydrogenase 6 family, member A1	-1.43493
	Hs.728857	200729_s_at	ACTR2	ARP2 actin-related protein 2 homolog (yeast)	-1.43466
	Hs.12707	1560020_at	DNAJC13	DnaJ (Hsp40) homolog, subfamily C, member 13	-1.43447
	Hs.445030	202975_s_at	RHOBTB3	Rho-related BTB domain containing 3	-1.43409
	Hs.485865	206006_s_at	KIAA1009	KIAA1009	-1.43398
	Hs.508848	200751_s_at	HNRNPC /// LOC100652761 /// LOC100653343	heterogeneous nuclear ribonucleoprotein C (C1/C2) /// uncharacterized LOC100652761 ///	-1.43363
	Hs.30977	226763_at	SESTD1	SEC14 and spectrin domains 1	-1.43334
	Hs.213061	222424_s_at	NUCKS1	nuclear casein kinase and cyclin-dependent kinase substrate 1	-1.43303
	Hs.97627	205761_s_at	DUS4L	dihydrouridine synthase 4-like (S. cerevisiae)	-1.43099
	Hs.2799	205524_s_at	HAPLN1	hyaluronan and proteoglycan link protein 1	-1.43052
	Hs.127310	227740_at	UHMK1	U2AF homology motif (UHM) kinase 1	-1.42781
	Hs.731699	212665_at	TIPARP	TCDD-inducible poly(ADP-ribose) polymerase	-1.42776
	Hs.12326	236076_at	LOC257396	uncharacterized LOC257396	-1.42743
	Hs.519523	1556950_s_at	SERPINB6	serpin peptidase inhibitor, clade B (ovalbumin), member 6	-1.42655
	Hs.124503	212758_s_at	ZEB1	zinc finger E-box binding homeobox 1	-1.42648
	Hs.282998	212104_s_at	RBFOX2	RNA binding protein, fox-1 homolog (C. elegans) 2	-1.42641
	Hs.210367	209376_x_at	SCAF11	SR-related CTD-associated factor 11	-1.42361
	Hs.82098	235369_at	C14orf28	chromosome 14 open reading frame 28	-1.42358
	Hs.436975	225856_at	CLOCK	clock homolog (mouse)	-1.42267
	Hs.490203	201617_x_at	CALD1	caldesmon 1	-1.42108
	Hs.330073	203288_at	KIAA0355	KIAA0355	-1.41934
	Hs.567832	208662_s_at	TTC3 /// TTC3P1	tetratricopeptide repeat domain 3 /// tetratricopeptide repeat domain 3 pseudogene 1	-1.41654
	Hs.674365	225094_at	SETD8	SET domain containing (lysine methyltransferase) 8	-1.41651
	Hs.656558	240155_x_at	ZNF493	zinc finger protein 493	-1.41651
	---	238435_at	LOC100506661	uncharacterized LOC100506661	-1.41599

	Hs.643754	203496_s_at	MED1	mediator complex subunit 1	-1.41397
	Hs.159195	241708_at	DOCK1	dedicator of cytokinesis 1	-1.41297
	Hs.349096	230285_at	SVIP	small VCP/p97-interacting protein	-1.41188
	Hs.405590	235429_at	EIF3E	eukaryotic translation initiation factor 3, subunit E	-1.41114
	Hs.499205	229638_at	IRX3	iroquois homeobox 3	-1.4088
	Hs.720221	230708_at	PRICKLE1	prickle homolog 1 (Drosophila)	-1.40629
	Hs.464585	216563_at	ANKRD12	ankyrin repeat domain 12	-1.40561
	Hs.418192	215892_at	ZNF440	Zinc finger protein 440	-1.40433
	Hs.127535	229893_at	FRMD3	FERM domain containing 3	-1.40252
	Hs.713698	1570523_s_at	ATG10	autophagy related 10	-1.40157
	Hs.491941	231380_at	C8orf34	chromosome 8 open reading frame 34	-1.40144
	Hs.584851	235084_x_at	TRIM38	tripartite motif containing 38	-1.39977
	Hs.486470	201718_s_at	EPB41L2	erythrocyte membrane protein band 4.1-like 2	-1.39912
	Hs.255935	200920_s_at	BTG1	B-cell translocation gene 1, anti-proliferative	-1.39871
	Hs.608041	222024_s_at	AKAP13	A kinase (PRKA) anchor protein 13	-1.39849
	Hs.146804	217813_s_at	SPIN1	spindlin 1	-1.39817
	Hs.632269	226805_at	FITM2	fat storage-inducing transmembrane protein 2	-1.39715
	Hs.406787	218432_at	FBXO3	F-box protein 3	-1.39694
	Hs.425144	205076_s_at	MTMR11	myotubularin related protein 11	-1.39668
	Hs.171929	220988_s_at	C1QTNF3	C1q and tumor necrosis factor related protein 3	-1.39503
	Hs.358997	224908_s_at	TTL	tubulin tyrosine ligase	-1.39441
	Hs.523442	214579_at	NIPAL3	NIPA-like domain containing 3	-1.39439
	Hs.463278	239159_at	GOSR2	golgi SNAP receptor complex member 2	-1.39285
	Hs.677935	226429_at	KIAA1704	KIAA1704	-1.39203
	Hs.340623	228686_at	FLJ33630	uncharacterized LOC644873	-1.39117
	Hs.711490	202731_at	MIR4680 /// PDCD4	microRNA 4680 /// programmed cell death 4 (neoplastic transformation inhibitor)	-1.39108
	Hs.590971	226716_at	PRR12	proline rich 12	-1.39055
	Hs.59425	1554084_a_at	NOL9	nucleolar protein 9	-1.39014
	Hs.730856	206197_at	NME5	NME/NM23 family member 5	-1.38835
	Hs.632616	1554019_s_at	CEP57L1	centrosomal protein 57kDa-like 1	-1.38687
	Hs.670381	1555945_s_at	FAM120A	family with sequence similarity 120A	-1.38652
	Hs.49582	201604_s_at	PPP1R12A	protein phosphatase 1, regulatory subunit 12A	-1.38608
	Hs.439363	1566257_at	GPR180	G protein-coupled receptor 180	-1.38311
	Hs.191540	236254_at	VPS13B	vacuolar protein sorting 13 homolog B (yeast)	-1.38226
	Hs.657382	228411_at	PARD3B	par-3 partitioning defective 3 homolog B (C. elegans)	-1.38188
	Hs.711490	202730_s_at	MIR4680 /// PDCD4	microRNA 4680 /// programmed cell death 4 (neoplastic transformation inhibitor)	-1.38144
	Hs.6421	223457_at	COPG2	coatomer protein complex, subunit gamma 2	-1.38125
	Hs.665717	1564331_at	ZNF846	zinc finger protein 846	-1.38076
	Hs.646353	208106_x_at	PSG6	pregnancy specific beta-1-glycoprotein 6	-1.38011
	Hs.173030	223487_x_at	GNB4	guanine nucleotide binding protein (G protein), beta polypeptide 4	-1.37996
	Hs.125056	228032_s_at	DENND1B	DENN/MADD domain containing 1B	-1.37761
	Hs.380048	235562_at	C3orf70	chromosome 3 open reading frame 70	-1.37723
	Hs.657843	242033_at	RNF180	ring finger protein 180	-1.37385
	Hs.523744	1552617_a_at	RFWD2	ring finger and WD repeat domain 2, E3 ubiquitin protein ligase	-1.37384
	Hs.584833	216941_s_at	TAF1B	TATA box binding protein (TBP)-associated factor, RNA polymerase I, B, 63kDa	-1.37143
	Hs.486542	201101_s_at	BCLAF1	BCL2-associated transcription factor 1	-1.36964
	Hs.433442	203333_at	KIFAP3	kinesin-associated protein 3	-1.36946
	Hs.274329	209917_s_at	TP53TG1	TP53 target 1 (non-protein coding)	-1.36942
	Hs.118554	222714_s_at	LACTB2	lactamase, beta 2	-1.36937
	Hs.40582	211348_s_at	CDC14B	CDC14 cell division cycle 14 homolog B (S. cerevisiae)	-1.36899
	Hs.121396	1553726_s_at	C6orf170	chromosome 6 open reading frame 170	-1.36751
	Hs.607407	202719_s_at	TES	testis derived transcript (3 LIM domains)	-1.36731

	Hs.658514	219874_at	SLC12A8	solute carrier family 12 (potassium/chloride transporters), member 8	-1.36688
	Hs.131673	225700_at	GLCCI1	glucocorticoid induced transcript 1	-1.36575
	Hs.271876	205308_at	ZC2HC1A	zinc finger, C2HC-type containing 1A	-1.36558
	Hs.168762	204062_s_at	ULK2	unc-51-like kinase 2 (C. elegans)	-1.36556
	Hs.160211	222439_s_at	THRAP3	thyroid hormone receptor associated protein 3	-1.36446
	Hs.417022	204569_at	ICK	intestinal cell (MAK-like) kinase	-1.36402
	Hs.197644	218603_at	HECA	headcase homolog (Drosophila)	-1.36308
	Hs.231883	225106_s_at	OGFOD1	2-oxoglutarate and iron-dependent oxygenase domain containing 1	-1.36299
	Hs.655964	1569594_a_at	NEMF	nuclear export mediator factor	-1.36292
	Hs.656902	202956_at	ARFGEF1	ADP-ribosylation factor guanine nucleotide-exchange factor 1 (brefeldin A-inhibited)	-1.36291
	Hs.195403	219921_s_at	DOCK5	dedicator of cytokinesis 5	-1.36287
	Hs.25155	201829_at	NET1	neuroepithelial cell transforming 1	-1.35986
	Hs.702872	203242_s_at	PDLIM5	PDZ and LIM domain 5	-1.35934
	Hs.509008	223255_at	G2E3	G2/M-phase specific E3 ubiquitin protein ligase	-1.35896
	Hs.567367	225885_at	EEA1	early endosome antigen 1	-1.35856
	Hs.345588	226876_at	FAM101B	family with sequence similarity 101, member B	-1.35811
	Hs.482043	202784_s_at	NNT	nicotinamide nucleotide transhydrogenase	-1.35705
	Hs.500409	200946_x_at	GLUD1	glutamate dehydrogenase 1	-1.35523
	Hs.406096	217741_s_at	ZFAND5	zinc finger, AN1-type domain 5	-1.3552
	Hs.175473	202588_at	AK1	adenylate kinase 1	-1.35292
	Hs.485910	232902_s_at	RARS2	arginyl-tRNA synthetase 2, mitochondrial	-1.35226
	Hs.334772	233543_s_at	FAM175A	family with sequence similarity 175, member A	-1.35215
	Hs.49582	201602_s_at	PPP1R12A	protein phosphatase 1, regulatory subunit 12A	-1.34963
	Hs.710624	231118_at	ANKRD35	ankyrin repeat domain 35	-1.34831
	Hs.505202	214806_at	BICD1	bicaudal D homolog 1 (Drosophila)	-1.3481
	Hs.546268	219027_s_at	MYO9A	myosin IXA	-1.34783
	Hs.223296	1563111_a_at	PIGX	phosphatidylinositol glycan anchor biosynthesis, class X	-1.34746
	Hs.654857	219428_s_at	PXMP4	peroxisomal membrane protein 4, 24kDa	-1.34392
	Hs.592184	210849_s_at	VPS41	vacuolar protein sorting 41 homolog (S. cerevisiae)	-1.34364
	Hs.291000	235061_at	PPM1K	protein phosphatase, Mg2+/Mn2+ dependent, 1K	-1.34323
	Hs.706676	227905_s_at	AZI2	5-azacytidine induced 2	-1.34315
	Hs.643754	225452_at	MED1	mediator complex subunit 1	-1.34258
	Hs.435004	216392_s_at	SEC23IP	SEC23 interacting protein	-1.34049
	Hs.709689	218962_s_at	TMEM168	transmembrane protein 168	-1.34009
	Hs.535394	214825_at	FAM155A	family with sequence similarity 155, member A	-1.33919
	Hs.40510	1552774_a_at	SLC25A27	solute carrier family 25, member 27	-1.33916
	Hs.534189	212926_at	SMC5	structural maintenance of chromosomes 5	-1.33813
	---	213087_s_at	EEF1D	eukaryotic translation elongation factor 1 delta (guanine nucleotide exchange protein)	-1.33693
	Hs.543039	239466_at	LOC344595	uncharacterized LOC344595	-1.33693
	Hs.478407	229519_at	FXR1	fragile X mental retardation, autosomal homolog 1	-1.33688
	Hs.69855	222975_s_at	CSDE1	cold shock domain containing E1, RNA-binding	-1.33469
	Hs.653144	233191_at	RUFY2	RUN and FYVE domain containing 2	-1.33444
	Hs.708182	202583_s_at	RANBP9	RAN binding protein 9	-1.33287
	Hs.631618	209344_at	TPM4	tropomyosin 4	-1.33114
	Hs.306307	214578_s_at	ROCK1	Rho-associated, coiled-coil containing protein kinase 1	-1.32911
	Hs.9873	212162_at	KIDINS220	kinase D-interacting substrate, 220kDa	-1.32801
	Hs.80720	229114_at	GAB1	GRB2-associated binding protein 1	-1.32787
	Hs.315167	219000_s_at	DSCC1	defective in sister chromatid cohesion 1 homolog (S. cerevisiae)	-1.32672
	Hs.81874	204168_at	MGST2	microsomal glutathione S-transferase 2	-1.32588
	Hs.531111	214659_x_at	YLPM1	YLP motif containing 1	-1.32571
	Hs.724	1316_at	THRA	thyroid hormone receptor, alpha	-1.32548
	Hs.155827	204384_at	GOLGA2	golgin A2	-1.32385

Hs.369017	221960_s_at	RAB2A	RAB2A, member RAS oncogene family	-1.32363
Hs.523080	212423_at	ZCCHC24	zinc finger, CCHC domain containing 24	-1.32318
Hs.327252	226087_at	LZIC	leucine zipper and CTNNBIP1 domain containing	-1.32263
Hs.740395	217802_s_at	NUCKS1	nuclear casein kinase and cyclin-dependent kinase substrate 1	-1.32218
Hs.593995	216511_s_at	TCF7L2	transcription factor 7-like 2 (T-cell specific, HMG-box)	-1.32201
Hs.8739	235623_at	ELP2	elongation protein 2 homolog (S. cerevisiae)	-1.31959
Hs.334868	228070_at	PPP2R5E	protein phosphatase 2, regulatory subunit B', epsilon isoform	-1.31921
Hs.740373	212113_at	ATXN7L3B	ataxin 7-like 3B	-1.31917
Hs.66708	201337_s_at	VAMP3	vesicle-associated membrane protein 3 (cellubrevin)	-1.31841
Hs.632272	235057_at	ITCH	itchy E3 ubiquitin protein ligase	-1.31824
Hs.461787	214075_at	NENF	neudesin neurotrophic factor	-1.31777
Hs.368982	208050_s_at	CASP2	caspase 2, apoptosis-related cysteine peptidase	-1.31594
Hs.21145	208503_s_at	GATAD1	GATA zinc finger domain containing 1	-1.31545
Hs.644000	201768_s_at	CLINT1	clathrin interactor 1	-1.31481
Hs.656902	202955_s_at	ARFGEF1	ADP-ribosylation factor guanine nucleotide-exchange factor 1 (brefeldin A-inhibited)	-1.31479
Hs.448979	206770_s_at	SLC35A3	solute carrier family 35 (UDP-N-acetylglucosamine (UDP-GlcNAc) transporter), member A3	-1.3142
Hs.634120	238574_at	SLC25A51	solute carrier family 25, member 51	-1.31376
Hs.370147	220072_at	CSPP1	centrosome and spindle pole associated protein 1	-1.31305
Hs.340623	235628_x_at	FLJ33630	uncharacterized LOC644873	-1.31086
Hs.272939	1557167_at	HCG11	HLA complex group 11 (non-protein coding)	-1.30993
Hs.647120	222414_at	MLL3	myeloid/lymphoid or mixed-lineage leukemia 3	-1.30864
Hs.436687	215780_s_at	SET /// SETP4	SET nuclear oncogene /// SET pseudogene 4	-1.30858
Hs.605153	203753_at	TCF4	transcription factor 4	-1.30314
Hs.708182	216125_s_at	RANBP9	RAN binding protein 9	-1.30243
Hs.466391	211563_s_at	URI1	URI1, prefoldin-like chaperone	-1.30105
Hs.654740	214820_at	BRWD1	bromodomain and WD repeat domain containing 1	-1.30095
Hs.733180	228606_at	TCTEX1D2	Tctex1 domain containing 2	-1.30035
Hs.731996	228027_at	ARMCX5-GPRASP2 /// GPRASP2	ARMCX5-GPRASP2 readthrough /// G protein-coupled receptor associated sorting protein 2	-1.3003
Hs.183713	204464_s_at	EDNRA	endothelin receptor type A	-1.30009
Hs.458412	1568594_s_at	TRIM52	tripartite motif containing 52	-1.29936
Hs.533499	231716_at	RC3H2	ring finger and CCCH-type domains 2	-1.29856
Hs.157078	221826_at	ANGEL2	angel homolog 2 (Drosophila)	-1.29848
Hs.97439	209155_s_at	NT5C2	5'-nucleotidase, cytosolic II	-1.29823
Hs.24678	221268_s_at	SGPP1	sphingosine-1-phosphate phosphatase 1	-1.29819
Hs.740395	224581_s_at	NUCKS1	nuclear casein kinase and cyclin-dependent kinase substrate 1	-1.29785
Hs.102267	204298_s_at	LOX	lysyl oxidase	-1.29746
Hs.540550	229586_at	CHD9	chromodomain helicase DNA binding protein 9	-1.29675
Hs.61188	227678_at	XRCC6BP1	XRCC6 binding protein 1	-1.29657
Hs.435004	209175_at	SEC23IP	SEC23 interacting protein	-1.29598
---	236657_at	LOC100288911	uncharacterized LOC100288911	-1.29334
Hs.327527	212520_s_at	SMARCA4	SWI/SNF related, matrix associated, actin dependent regulator of chromatin, subfamily a	-1.29241
Hs.202238	243927_x_at	KIAA1429	KIAA1429	-1.29087
Hs.428360	220159_at	ABCA11P	ATP-binding cassette, sub-family A (ABC1), member 11, pseudogene	-1.29063
Hs.314246	211009_s_at	ZNF271	zinc finger protein 271	-1.28915
Hs.418198	225761_at	PAPD4	PAP associated domain containing 4	-1.289
Hs.553131	1558685_a_at	LOC158960	uncharacterized protein BC009467	-1.28851
Hs.485784	202319_at	SENP6	SUMO1/sentrin specific peptidase 6	-1.28843
Hs.654567	214787_at	DENND4A	DENN/MADD domain containing 4A	-1.28792
Hs.464137	209600_s_at	ACOX1	acyl-CoA oxidase 1, palmitoyl	-1.28684
Hs.87889	206061_s_at	DICER1	dicer 1, ribonuclease type III	-1.28642
Hs.406787	229955_at	FBXO3	F-box protein 3	-1.28637

Hs.64016	207808_s_at	PROS1	protein S (alpha)	-1.28633
Hs.272927	204344_s_at	SEC23A	Sec23 homolog A (S. cerevisiae)	-1.28585
Hs.654560	228904_at	HOXB3	homeobox B3	-1.2851
Hs.515487	1563431_x_at	CALM3	Calmodulin 3 (phosphorylase kinase, delta)	-1.28489
Hs.528574	225802_at	TOP1MT	topoisomerase (DNA) I, mitochondrial	-1.28451
Hs.655519	225894_at	SYNPO2	synaptopodin 2	-1.28436
Hs.631954	226826_at	LSM11	LSM11, U7 small nuclear RNA associated	-1.2828
Hs.40758	229072_at	RAB30	RAB30, member RAS oncogene family	-1.28177
Hs.591692	228536_at	PRMT10	protein arginine methyltransferase 10 (putative)	-1.28141
---	205158_at	RNASE4	ribonuclease, RNase A family, 4	-1.28086
Hs.497183	201363_s_at	IVNS1ABP	influenza virus NS1A binding protein	-1.28004
Hs.165762	228220_at	FCHO2	FCH domain only 2	-1.27978
Hs.655964	1557950_at	NEMF	nuclear export mediator factor	-1.27971
Hs.184720	214221_at	ALMS1	Alstrom syndrome 1	-1.27786
Hs.570189	225908_at	IAH1	isoamyl acetate-hydrolyzing esterase 1 homolog (S. cerevisiae)	-1.27755
Hs.476415	218158_s_at	APPL1	adaptor protein, phosphotyrosine interaction, PH domain and leucine zipper containing 1	-1.27701
Hs.85195	204784_s_at	MLF1	myeloid leukemia factor 1	-1.27671
Hs.30977	227041_at	SESTD1	SEC14 and spectrin domains 1	-1.27588
Hs.445511	202131_s_at	RIOK3	RIO kinase 3 (yeast)	-1.2757
Hs.497183	206245_s_at	IVNS1ABP	influenza virus NS1A binding protein	-1.27536
Hs.12102	208781_x_at	SNX3	sorting nexin 3	-1.27458
Hs.409582	226070_at	C9orf142	chromosome 9 open reading frame 142	-1.27381
---	239010_at	LOC100653149	uncharacterized LOC100653149	-1.27342
Hs.156928	1556301_at	LOC100287015	Uncharacterized LOC100287015	-1.27316
Hs.591360	225010_at	CCDC6	coiled-coil domain containing 6	-1.27266
Hs.122927	226425_at	CLIP4	CAP-GLY domain containing linker protein family, member 4	-1.27264
Hs.711490	212593_s_at	MIR4680 /// PDCD4	microRNA 4680 /// programmed cell death 4 (neoplastic transformation inhibitor)	-1.27227
Hs.368639	226885_at	RNF217	ring finger protein 217	-1.27177
Hs.466436	219818_s_at	GPATCH1	G patch domain containing 1	-1.27081
Hs.195060	1555803_a_at	C11orf57	chromosome 11 open reading frame 57	-1.27075
Hs.302977	218374_s_at	C12orf4	chromosome 12 open reading frame 4	-1.26952
Hs.20000	226416_at	ERI1	exoribonuclease 1	-1.26894
Hs.310645	207791_s_at	RAB1A	RAB1A, member RAS oncogene family	-1.26788
Hs.146551	37170_at	BMP2K	BMP2 inducible kinase	-1.26781
Hs.149387	203216_s_at	MYO6	myosin VI	-1.2671
Hs.643436	1554433_a_at	ZNF146	zinc finger protein 146	-1.26565
Hs.372309	222617_s_at	FAM204A	family with sequence similarity 204, member A	-1.26477
Hs.654580	1554885_a_at	LOC100653079 /// PRIM2	uncharacterized LOC100653079 /// primase, DNA, polypeptide 2 (58kDa)	-1.26473
Hs.475018	212931_at	TCF20	transcription factor 20 (AR1)	-1.26391
Hs.180408	214140_at	SLC25A16	solute carrier family 25 (mitochondrial carrier; Graves disease autoantigen), member 16	-1.26342
Hs.438953	228584_at	SGCB	sarcoglycan, beta (43kDa dystrophin-associated glycoprotein)	-1.26268
Hs.530000	208310_s_at	CCZ1 /// CCZ1B	CCZ1 vacuolar protein trafficking and biogenesis associated homolog (S. cerevisiae) ///	-1.2626
Hs.546479	235142_at	ZBTB8A	zinc finger and BTB domain containing 8A	-1.26203
Hs.59719	210788_s_at	DHRS7	dehydrogenase/reductase (SDR family) member 7	-1.2618
Hs.643813	1553530_a_at	ITGB1	integrin, beta 1 (fibronectin receptor, beta polypeptide, antigen CD29 includes MDF2, M	-1.26134
Hs.379972	238783_at	TMEM161B	transmembrane protein 161B	-1.25984
Hs.101302	231766_s_at	COL12A1	collagen, type XII, alpha 1	-1.25983
Hs.433484	235272_at	SBSN	suprabasin	-1.25929
Hs.288304	1553749_at	FAM76B	family with sequence similarity 76, member B	-1.25721
Hs.22587	203017_s_at	SSX2IP	synovial sarcoma, X breakpoint 2 interacting protein	-1.25612
Hs.233325	211330_s_at	HFE	hemochromatosis	-1.2552

	Hs.131180	219953_s_at	AKIP1	A kinase (PRKA) interacting protein 1	-1.25515
	Hs.515210	200664_s_at	DNAJB1	DnaJ (Hsp40) homolog, subfamily B, member 1	-1.25508
	Hs.479867	204739_at	CENPC1	centromere protein C 1	-1.25485
	Hs.418198	1556277_a_at	PAPD4	PAP associated domain containing 4	-1.25446
	Hs.415342	213311_s_at	TCF25	transcription factor 25 (basic helix-loop-helix)	-1.25433
	Hs.603118	201901_s_at	YY1	YY1 transcription factor	-1.25368
	---	225199_at	LOC100505487	uncharacterized LOC100505487	-1.25346
	Hs.521640	201222_s_at	RAD23B	RAD23 homolog B (S. cerevisiae)	-1.25339
	Hs.188456	216593_s_at	LOC100505991 /// PIGC	uncharacterized LOC100505991 /// phosphatidylinositol glycan anchor biosynthesis, class	-1.25289
	Hs.509451	222230_s_at	ACTR10	actin-related protein 10 homolog (S. cerevisiae)	-1.25259
	Hs.700632	228801_at	ORMDL1	ORM1-like 1 (S. cerevisiae)	-1.25244
	Hs.646386	1558755_x_at	ZNF763	zinc finger protein 763	-1.25187
	Hs.368264	1554365_a_at	PPP2R5C	protein phosphatase 2, regulatory subunit B', gamma	-1.2495
	Hs.729113	1558699_a_at	HERPUD2	HERPUD family member 2	-1.24932
	Hs.591530	1553034_at	SDCCAG8	serologically defined colon cancer antigen 8	-1.24871
	Hs.349150	227718_at	PURB	purine-rich element binding protein B	-1.24791
	Hs.643553	226235_at	LOC339290	uncharacterized LOC339290	-1.24779
	Hs.656208	212885_at	MPHOSPH10	M-phase phosphoprotein 10 (U3 small nucleolar ribonucleoprotein)	-1.24705
	Hs.655182	235425_at	SGOL2	shugoshin-like 2 (S. pombe)	-1.24699
	Hs.709010	239035_at	MTHFR	methylenetetrahydrofolate reductase (NAD(P)H)	-1.24637
	Hs.512181	213289_at	APOOL	apolipoprotein O-like	-1.2458
	Hs.306083	225794_s_at	C22orf32	chromosome 22 open reading frame 32	-1.24565
	Hs.180402	219849_at	ZNF671	zinc finger protein 671	-1.24429
	Hs.524161	230490_x_at	RSU1	Ras suppressor protein 1	-1.24416
	Hs.648394	211937_at	EIF4B	eukaryotic translation initiation factor 4B	-1.24404
	Hs.115467	240344_x_at	LYRM7	Lyrm7 homolog (mouse)	-1.24395
	Hs.713564	218134_s_at	RBM22	RNA binding motif protein 22	-1.24374
	Hs.433702	208705_s_at	EIF5	eukaryotic translation initiation factor 5	-1.24345
	Hs.437338	217286_s_at	NDRG3	NDRG family member 3	-1.24258
	Hs.528731	236562_at	ZNF439	zinc finger protein 439	-1.24143
	Hs.369284	218859_s_at	ESF1	ESF1, nucleolar pre-rRNA processing protein, homolog (S. cerevisiae)	-1.2411
	Hs.443661	219156_at	SYNJ2BP /// SYNJ2BP-COX16	synaptojanin 2 binding protein /// SYNJ2BP-COX16 readthrough	-1.24107
	Hs.255932	223002_s_at	XRN2	5'-3' exoribonuclease 2	-1.24068
	Hs.263671	212397_at	RDX	radixin	-1.24052
	Hs.35199	237706_at	STXBP4	syntaxin binding protein 4	-1.24034
	Hs.221941	217889_s_at	CYBRD1	cytochrome b reductase 1	-1.24032
	Hs.38114	235253_at	RAD1	RAD1 homolog (S. pombe)	-1.23996
	Hs.642618	218450_at	HEBP1	heme binding protein 1	-1.23956
	Hs.162852	235114_x_at	HOOK3	hook homolog 3 (Drosophila)	-1.23892
	Hs.656803	243552_at	MBTD1	mbt domain containing 1	-1.23881
	Hs.531713	202143_s_at	COPS8	COP9 constitutive photomorphogenic homolog subunit 8 (Arabidopsis)	-1.23808
	Hs.732391	222837_s_at	NAA15	N(alpha)-acetyltransferase 15, NatA auxiliary subunit	-1.23751
	Hs.631814	228196_s_at	LARP4B	La ribonucleoprotein domain family, member 4B	-1.23638
	Hs.158688	201024_x_at	EIF5B	eukaryotic translation initiation factor 5B	-1.23583
	Hs.101014	203492_x_at	CEP57	centrosomal protein 57kDa	-1.23517
	Hs.418533	201456_s_at	BUB3	budding uninhibited by benzimidazoles 3 homolog (yeast)	-1.23498
	Hs.448979	226894_at	SLC35A3	solute carrier family 35 (UDP-N-acetylglucosamine (UDP-GlcNAc) transporter), member A3	-1.23475
	Hs.334637	226879_at	HVCN1	hydrogen voltage-gated channel 1	-1.2346
	Hs.379548	234982_at	UBR3	ubiquitin protein ligase E3 component n-recognin 3 (putative)	-1.23408
	Hs.464184	202083_s_at	SEC14L1	SEC14-like 1 (S. cerevisiae)	-1.23402
	Hs.102696	230235_at	MCTS1	malignant T cell amplified sequence 1	-1.23388

	Hs.655378	227249_at	NDE1	NudE nuclear distribution E homolog 1 (A. nidulans)	-1.23354
	Hs.591122	226873_at	FAM63B	family with sequence similarity 63, member B	-1.2327
	Hs.632486	200798_x_at	MCL1	myeloid cell leukemia sequence 1 (BCL2-related)	-1.23247
	Hs.407015	242138_at	DLX1	distal-less homeobox 1	-1.23223
	Hs.135763	234140_s_at	STIM2	stromal interaction molecule 2	-1.23176
	Hs.125056	1557309_at	DENND1B	DENN/MADD domain containing 1B	-1.22949
	Hs.327736	201991_s_at	KIF5B	kinesin family member 5B	-1.22942
	Hs.619530	231437_at	SLC35D2	solute carrier family 35, member D2	-1.22853
	Hs.465433	227049_at	ZADH2	zinc binding alcohol dehydrogenase domain containing 2	-1.22849
	Hs.170622	1555730_a_at	CFL1	cofilin 1 (non-muscle)	-1.22835
	Hs.577202	202453_s_at	GTF2H1	general transcription factor IIH, polypeptide 1, 62kDa	-1.22827
	---	214850_at	LOC100170939	glucuronidase, beta pseudogene	-1.22795
	Hs.432424	203374_s_at	TPP2	tripeptidyl peptidase II	-1.2272
	Hs.534641	213315_x_at	CXorf40A	chromosome X open reading frame 40A	-1.22713
	Hs.24485	209259_s_at	SMC3	structural maintenance of chromosomes 3	-1.22673
	Hs.66708	211749_s_at	VAMP3	vesicle-associated membrane protein 3 (cellubrevin)	-1.2263
	Hs.309316	201138_s_at	SSB	Sjogren syndrome antigen B (autoantigen La)	-1.22625
	Hs.478465	221713_s_at	MAP6D1	MAP6 domain containing 1	-1.22552
	Hs.493808	207839_s_at	TMEM8B	transmembrane protein 8B	-1.22548
	Hs.153026	209306_s_at	SWAP70	SWAP switching B-cell complex 70kDa subunit	-1.22529
	Hs.522672	1554098_at	SPIN3	spindlin family, member 3	-1.22474
	Hs.233325	206087_x_at	HFE	hemochromatosis	-1.22457
	Hs.591122	222111_at	FAM63B	family with sequence similarity 63, member B	-1.22387
	Hs.388220	233647_s_at	CDADC1	cytidine and dCMP deaminase domain containing 1	-1.2233
	Hs.221436	238447_at	RBMS3	RNA binding motif, single stranded interacting protein 3	-1.22263
	Hs.285197	1558254_s_at	SRPK2	SRSF protein kinase 2	-1.22239
	Hs.271749	204448_s_at	PDCL	phosducin-like	-1.22201
	Hs.279257	208857_s_at	PCMT1	protein-L-isoaspartate (D-aspartate) O-methyltransferase	-1.2216
	Hs.88778	209213_at	CBR1	carbonyl reductase 1	-1.22143
	Hs.740404	225707_at	ARL6IP6	ADP-ribosylation-like factor 6 interacting protein 6	-1.22014
	Hs.374127	209658_at	CDC16	cell division cycle 16 homolog (S. cerevisiae)	-1.22009
	Hs.36859	227693_at	WDR20	WD repeat domain 20	-1.21976
	Hs.728857	200728_at	ACTR2	ARP2 actin-related protein 2 homolog (yeast)	-1.21943
	Hs.15154	204955_at	SRPX	sushi-repeat containing protein, X-linked	-1.21906
	Hs.49774	1555579_s_at	PTPRM	protein tyrosine phosphatase, receptor type, M	-1.21721
	Hs.581438	200806_s_at	HSPD1	heat shock 60kDa protein 1 (chaperonin)	-1.2171
	Hs.472024	220477_s_at	TMEM230	transmembrane protein 230	-1.21634
	Hs.459106	212461_at	AZIN1	antizyme inhibitor 1	-1.21625
	Hs.621695	224567_x_at	LOC100507645 /// MALAT1	uncharacterized LOC100507645 /// metastasis associated lung adenocarcinoma transcript 1	-1.21591
	Hs.460988	202370_s_at	CBFB	core-binding factor, beta subunit	-1.21585
	Hs.510324	212265_at	QKI	QKI, KH domain containing, RNA binding	-1.21536
	Hs.369017	208734_x_at	RAB2A	RAB2A, member RAS oncogene family	-1.21523
	Hs.591061	228927_at	ZNF397	zinc finger protein 397	-1.21502
	Hs.507475	208021_s_at	RFC1	replication factor C (activator 1) 1, 145kDa	-1.21491
	Hs.306327	213530_at	RAB3GAP1	RAB3 GTPase activating protein subunit 1 (catalytic)	-1.21451
	Hs.575782	214943_s_at	ARID4B /// RBM34	AT rich interactive domain 4B (RBP1-like) /// RNA binding motif protein 34	-1.21446
	Hs.198308	202749_at	WRB	tryptophan rich basic protein	-1.21355
	Hs.148670	212651_at	RHOBTB1	Rho-related BTB domain containing 1	-1.21305
	Hs.436585	207416_s_at	NFATC3	nuclear factor of activated T-cells, cytoplasmic, calcineurin-dependent 3	-1.21285
	Hs.433668	219600_s_at	TMEM50B	transmembrane protein 50B	-1.21154
	Hs.444229	223422_s_at	ARHGAP24	Rho GTPase activating protein 24	-1.2112
	Hs.126221	209412_at	TRAPPC10	trafficking protein particle complex 10	-1.21115

	Hs.440219	209088_s_at	UBN1	ubinuclein 1	-1.21105
	Hs.437256	212243_at	GCOM1 /// POLR2M	GRINL1A complex locus 1 /// polymerase (RNA) II (DNA directed) polypeptide M	-1.20877
	Hs.127675	229958_at	CLN8	ceroid-lipofuscinosis, neuronal 8 (epilepsy, progressive with mental retardation)	-1.2084
	Hs.233552	207318_s_at	CDK13	cyclin-dependent kinase 13	-1.20805
	Hs.477009	212381_at	USP24	ubiquitin specific peptidase 24	-1.2073
	Hs.620764	226010_at	SLC25A23	solute carrier family 25 (mitochondrial carrier; phosphate carrier), member 23	-1.20716
	Hs.523744	234950_s_at	RFWD2	ring finger and WD repeat domain 2, E3 ubiquitin protein ligase	-1.20599
	Hs.74615	203131_at	PDGFRA	platelet-derived growth factor receptor, alpha polypeptide	-1.20563
	Hs.431850	224621_at	MAPK1	mitogen-activated protein kinase 1	-1.20557
	Hs.49582	201603_at	PPP1R12A	protein phosphatase 1, regulatory subunit 12A	-1.2051
	Hs.339024	225790_at	MSRB3	methionine sulfoxide reductase B3	-1.20504
	Hs.432760	201950_x_at	CAPZB	capping protein (actin filament) muscle Z-line, beta	-1.20473
	Hs.189409	230389_at	FNBP1	formin binding protein 1	-1.20456
	Hs.516978	225224_at	C20orf112	chromosome 20 open reading frame 112	-1.20345
	Hs.437894	222737_s_at	BRD7	bromodomain containing 7	-1.20326
	Hs.460232	206555_s_at	THUMPD1	THUMP domain containing 1	-1.20278
	Hs.736055	1560402_at	GAS5	growth arrest-specific 5 (non-protein coding)	-1.20213
	Hs.408142	212779_at	KIAA1109	KIAA1109	-1.20178
	Hs.303787	205084_at	BCAP29	B-cell receptor-associated protein 29	-1.20175
	Hs.525549	224945_at	BTBD7	BTB (POZ) domain containing 7	-1.20164
	Hs.306307	213044_at	ROCK1	Rho-associated, coiled-coil containing protein kinase 1	-1.20145
	Hs.436031	214666_x_at	IREB2	iron-responsive element binding protein 2	-1.20115
	Hs.489040	208920_at	SRI	sorcin	-1.20091
	Hs.173135	202969_at	DYRK2	dual-specificity tyrosine-(Y)-phosphorylation regulated kinase 2	-1.20049
	Hs.655552	221039_s_at	ASAP1	ArfGAP with SH3 domain, ankyrin repeat and PH domain 1	1.20001
	Hs.143250	201645_at	TNC	tenascin C	1.20089
	Hs.694798	213199_at	C2CD3	C2 calcium-dependent domain containing 3	1.20142
	Hs.177841	221530_s_at	BHLHE41	basic helix-loop-helix family, member e41	1.20189
	Hs.513315	224477_s_at	NUDT16L1	nudix (nucleoside diphosphate linked moiety X)-type motif 16-like 1	1.20371
	Hs.213198	222612_at	PSPC1	paraspeckle component 1	1.20377
	Hs.413901	212871_at	MAPKAPK5	mitogen-activated protein kinase-activated protein kinase 5	1.20395
	---	217542_at	MDM2	Mdm2, p53 E3 ubiquitin protein ligase homolog (mouse)	1.20474
	Hs.183817	227256_at	USP31	ubiquitin specific peptidase 31	1.20486
	Hs.526933	225148_at	RPS19BP1	ribosomal protein S19 binding protein 1	1.20559
	Hs.523438	219405_at	TRIM68	tripartite motif containing 68	1.20697
	Hs.356769	209166_s_at	MAN2B1	mannosidase, alpha, class 2B, member 1	1.20749
	Hs.512465	222977_at	SURF4	surfeit 4	1.20913
	Hs.443031	201576_s_at	GLB1	galactosidase, beta 1	1.21207
	Hs.454534	202152_x_at	USF2	upstream transcription factor 2, c-fos interacting	1.2127
	Hs.158195	209657_s_at	HSF2	heat shock transcription factor 2	1.2131
	Hs.42806	224472_x_at	SDF4	stromal cell derived factor 4	1.2132
	Hs.248785	225440_at	AGPAT3	1-acylglycerol-3-phosphate O-acyltransferase 3	1.21419
	Hs.5710	201200_at	CREG1	cellular repressor of E1A-stimulated genes 1	1.21477
	Hs.728802	201748_s_at	SAFB	scaffold attachment factor B	1.21534
	Hs.436585	210555_s_at	NFATC3	nuclear factor of activated T-cells, cytoplasmic, calcineurin-dependent 3	1.21657
	Hs.374477	210011_s_at	EWSR1	Ewing sarcoma breakpoint region 1	1.21709
	Hs.585209	229558_at	C16orf88	chromosome 16 open reading frame 88	1.2178
	Hs.93659	208658_at	PDIA4	protein disulfide isomerase family A, member 4	1.21789
	Hs.500842	223494_at	MGEA5	meningioma expressed antigen 5 (hyaluronidase)	1.21806
	Hs.5443	230427_s_at	BAG5	BCL2-associated athanogene 5	1.21857
	Hs.489190	203775_at	SLC25A13	solute carrier family 25 (aspartate/glutamate carrier), member 13	1.21969
	Hs.414795	202628_s_at	SERPINE1	serpin peptidase inhibitor, clade E (nexin, plasminogen activator inhibitor type 1), me	1.21986

	Hs.633762	229852_at	NMNAT1	nicotinamide nucleotide adenylyltransferase 1	1.21986
	Hs.509140	217986_s_at	BAZ1A	bromodomain adjacent to zinc finger domain, 1A	1.22105
	Hs.593344	225037_at	SLC35C2	solute carrier family 35, member C2	1.22222
	Hs.455109	53076_at	B4GALT7	xylosylprotein beta 1,4-galactosyltransferase, polypeptide 7 (galactosyltransferase I)	1.22396
	Hs.461954	235677_at	SRR	Serine racemase	1.22428
	Hs.514997	212540_at	CDC34	cell division cycle 34 homolog (S. cerevisiae)	1.2246
	Hs.516632	221782_at	DNAJC10	DnaJ (Hsp40) homolog, subfamily C, member 10	1.22646
	Hs.438830	218435_at	DNAJC15	DnaJ (Hsp40) homolog, subfamily C, member 15	1.22648
	Hs.449098	202792_s_at	PPP6R2	protein phosphatase 6, regulatory subunit 2	1.23097
	Hs.474833	202332_at	CSNK1E	casein kinase 1, epsilon	1.23112
	Hs.507971	226795_at	LRCH1	leucine-rich repeats and calponin homology (CH) domain containing 1	1.23133
	Hs.265018	226475_at	FAM118A	family with sequence similarity 118, member A	1.23148
	Hs.68257	202355_s_at	GTF2F1	general transcription factor IIF, polypeptide 1, 74kDa	1.23217
	Hs.334587	207836_s_at	RBPMS	RNA binding protein with multiple splicing	1.23238
	Hs.234572	226738_at	WDR81	WD repeat domain 81	1.23299
	Hs.24545	50376_at	ZNF444	zinc finger protein 444	1.23316
	Hs.659809	214995_s_at	APOBEC3F /// APOBEC3G	apolipoprotein B mRNA editing enzyme, catalytic polypeptide-like 3F /// apolipoprotein	1.23411
	Hs.93659	211048_s_at	PDIA4	protein disulfide isomerase family A, member 4	1.23432
	Hs.522099	209998_at	PIGO	phosphatidylinositol glycan anchor biosynthesis, class O	1.23496
	Hs.226390	209773_s_at	RRM2	ribonucleotide reductase M2	1.23537
	Hs.567619	226384_at	PPAPDC1B	phosphatidic acid phosphatase type 2 domain containing 1B	1.2358
	Hs.654665	212194_s_at	TM9SF4	transmembrane 9 superfamily protein member 4	1.23661
	Hs.464071	201118_at	PGD	phosphogluconate dehydrogenase	1.23709
	Hs.485449	221050_s_at	GTPBP2	GTP binding protein 2	1.23754
	Hs.740380	201170_s_at	BHLHE40	basic helix-loop-helix family, member e40	1.23757
	Hs.48353	229101_at	IL17RA	interleukin 17 receptor A	1.23965
	Hs.558536	218860_at	NOC4L	nucleolar complex associated 4 homolog (S. cerevisiae)	1.23984
	Hs.656778	238542_at	ULBP2	UL16 binding protein 2	1.24138
	Hs.523710	212860_at	ZDHHC18	zinc finger, DHHC-type containing 18	1.24193
	Hs.516707	221984_s_at	FAM134A	family with sequence similarity 134, member A	1.24193
	Hs.524081	209509_s_at	DPAGT1	dolichyl-phosphate (UDP-N-acetylglucosamine) N-acetylglucosaminephosphotransferase 1 (G	1.24387
	Hs.368611	229336_at	ST3GAL2	ST3 beta-galactoside alpha-2,3-sialyltransferase 2	1.24634
	Hs.191539	225022_at	GOPC	golgi-associated PDZ and coiled-coil motif containing	1.24646
	Hs.408324	217940_s_at	CARKD	carbohydrate kinase domain containing	1.24763
	Hs.521124	219819_s_at	MRPS28	mitochondrial ribosomal protein S28	1.24769
	Hs.643951	200913_at	PPM1G	protein phosphatase, Mg2+/Mn2+ dependent, 1G	1.2479
	Hs.632182	220947_s_at	TBC1D10B	TBC1 domain family, member 10B	1.2486
	Hs.456578	222001_x_at	LOC728855 /// LOC728875	uncharacterized LOC728855 /// uncharacterized LOC728875	1.25044
	Hs.355753	229577_at	AGPAT6	1-acylglycerol-3-phosphate O-acyltransferase 6 (lysophosphatidic acid acyltransferase,	1.25054
	Hs.487498	226640_at	DAGLB	diacylglycerol lipase, beta	1.25063
	Hs.420024	225192_at	CACUL1	CDK2-associated, cullin domain 1	1.2519
	Hs.517717	203408_s_at	SATB1	SATB homeobox 1	1.25235
	Hs.290758	208619_at	DDB1	damage-specific DNA binding protein 1, 127kDa	1.25414
	Hs.474095	1558139_at	FLJ39632 /// LOC100506303 /// LOC100653149 /// LOC400879	uncharacterized LOC642477 /// uncharacterized LOC100506303 /// uncharacterized LOC10065	1.25416
	Hs.148767	213179_at	RQCD1	RCD1 required for cell differentiation1 homolog (S. pombe)	1.25421
	Hs.279413	203422_at	POLD1	polymerase (DNA directed), delta 1, catalytic subunit	1.25469
	Hs.130098	40465_at	DDX23	DEAD (Asp-Glu-Ala-Asp) box polypeptide 23	1.25485
	Hs.440092	239067_s_at	PANX2	pannexin 2	1.25526
	Hs.714189	219491_at	LRFN4	leucine rich repeat and fibronectin type III domain containing 4	1.25718

	Hs.654965	201175_at	TMX2	thioredoxin-related transmembrane protein 2	1.26135
	Hs.534497	227366_at	RILP	Rab interacting lysosomal protein	1.26143
	Hs.409226	211342_x_at	MED12	mediator complex subunit 12	1.2635
	Hs.731427	213112_s_at	SQSTM1	sequestosome 1	1.26578
	Hs.459858	218971_s_at	WDR91	WD repeat domain 91	1.26609
	Hs.104672	1554966_a_at	FILIP1L	filamin A interacting protein 1-like	1.26697
	Hs.288215	224603_at	LOC100507246	uncharacterized LOC100507246	1.26793
	Hs.642946	238753_at	NCS1	neuronal calcium sensor 1	1.26834
	Hs.531249	229198_at	USP35	ubiquitin specific peptidase 35	1.26917
	Hs.593645	213942_at	MEGF6	multiple EGF-like-domains 6	1.26961
	Hs.404089	222749_at	SUFU	suppressor of fused homolog (Drosophila)	1.27001
	Hs.501857	212561_at	DENND5A	DENN/MADD domain containing 5A	1.27015
	Hs.42806	232032_x_at	SDF4	stromal cell derived factor 4	1.27138
	Hs.464829	203441_s_at	CDH2	cadherin 2, type 1, N-cadherin (neuronal)	1.27219
	Hs.515154	223419_at	FBXW9	F-box and WD repeat domain containing 9	1.27274
	Hs.437966	228225_at	PEX2	peroxisomal biogenesis factor 2	1.27305
	Hs.134742	226722_at	FAM20C	family with sequence similarity 20, member C	1.27318
	Hs.725212	220949_s_at	C7orf49 /// LOC653739	chromosome 7 open reading frame 49 /// uncharacterized LOC653739	1.2736
	Hs.516855	212437_at	CENPB	centromere protein B, 80kDa	1.27457
	Hs.567488	219920_s_at	AMIGO3 /// GMPPB	adhesion molecule with Ig-like domain 3 /// GDP-mannose pyrophosphorylase B	1.27476
	Hs.523822	235511_at	RBM4	RNA binding motif protein 4	1.27499
	Hs.38032	218673_s_at	ATG7	autophagy related 7	1.27676
	Hs.19192	204252_at	CDK2	cyclin-dependent kinase 2	1.27679
	Hs.187763	202102_s_at	BRD4	bromodomain containing 4	1.27698
	Hs.508848	227110_at	HNRNPC /// LOC100652761 /// LOC100653343	heterogeneous nuclear ribonucleoprotein C (C1/C2) /// uncharacterized LOC100652761 ///	1.27852
	Hs.516370	204065_at	CHST10	carbohydrate sulfotransferase 10	1.27913
	Hs.519347	238781_at	SREK1	splicing regulatory glutamine/lysine-rich protein 1	1.27914
	Hs.119177	200734_s_at	ARF3	ADP-ribosylation factor 3	1.28107
	Hs.387755	218561_s_at	LYRM4	LYR motif containing 4	1.28374
	Hs.655396	208776_at	PSMD11	proteasome (prosome, macropain) 26S subunit, non-ATPase, 11	1.28468
	Hs.501296	205865_at	ARID3A	AT rich interactive domain 3A (BRIGHT-like)	1.28514
	Hs.5452	203965_at	USP20	ubiquitin specific peptidase 20	1.28563
	Hs.709317	218601_at	URGCP	upregulator of cell proliferation	1.291
	Hs.715623	217584_at	NPC1	Niemann-Pick disease, type C1	1.29125
	Hs.511138	223772_s_at	TMEM87A	transmembrane protein 87A	1.29317
	Hs.437008	202894_at	EPHB4	EPH receptor B4	1.29333
	Hs.436568	209619_at	CD74	CD74 molecule, major histocompatibility complex, class II invariant chain	1.29383
	Hs.514297	217750_s_at	UBE2Z	ubiquitin-conjugating enzyme E2Z	1.29727
	Hs.533139	220659_s_at	C7orf43	chromosome 7 open reading frame 43	1.29757
	Hs.58351	204719_at	ABCA8	ATP-binding cassette, sub-family A (ABC1), member 8	1.29858
	Hs.193268	231984_at	MTAP	methylthioadenosine phosphorylase	1.29988
	Hs.532786	212708_at	MSL1	male-specific lethal 1 homolog (Drosophila)	1.30009
	Hs.268726	213308_at	SHANK2	SH3 and multiple ankyrin repeat domains 2	1.30068
	Hs.511801	224574_at	C17orf49 /// RNASEK-C17ORF49	chromosome 17 open reading frame 49 /// RNASEK-C17orf49 readthrough	1.30219
	Hs.533712	239635_at	RBM14	RNA binding motif protein 14	1.30468
	Hs.118666	212541_at	FLAD1	FAD1 flavin adenine dinucleotide synthetase homolog (S. cerevisiae)	1.30479
	Hs.460468	211982_x_at	XPO6	exportin 6	1.30522
	Hs.188553	1552329_at	RBBP6	retinoblastoma binding protein 6	1.30622
	Hs.174050	209059_s_at	EDF1	endothelial differentiation-related factor 1	1.30658

	Hs.524183	200895_s_at	FKBP4	FK506 binding protein 4, 59kDa	1.30669
	Hs.602085	225842_at	PHLDA1	pleckstrin homology-like domain, family A, member 1	1.30746
	Hs.513632	212228_s_at	COQ9	coenzyme Q9 homolog (S. cerevisiae)	1.30829
	Hs.350966	203554_x_at	PTTG1	pituitary tumor-transforming 1	1.31019
	Hs.82927	212360_at	AMPD2	adenosine monophosphate deaminase 2	1.3116
	Hs.269109	203789_s_at	SEMA3C	sema domain, immunoglobulin domain (Ig), short basic domain, secreted, (semaphorin) 3C	1.312
	Hs.654827	204193_at	CHKB	choline kinase beta	1.31488
	Hs.720923	221621_at	LINC00338 /// SCARNA16	long intergenic non-protein coding RNA 338 /// small Cajal body-specific RNA 16	1.31497
	Hs.523739	208922_s_at	NXF1	nuclear RNA export factor 1	1.31566
	Hs.551111	219332_at	MICALL2	MICAL-like 2	1.31606
	Hs.311190	225201_s_at	MRPL14	mitochondrial ribosomal protein L14	1.32147
	Hs.720762	217436_x_at	HLA-J	major histocompatibility complex, class I, J (pseudogene)	1.32295
	Hs.203206	223055_s_at	XPO5	exportin 5	1.32524
	Hs.435755	225075_at	PDRG1	p53 and DNA-damage regulated 1	1.32566
	Hs.529862	1556006_s_at	CSNK1A1	Casein kinase 1, alpha 1	1.32746
	Hs.597649	203988_s_at	FUT8	fucosyltransferase 8 (alpha (1,6) fucosyltransferase)	1.32809
	Hs.459790	227987_at	VPS13A	vacuolar protein sorting 13 homolog A (S. cerevisiae)	1.33169
	Hs.44693	1560145_at	MKLN1	Muskelin 1, intracellular mediator containing kelch motifs	1.33353
	---	240868_at	LOC100129406	uncharacterized LOC100129406	1.33393
	Hs.729098	230879_at	BAG2	BCL2-associated athanogene 2	1.33479
	Hs.8859	221732_at	CANT1	calcium activated nucleotidase 1	1.33596
	Hs.487458	218425_at	RNF216	ring finger protein 216	1.33609
	Hs.48513	204434_at	SPATA2	spermatogenesis associated 2	1.33672
	Hs.297304	228395_at	GLT8D1	Glycosyltransferase 8 domain containing 1	1.33756
	Hs.67896	210443_x_at	OGFR	opioid growth factor receptor	1.33838
	Hs.530595	225636_at	STAT2	signal transducer and activator of transcription 2, 113kDa	1.3409
	Hs.523875	201598_s_at	INPPL1	inositol polyphosphate phosphatase-like 1	1.34176
	Hs.517969	201284_s_at	APEH	N-acylaminoacyl-peptide hydrolase	1.3419
	Hs.29665	201561_s_at	CLSTN1	calsyntenin 1	1.34195
	Hs.200600	201771_at	SCAMP3	secretory carrier membrane protein 3	1.342
	Hs.568818	208476_s_at	FRMD4A	FERM domain containing 4A	1.34285
	Hs.408312	44563_at	WRAP53	WD repeat containing, antisense to TP53	1.34335
	Hs.643516	1554076_s_at	TMEM136	transmembrane protein 136	1.34358
	Hs.153752	201853_s_at	CDC25B	cell division cycle 25 homolog B (S. pombe)	1.3456
	Hs.381072	201489_at	PPIF	peptidylprolyl isomerase F	1.34597
	Hs.110849	1487_at	ESRRA	estrogen-related receptor alpha	1.34643
	Hs.189690	235810_at	ZNF182	zinc finger protein 182	1.34766
	Hs.436446	202655_at	MANF	mesencephalic astrocyte-derived neurotrophic factor	1.35023
	Hs.195667	204307_at	TECPR2	tectonin beta-propeller repeat containing 2	1.35025
	Hs.107911	203192_at	ABCB6	ATP-binding cassette, sub-family B (MDR/TAP), member 6	1.35063
	Hs.445534	202861_at	PER1	period homolog 1 (Drosophila)	1.35077
	Hs.127403	203364_s_at	ATG13	autophagy related 13	1.35288
	Hs.656074	224464_s_at	NUDT22	nudix (nucleoside diphosphate linked moiety X)-type motif 22	1.35451
	Hs.386567	242907_at	GBP2	guanylate binding protein 2, interferon-inducible	1.35535
	Hs.280387	208433_s_at	LRP8	low density lipoprotein receptor-related protein 8, apolipoprotein e receptor	1.35629
	Hs.659104	227657_at	RNF150	ring finger protein 150	1.35853
	Hs.195659	213324_at	SRC	v-src sarcoma (Schmidt-Ruppin A-2) viral oncogene homolog (avian)	1.35928
	Hs.112195	227468_at	CPT1C	carnitine palmitoyltransferase 1C	1.35945
	Hs.644004	221158_at	GCFC1	GC-rich sequence DNA-binding factor 1	1.3599
	Hs.730659	202128_at	KIAA0317	KIAA0317	1.36119
	Hs.113912	215992_s_at	RAPGEF2	Rap guanine nucleotide exchange factor (GEF) 2	1.3619
	Hs.12341	201786_s_at	ADAR	adenosine deaminase, RNA-specific	1.36212

	Hs.603252	1557578_at	PHLDB2	Pleckstrin homology-like domain, family B, member 2	1.36597
	Hs.467279	209179_s_at	MBOAT7	membrane bound O-acyltransferase domain containing 7	1.36822
	Hs.10499	228951_at	SLC38A7	solute carrier family 38, member 7	1.3695
	Hs.78354	206593_s_at	MED22	mediator complex subunit 22	1.36955
	Hs.124147	208989_s_at	KDM2A	lysine (K)-specific demethylase 2A	1.37033
	Hs.178728	202463_s_at	MBD3	methyl-CpG binding domain protein 3	1.37232
	Hs.655285	200045_at	ABCF1	ATP-binding cassette, sub-family F (GCN20), member 1	1.37774
	Hs.424312	211564_s_at	PDLIM4	PDZ and LIM domain 4	1.38467
	Hs.493919	210210_at	MPZL1	myelin protein zero-like 1	1.38647
	Hs.530730	224792_at	TNKS1BP1	tankyrase 1 binding protein 1, 182kDa	1.38799
	Hs.337295	213330_s_at	STIP1	stress-induced-phosphoprotein 1	1.38833
	Hs.109929	225451_at	GRIPAP1	GRIP1 associated protein 1	1.38958
	Hs.167741	204820_s_at	BTN3A2 /// BTN3A3	butyrophilin, subfamily 3, member A2 /// butyrophilin, subfamily 3, member A3	1.38992
	Hs.731529	212549_at	STAT5B	signal transducer and activator of transcription 5B	1.39191
	Hs.584748	202140_s_at	CLK3	CDC-like kinase 3	1.3926
	Hs.284491	222492_at	PDXK	pyridoxal (pyridoxine, vitamin B6) kinase	1.39262
	Hs.527524	225273_at	WWC3	WWC family member 3	1.39324
	Hs.591455	213852_at	RBM8A	RNA binding motif protein 8A	1.39423
	Hs.737838	211911_x_at	HLA-B	major histocompatibility complex, class I, B	1.39548
	Hs.595451	212618_at	ZNF609	zinc finger protein 609	1.39574
	Hs.515610	209229_s_at	PPP6R1	protein phosphatase 6, regulatory subunit 1	1.39599
	Hs.654636	220980_s_at	ADPGK	ADP-dependent glucokinase	1.39656
	Hs.708195	227125_at	IFNAR2	interferon (alpha, beta and omega) receptor 2	1.39667
	Hs.568124	202616_s_at	MECP2	methyl CpG binding protein 2 (Rett syndrome)	1.39748
	Hs.523753	218734_at	NAA40	N(alpha)-acetyltransferase 40, NatD catalytic subunit, homolog (S. cerevisiae)	1.39777
	Hs.594634	212090_at	GRINA	glutamate receptor, ionotropic, N-methyl D-aspartate-associated protein 1 (glutamate bi	1.39784
	Hs.54609	205164_at	GCAT	glycine C-acetyltransferase	1.40021
	Hs.658241	230505_at	LOC145474	uncharacterized LOC145474	1.4011
	Hs.32433	227272_at	C15orf52	chromosome 15 open reading frame 52	1.40203
	Hs.150651	218248_at	FAM111A	family with sequence similarity 111, member A	1.40208
	Hs.515417	220956_s_at	EGLN2 /// RAB4B-EGLN2	egl nine homolog 2 (C. elegans) /// RAB4B-EGLN2 readthrough	1.40259
	Hs.731416	217788_s_at	GALNT2	UDP-N-acetyl-alpha-D-galactosamine:polypeptide N-acetylgalactosaminyltransferase 2 (Gal	1.40441
	Hs.655179	218540_at	THTPA	thiamine triphosphatase	1.40657
	Hs.631886	206036_s_at	REL	v-rel reticuloendotheliosis viral oncogene homolog (avian)	1.40689
	Hs.591110	202070_s_at	IDH3A	isocitrate dehydrogenase 3 (NAD+) alpha	1.40741
	Hs.604789	209623_at	MCCC2	methylcrotonoyl-CoA carboxylase 2 (beta)	1.41434
	Hs.737838	209140_x_at	HLA-B	major histocompatibility complex, class I, B	1.417
	Hs.512465	222978_at	SURF4	surfeit 4	1.41708
	Hs.521954	203669_s_at	DGAT1	diacylglycerol O-acyltransferase 1	1.41912
	Hs.512651	224969_at	ATXN7L3	ataxin 7-like 3	1.42008
	Hs.591787	227456_s_at	C6orf136	chromosome 6 open reading frame 136	1.42213
	Hs.433343	207435_s_at	SRRM2	serine/arginine repetitive matrix 2	1.42224
	Hs.424552	239581_at	ARL10	ADP-ribosylation factor-like 10	1.4281
	Hs.730704	225218_at	ZFYVE27	zinc finger, FYVE domain containing 27	1.42984
	Hs.458355	208747_s_at	C1S	complement component 1, s subcomponent	1.43081
	Hs.403790	223192_at	SLC25A28	solute carrier family 25 (mitochondrial iron transporter), member 28	1.43431
	Hs.76090	201207_at	TNFAIP1	tumor necrosis factor, alpha-induced protein 1 (endothelial)	1.4344
	Hs.153299	215982_s_at	DOM3Z	dom-3 homolog Z (C. elegans)	1.43808
	Hs.496098	233933_s_at	OTUD5	OTU domain containing 5	1.44217
	Hs.650680	228445_at	AIFM2	apoptosis-inducing factor, mitochondrion-associated, 2	1.44248
	Hs.46679	227667_at	CUEDC1	CUE domain containing 1	1.44332

Hs.514284	200758_s_at	NFE2L1	nuclear factor (erythroid-derived 2)-like 1	1.44413
Hs.490181	238418_at	SLC35B4	solute carrier family 35, member B4	1.4446
Hs.355753	224776_at	AGPAT6	1-acylglycerol-3-phosphate O-acyltransferase 6 (lysophosphatidic acid acyltransferase,	1.44715
Hs.131342	223710_at	CCL26	chemokine (C-C motif) ligand 26	1.44838
Hs.519818	201126_s_at	MGAT1	mannosyl (alpha-1,3-)-glycoprotein beta-1,2-N-acetylglucosaminyltransferase	1.45042
Hs.371210	222720_x_at	C1orf27	chromosome 1 open reading frame 27	1.45438
Hs.438720	210983_s_at	MCM7	minichromosome maintenance complex component 7	1.45517
Hs.1570	205579_at	HRH1	histamine receptor H1	1.45698
Hs.371794	225076_s_at	ZNFX1	zinc finger, NFX1-type containing 1	1.45719
Hs.65735	203709_at	PHKG2	phosphorylase kinase, gamma 2 (testis)	1.45796
Hs.500013	202865_at	DNAJB12	DnaJ (Hsp40) homolog, subfamily B, member 12	1.45802
Hs.284284	225733_at	B3GALT6	UDP-Gal:betaGal beta 1,3-galactosyltransferase polypeptide 6	1.45995
Hs.85155	211965_at	ZFP36L1	zinc finger protein 36, C3H type-like 1	1.463
Hs.662150	220710_at	ANP32A-IT1	ANP32A intronic transcript 1 (non-protein coding)	1.46452
Hs.709864	223622_s_at	HYI	hydroxypyruvate isomerase (putative)	1.46527
Hs.500466	233254_x_at	PTEN	phosphatase and tensin homolog	1.4672
Hs.731801	209100_at	IFRD2	interferon-related developmental regulator 2	1.47208
Hs.521151	218068_s_at	ZNF672	zinc finger protein 672	1.47434
Hs.326035	201693_s_at	EGR1	early growth response 1	1.47473
Hs.26010	201037_at	PFKP	phosphofructokinase, platelet	1.4763
Hs.517948	204355_at	DHX30	DEAH (Asp-Glu-Ala-His) box polypeptide 30	1.47659
Hs.202354	231240_at	DIO2	deiodinase, iodothyronine, type II	1.47665
Hs.502872	203652_at	MAP3K11	mitogen-activated protein kinase kinase kinase 11	1.47921
Hs.396189	239203_at	C7orf53	chromosome 7 open reading frame 53	1.47925
Hs.178715	229687_s_at	PRDM11	PR domain containing 11	1.48049
Hs.336810	223132_s_at	TRIM8	tripartite motif containing 8	1.48511
Hs.515493	227882_at	FKRP	fukutin related protein	1.48538
Hs.34024	213107_at	TNIK	TRAF2 and NCK interacting kinase	1.48589
Hs.459538	203180_at	ALDH1A3	aldehyde dehydrogenase 1 family, member A3	1.48714
Hs.193133	213236_at	SASH1	SAM and SH3 domain containing 1	1.48824
Hs.467587	1569519_at	LOC100506032 /// NBPF1 /// NBPF10 /// NBPF11 /// NBPF12 /// NBPF24 /// NBPF7 /// NBPF8 /// NBPF9	neuroblastoma breakpoint family member 21-like /// neuroblastoma breakpoint family, mem	1.49012
Hs.632447	202250_s_at	DCAF8	DDB1 and CUL4 associated factor 8	1.49032
Hs.520612	226206_at	MAFK	v-maf musculoaponeurotic fibrosarcoma oncogene homolog K (avian)	1.49076
Hs.737838	208729_x_at	HLA-B	major histocompatibility complex, class I, B	1.49857
Hs.591947	203759_at	ST3GAL4	ST3 beta-galactoside alpha-2,3-sialyltransferase 4	1.50124
Hs.10499	218727_at	SLC38A7	solute carrier family 38, member 7	1.50548
Hs.731416	217787_s_at	GALNT2	UDP-N-acetyl-alpha-D-galactosamine:polypeptide N-acetylgalactosaminyltransferase 2 (Gal	1.50595
Hs.492618	230183_at	EXT1	exostosin 1	1.5093
Hs.266728	204800_s_at	DHRS12	dehydrogenase/reductase (SDR family) member 12	1.51062
Hs.487175	214124_x_at	FGFR1OP	FGFR1 oncogene partner	1.512
Hs.119878	238025_at	MLKL	mixed lineage kinase domain-like	1.5126
Hs.271977	220559_at	EN1	engrailed homeobox 1	1.51526
Hs.530477	204164_at	SIPA1	signal-induced proliferation-associated 1	1.51766
Hs.519972	204806_x_at	HLA-F	major histocompatibility complex, class I, F	1.52075
Hs.116448	223080_at	GLS	Glutaminase	1.52234
Hs.283739	224513_s_at	UBQLN4	ubiquilin 4	1.52334
Hs.89890	204476_s_at	PC	pyruvate carboxylase	1.52418

	Hs.445534	36829_at	PER1	period homolog 1 (Drosophila)	1.52544
	Hs.484741	204187_at	GMPR	guanosine monophosphate reductase	1.52574
	Hs.171844	212662_at	PVR	poliovirus receptor	1.52742
	Hs.233160	203439_s_at	STC2	stanniocalcin 2	1.52746
	Hs.654636	224455_s_at	ADPGK	ADP-dependent glucokinase	1.53737
	Hs.740403	213338_at	TMEM158	transmembrane protein 158 (gene/pseudogene)	1.53768
	Hs.558764	201762_s_at	PSME2	proteasome (prosome, macropain) activator subunit 2 (PA28 beta)	1.53842
	Hs.173233	219230_at	TMEM100	transmembrane protein 100	1.53922
	Hs.446429	211663_x_at	PTGDS	prostaglandin D2 synthase 21kDa (brain)	1.54077
	Hs.632472	202963_at	RFX5	regulatory factor X, 5 (influences HLA class II expression)	1.54429
	Hs.376046	212613_at	BTN3A2	butyrophilin, subfamily 3, member A2	1.54558
	Hs.123534	204341_at	TRIM16	tripartite motif containing 16	1.54634
	Hs.288467	213909_at	LRRC15	leucine rich repeat containing 15	1.54967
	Hs.503546	208962_s_at	FADS1 /// MIR1908	fatty acid desaturase 1 /// microRNA 1908	1.55149
	Hs.517670	216251_s_at	TTLL12	tubulin tyrosine ligase-like family, member 12	1.55292
	Hs.75348	200814_at	PSME1	proteasome (prosome, macropain) activator subunit 1 (PA28 alpha)	1.55319
	Hs.408730	219400_at	CNTNAP1	contactin associated protein 1	1.55773
	Hs.579079	213009_s_at	TRIM37	tripartite motif containing 37	1.55814
	Hs.167451	204155_s_at	SIK3	SIK family kinase 3	1.56311
	Hs.733289	229095_s_at	LIMS3 /// LIMS3-LOC440895 /// LIMS3L /// LOC100288570 /// LOC100507334 /// LOC440895	LIM and senescent cell antigen-like domains 3 /// LIMS3-LOC440895 readthrough /// LIM a	1.56468
	Hs.471991	205323_s_at	MTF1	metal-regulatory transcription factor 1	1.57157
	Hs.146274	229715_at	B7H6	B7 homolog 6	1.57261
	Hs.731427	201471_s_at	SQSTM1	sequestosome 1	1.57531
	Hs.730686	208047_s_at	NAB1	NGFI-A binding protein 1 (EGR1 binding protein 1)	1.57531
	Hs.656274	208296_x_at	TNFAIP8	tumor necrosis factor, alpha-induced protein 8	1.57909
	Hs.702167	201482_at	QSOX1	quiescin Q6 sulfhydryl oxidase 1	1.58042
	Hs.118695	214595_at	KCNG1	potassium voltage-gated channel, subfamily G, member 1	1.5842
	Hs.183800	212127_at	RANGAP1	Ran GTPase activating protein 1	1.5851
	Hs.250493	219314_s_at	ZNF219	zinc finger protein 219	1.58544
	Hs.540696	213843_x_at	SLC6A8	solute carrier family 6 (neurotransmitter transporter, creatine), member 8	1.58581
	Hs.534667	210859_x_at	CLN3	ceroid-lipofuscinosis, neuronal 3	1.58743
	Hs.514012	207667_s_at	MAP2K3	mitogen-activated protein kinase kinase 3	1.59045
	Hs.386225	201673_s_at	GYS1	glycogen synthase 1 (muscle)	1.59215
	Hs.89864	203727_at	SKIV2L	superkiller viralicidic activity 2-like (S. cerevisiae)	1.59465
	Hs.285666	231823_s_at	SH3PXD2B	SH3 and PX domains 2B	1.5967
	Hs.71040	223025_s_at	AP1M1	adaptor-related protein complex 1, mu 1 subunit	1.59931
	Hs.521482	1557458_s_at	SHB	Src homology 2 domain containing adaptor protein B	1.6009
	Hs.133512	220104_at	ZC3HAV1	zinc finger CCCH-type, antiviral 1	1.60137
	Hs.514284	200759_x_at	NFE2L1	nuclear factor (erythroid-derived 2)-like 1	1.60212
	Hs.584841	214130_s_at	LOC728802 /// PDE4DIP	myomegalin-like /// phosphodiesterase 4D interacting protein	1.60468
	Hs.591638	231252_at	KANSL1L	KAT8 regulatory NSL complex subunit 1-like	1.61014
	Hs.567572	232879_at	CRTC3	CREB regulated transcription coactivator 3	1.61178
	Hs.277704	200825_s_at	HYOU1	hypoxia up-regulated 1	1.61621
	Hs.592304	225750_at	ERO1L	ERO1-like (S. cerevisiae)	1.61934
	Hs.477420	212822_at	HEG1	HEG homolog 1 (zebrafish)	1.61957
	Hs.163642	233072_at	NTNG2	netrin G2	1.63043
	Hs.386684	244699_at	AHI1	Abelson helper integration site 1	1.63607
	Hs.193163	202931_x_at	BIN1	bridging integrator 1	1.63928

	Hs.232021	238736_at	REV3L	REV3-like, catalytic subunit of DNA polymerase zeta (yeast)	1.64537
	Hs.444950	226613_at	GATSL3	GATS protein-like 3	1.64664
	Hs.193163	210202_s_at	BIN1	bridging integrator 1	1.65019
	Hs.436792	209204_at	LMO4	LIM domain only 4	1.65573
	Hs.731394	203066_at	CHST15	carbohydrate (N-acetylgalactosamine 4-sulfate 6-O) sulfotransferase 15	1.65786
	Hs.740679	238949_at	RNF145	ring finger protein 145	1.65977
	Hs.500761	202856_s_at	SLC16A3	solute carrier family 16, member 3 (monocarboxylic acid transporter 4)	1.6644
	Hs.530595	205170_at	STAT2	signal transducer and activator of transcription 2, 113kDa	1.66633
	Hs.494457	203045_at	NINJ1	ninjurin 1	1.68158
	Hs.420559	202392_s_at	PISD	phosphatidylserine decarboxylase	1.68746
	Hs.284491	218019_s_at	PDXK	pyridoxal (pyridoxine, vitamin B6) kinase	1.68762
	Hs.521482	204656_at	SHB	Src homology 2 domain containing adaptor protein B	1.68876
	Hs.656274	210260_s_at	TNFAIP8	tumor necrosis factor, alpha-induced protein 8	1.69017
	Hs.595793	39548_at	NPAS2	neuronal PAS domain protein 2	1.69659
	Hs.21765	216080_s_at	FADS3	fatty acid desaturase 3	1.70678
	Hs.530381	224739_at	PIM3	pim-3 oncogene	1.70921
	Hs.403790	221432_s_at	SLC25A28	solute carrier family 25 (mitochondrial iron transporter), member 28	1.72304
	Hs.642990	209969_s_at	STAT1	signal transducer and activator of transcription 1, 91kDa	1.72543
	Hs.19987	219383_at	PRR5L	proline rich 5 like	1.72884
	Hs.459649	209235_at	CLCN7	chloride channel, voltage-sensitive 7	1.72923
	Hs.109225	203868_s_at	VCAM1	vascular cell adhesion molecule 1	1.73545
	Hs.406530	223113_at	TMEM138	transmembrane protein 138	1.73926
	Hs.729056	212892_at	ZNF282	zinc finger protein 282	1.73972
	Hs.514284	214179_s_at	NFE2L1	nuclear factor (erythroid-derived 2)-like 1	1.7417
	Hs.1706	203882_at	IRF9	interferon regulatory factor 9	1.75317
	Hs.310640	226117_at	TIFA	TRAF-interacting protein with forkhead-associated domain	1.754
	Hs.99962	210692_s_at	SLC43A3	solute carrier family 43, member 3	1.75551
	Hs.352018	202307_s_at	TAP1	transporter 1, ATP-binding cassette, sub-family B (MDR/TAP)	1.75853
	Hs.287362	228340_at	TLE3	transducin-like enhancer of split 3 (E(sp1) homolog, Drosophila)	1.7605
	Hs.502458	1558965_at	PHF21A	PHD finger protein 21A	1.77099
	Hs.631858	219952_s_at	MCOLN1	mucolipin 1	1.77772
	Hs.60640	242957_at	VWCE	von Willebrand factor C and EGF domains	1.78253
	Hs.478275	214329_x_at	TNFSF10	tumor necrosis factor (ligand) superfamily, member 10	1.78609
	Hs.193163	214439_x_at	BIN1	bridging integrator 1	1.80897
	Hs.127432	236649_at	DTWD1	DTW domain containing 1	1.82506
	Hs.604355	209870_s_at	APBA2	amyloid beta (A4) precursor protein-binding, family A, member 2	1.86556
	Hs.586109	232370_at	LOC254057	uncharacterized LOC254057	1.88596
	Hs.740543	219247_s_at	ZDHHC14	zinc finger, DHHC-type containing 14	1.89382
	Hs.122785	206522_at	MGAM	maltase-glucoamylase (alpha-glucosidase)	1.90428
	Hs.167641	225058_at	GPR108	G protein-coupled receptor 108	1.91074
	Hs.443831	227751_at	PDCD5	programmed cell death 5	1.9141
	---	1556936_at	LOC100506834	uncharacterized LOC100506834	1.91547
	Hs.408702	1554016_a_at	C16orf57	chromosome 16 open reading frame 57	1.93224
	Hs.711617	220739_s_at	CNNM3	cyclin M3	1.97685
	Hs.79015	209582_s_at	CD200	CD200 molecule	1.97937
	Hs.522378	225020_at	DAB2IP	DAB2 interacting protein	1.98051
	Hs.524183	200894_s_at	FKBP4	FK506 binding protein 4, 59kDa	1.98436
	Hs.664877	232568_at	MGC24103	uncharacterized MGC24103	1.98479
	Hs.436061	202531_at	IRF1	interferon regulatory factor 1	1.99795
	Hs.68061	219257_s_at	SPHK1	sphingosine kinase 1	2.00807
	Hs.293660	225868_at	TRIM47	tripartite motif containing 47	2.01547
	Hs.614406	202340_x_at	NR4A1	nuclear receptor subfamily 4, group A, member 1	2.01922
	Hs.517227	229127_at	JAM2	junctional adhesion molecule 2	2.02183

	Hs.511915	201313_at	ENO2	enolase 2 (gamma, neuronal)	2.03284
	Hs.580681	204502_at	SAMHD1	SAM domain and HD domain 1	2.043
	Hs.593171	226225_at	MCC	mutated in colorectal cancers	2.04783
	Hs.620021	204288_s_at	SORBS2	sorbin and SH3 domain containing 2	2.05393
	Hs.105448	229158_at	WNK4	WNK lysine deficient protein kinase 4	2.0653
	Hs.130759	202430_s_at	PLSCR1	phospholipid scramblase 1	2.06803
	Hs.130759	202446_s_at	PLSCR1	phospholipid scramblase 1	2.08345
	Hs.1027	204802_at	RRAD	Ras-related associated with diabetes	2.09034
	Hs.474705	202807_s_at	TOM1	target of myb1 (chicken)	2.14039
	Hs.522507	223050_s_at	FBXW5	F-box and WD repeat domain containing 5	2.14835
	Hs.370937	208829_at	TAPBP	TAP binding protein (tapasin)	2.15759
	Hs.321045	204549_at	IKBKE	inhibitor of kappa light polypeptide gene enhancer in B-cells, kinase epsilon	2.16018
	Hs.591873	206693_at	IL7	interleukin 7	2.16944
	Hs.502	225973_at	TAP2	transporter 2, ATP-binding cassette, sub-family B (MDR/TAP)	2.21937
	Hs.631925	221572_s_at	SLC26A6	solute carrier family 26, member 6	2.22181
	Hs.319171	223218_s_at	NFKBIZ	nuclear factor of kappa light polypeptide gene enhancer in B-cells inhibitor, zeta	2.25228
	Hs.696497	205100_at	GFPT2	glutamine-fructose-6-phosphate transaminase 2	2.26359
	Hs.601143	223290_at	PDXP /// SH3BP1	pyridoxal (pyridoxine, vitamin B6) phosphatase /// SH3-domain binding protein 1	2.27512
	Hs.2490	206011_at	CASP1	caspase 1, apoptosis-related cysteine peptidase	2.27827
	Hs.355141	207196_s_at	TNIP1	TNFAIP3 interacting protein 1	2.28841
	Hs.17569	226372_at	CHST11	carbohydrate (chondroitin 4) sulfotransferase 11	2.32497
	Hs.319171	223217_s_at	NFKBIZ	nuclear factor of kappa light polypeptide gene enhancer in B-cells inhibitor, zeta	2.33248
	Hs.484047	236725_at	WWC1	WW and C2 domain containing 1	2.3425
	Hs.408702	218060_s_at	C16orf57	chromosome 16 open reading frame 57	2.36573
	Hs.22546	224735_at	CYBASC3	cytochrome b, ascorbate dependent 3	2.38265
	Hs.500761	202855_s_at	SLC16A3	solute carrier family 16, member 3 (monocarboxylic acid transporter 4)	2.4053
	Hs.20395	218829_s_at	CHD7	chromodomain helicase DNA binding protein 7	2.40601
	Hs.127022	221840_at	PTPRE	protein tyrosine phosphatase, receptor type, E	2.46239
	Hs.515383	1558404_at	LOC644242	uncharacterized LOC644242	2.46956
	Hs.336916	201763_s_at	DAXX	death-domain associated protein	2.5265
	Hs.524293	203074_at	ANXA8 /// ANXA8L1 /// ANXA8L2	annexin A8 /// annexin A8-like 1 /// annexin A8-like 2	2.52734
	Hs.180903	217497_at	TYMP	thymidine phosphorylase	2.55251
	Hs.517617	205193_at	MAFF	v-maf musculoaponeurotic fibrosarcoma oncogene homolog F (avian)	2.61253
	Hs.740396	202897_at	SIRPA	signal-regulatory protein alpha	2.65291
	Hs.655143	233720_at	SORBS2	Sorbin and SH3 domain containing 2	2.75661
	Hs.237856	219593_at	SLC15A3	solute carrier family 15, member 3	2.76827
	Hs.166120	208436_s_at	IRF7	interferon regulatory factor 7	2.80105
	Hs.443728	243582_at	SH3RF2	SH3 domain containing ring finger 2	2.81878
	Hs.186649	213142_x_at	PION	pigeon homolog (Drosophila)	2.83149
	Hs.210995	210735_s_at	CA12	carbonic anhydrase XII	2.83741
	Hs.186649	222150_s_at	PION	pigeon homolog (Drosophila)	2.89871
	Hs.520101	237016_at	TMEM217	transmembrane protein 217	3.11311
	Hs.753	205119_s_at	FPR1	formyl peptide receptor 1	3.11483
	Hs.417962	226034_at	DUSP4	dual specificity phosphatase 4	3.1355
	Hs.442619	209928_s_at	MSC	musculin	3.18359
	Hs.373550	1566901_at	TGIF1	TGFB-induced factor homeobox 1	3.33847
	Hs.654542	205870_at	BDKRB2	bradykinin receptor B2	3.36972
	Hs.476218	217312_s_at	COL7A1	collagen, type VII, alpha 1	3.39883
	Hs.118110	201641_at	BST2	bone marrow stromal cell antigen 2	3.59075
	Hs.190622	218943_s_at	DDX58	DEAD (Asp-Glu-Ala-Asp) box polypeptide 58	3.62404
	Hs.160562	209541_at	IGF1	insulin-like growth factor 1 (somatomedin C)	3.64701

Hs.374257	225033_at	ST3GAL1	ST3 beta-galactoside alpha-2,3-sialyltransferase 1	3.73223
Hs.82316	214453_s_at	IFI44	interferon-induced protein 44	3.79557
Hs.546467	227609_at	EPSTI1	epithelial stromal interaction 1 (breast)	4.0458
Hs.471200	211844_s_at	NRP2	neuropilin 2	4.14217
Hs.525607	202510_s_at	TNFAIP2	tumor necrosis factor, alpha-induced protein 2	4.23039
Hs.195040	205404_at	HSD11B1	hydroxysteroid (11-beta) dehydrogenase 1	4.72578
Hs.81328	201502_s_at	NFKBIA	nuclear factor of kappa light polypeptide gene enhancer in B-cells inhibitor, alpha	4.74509
Hs.437322	206025_s_at	TNFAIP6	tumor necrosis factor, alpha-induced protein 6	4.84297
Hs.389724	204439_at	IFI44L	interferon-induced protein 44-like	5.04887
Hs.731813	229450_at	IFIT3	interferon-induced protein with tetratricopeptide repeats 3	5.10923
Hs.730800	223454_at	CXCL16	chemokine (C-X-C motif) ligand 16	5.30855
Hs.163173	219209_at	IFIH1	interferon induced with helicase C domain 1	5.39069
Hs.458485	205483_s_at	ISG15	ISG15 ubiquitin-like modifier	5.4589
Hs.546467	235276_at	EPSTI1	epithelial stromal interaction 1 (breast)	5.46917
Hs.211600	202644_s_at	TNFAIP3	tumor necrosis factor, alpha-induced protein 3	5.47167
Hs.523847	204415_at	IFI6	interferon, alpha-inducible protein 6	5.68202
Hs.654402	205205_at	RELB	v-rel reticuloendotheliosis viral oncogene homolog B	6.37181
Hs.1395	205249_at	EGR2	early growth response 2	7.10657
Hs.303649	216598_s_at	CCL2	chemokine (C-C motif) ligand 2	7.80961
Hs.414332	204972_at	OAS2	2'-5'-oligoadenylate synthetase 2, 69/71kDa	7.81544
Hs.528634	218400_at	OAS3	2'-5'-oligoadenylate synthetase 3, 100kDa	9.05016
Hs.25590	204595_s_at	STC1	stanniocalcin 1	9.80787
Hs.524760	205552_s_at	OAS1	2'-5'-oligoadenylate synthetase 1, 40/46kDa	11.3964
Hs.487046	221477_s_at	LOC100129518 /// SOD2	uncharacterized LOC100129518 /// superoxide dismutase 2, mitochondrial	13.5602
Hs.7155	226702_at	CMPK2	cytidine monophosphate (UMP-CMP) kinase 2, mitochondrial	15.4655
Hs.926	204994_at	MX2	myxovirus (influenza virus) resistance 2 (mouse)	15.6128
Hs.529317	219352_at	HERC6	HECT and RLD domain containing E3 ubiquitin protein ligase family member 6	15.7442
Hs.643447	202638_s_at	ICAM1	intercellular adhesion molecule 1	16.68
Hs.69771	202357_s_at	CFB	complement factor B	20.7019
Hs.75765	209774_x_at	CXCL2	chemokine (C-X-C motif) ligand 2	20.9874
Hs.487046	215223_s_at	LOC100129518 /// SOD2	uncharacterized LOC100129518 /// superoxide dismutase 2, mitochondrial	23.8639
Hs.529053	217767_at	C3	complement component 3	25.6036
Hs.517307	202086_at	MX1	myxovirus (influenza virus) resistance 1, interferon-inducible protein p78 (mouse)	43.4907
Hs.789	204470_at	CXCL1	chemokine (C-X-C motif) ligand 1 (melanoma growth stimulating activity, alpha)	63.446
Hs.89690	207850_at	CXCL3	chemokine (C-X-C motif) ligand 3	69.0519
Hs.624	202859_x_at	IL8	interleukin 8	93.437
Hs.624	211506_s_at	IL8	interleukin 8	205.742

Supplemental Table 2. Genes differentially expressed in primed Resto cells compared with unprimed Resto cells. Green line indicates genes overexpressed in unprimed Resto cells and red line indicates genes overexpressed in primed Resto cells. Fold Change corresponds to the ratio of median expression in PMN-primed / unprimed Resto cells.

	UniGene ID	Probeset ID	Gene Symbol	Gene Title	FoldChange
	Hs.87191	211029_x_at	FGF18	fibroblast growth factor 18	-11.3298
	Hs.76392	212224_at	ALDH1A1	aldehyde dehydrogenase 1 family, member A1	-9.05956
	Hs.87191	206987_x_at	FGF18	fibroblast growth factor 18	-7.7272
	Hs.87191	231382_at	FGF18	fibroblast growth factor 18	-6.83921
	Hs.94070	205908_s_at	OMD	osteomodulin	-6.49102
	Hs.591282	230867_at	COL6A6	collagen, type VI, alpha 6	-3.88184
	Hs.533670	206349_at	LGI1	leucine-rich, glioma inactivated 1	-3.8148
	Hs.4	209612_s_at	ADH1B	alcohol dehydrogenase 1B (class I), beta polypeptide	-3.74855
	Hs.446077	220786_s_at	SLC38A4	solute carrier family 38, member 4	-3.71169
	Hs.591352	227475_at	FOXQ1	forkhead box Q1	-3.50818
	Hs.479754	205051_s_at	KIT	v-kit Hardy-Zuckerman 4 feline sarcoma viral oncogene homolog	-3.47075
	Hs.673160	1553630_at	C10orf107	chromosome 10 open reading frame 107	-3.29609
	Hs.520989	227265_at	FGL2	fibrinogen-like 2	-3.07527
	Hs.567973	228653_at	SAMD5	sterile alpha motif domain containing 5	-3.07336
	Hs.58324	229357_at	ADAMTS5	ADAM metallopeptidase with thrombospondin type 1 motif, 5	-3.05926
	Hs.655515	1552365_at	SCIN	scinderin	-2.75919
	Hs.499725	206385_s_at	ANK3	ankyrin 3, node of Ranvier (ankyrin G)	-2.68982
	Hs.58324	219935_at	ADAMTS5	ADAM metallopeptidase with thrombospondin type 1 motif, 5	-2.56013
	Hs.26530	222717_at	SDPR	serum deprivation response	-2.48192
	Hs.496755	228889_at	ARHGAP5-AS1	ARHGAP5 antisense RNA 1 (non-protein coding)	-2.47461
	Hs.48029	219480_at	SNAI1	snail homolog 1 (Drosophila)	-2.44471
	Hs.92489	220115_s_at	CDH10	cadherin 10, type 2 (T2-cadherin)	-2.41906
	Hs.499725	209442_x_at	ANK3	ankyrin 3, node of Ranvier (ankyrin G)	-2.4143
	Hs.156316	240556_at	DCN	decorin	-2.41312
	Hs.117060	206101_at	ECM2	extracellular matrix protein 2, female organ and adipocyte specific	-2.41236
	Hs.659934	235683_at	SESN3	sestrin 3	-2.40392
	Hs.5333	204301_at	KBTBD11	kelch repeat and BTB (POZ) domain containing 11	-2.3975
	Hs.411488	241703_at	RUNDC3B	RUN domain containing 3B	-2.39092
	Hs.732776	227984_at	LMF1	lipase maturation factor 1	-2.37904
	Hs.352298	219304_s_at	PDGFD	platelet derived growth factor D	-2.37249
	Hs.643005	223044_at	SLC40A1	solute carrier family 40 (iron-regulated transporter), member 1	-2.34315
	Hs.335293	1560477_a_at	SAMD11	sterile alpha motif domain containing 11	-2.31662
	Hs.534221	229004_at	ADAMTS15	ADAM metallopeptidase with thrombospondin type 1 motif, 15	-2.28166
	Hs.208093	235301_at	KIAA1324L	KIAA1324-like	-2.25763
	Hs.520989	204834_at	FGL2	fibrinogen-like 2	-2.23504
	Hs.95120	226632_at	CYGB	cytoglobin	-2.22236
	Hs.44385	213413_at	STON1	stonin 1	-2.21425
	Hs.244940	227467_at	RDH10	retinol dehydrogenase 10 (all-trans)	-2.19335
	Hs.368281	210015_s_at	MAP2	microtubule-associated protein 2	-2.18938
	Hs.486508	244353_s_at	SLC2A12	solute carrier family 2 (facilitated glucose transporter), member 12	-2.17444
	Hs.152944	205011_at	VWA5A	von Willebrand factor A domain containing 5A	-2.1568
	Hs.135118	230311_s_at	PRDM6	PR domain containing 6	-2.15422
	Hs.91546	219825_at	CYP26B1	cytochrome P450, family 26, subfamily B, polypeptide 1	-2.142
	Hs.700228	204284_at	PPP1R3C	protein phosphatase 1, regulatory subunit 3C	-2.14086
	Hs.253146	229245_at	PLEKHA6	pleckstrin homology domain containing, family A member 6	-2.11399
	Hs.440168	203498_at	RCAN2	regulator of calcineurin 2	-2.11033

	Hs.623400	218353_at	RGS5	regulator of G-protein signaling 5	-2.11008
	Hs.494977	212830_at	MEGF9	multiple EGF-like-domains 9	-2.10127
	Hs.106070	213348_at	CDKN1C	cyclin-dependent kinase inhibitor 1C (p57, Kip2)	-2.09709
	Hs.518989	225977_at	PCDH18	protocadherin 18	-2.09455
	---	226591_at	LOC100506965	uncharacterized LOC100506965	-2.09157
	Hs.26409	235494_at	LSAMP	limbic system-associated membrane protein	-2.0785
	Hs.272367	241355_at	HR	hairless homolog (mouse)	-2.07801
	Hs.78183	209160_at	AKR1C3	aldo-keto reductase family 1, member C3 (3-alpha hydroxysteroid dehydrogenase, type II)	-2.06799
	Hs.386791	228507_at	PDE3A	phosphodiesterase 3A, cGMP-inhibited	-2.06416
	Hs.200644	1555980_a_at	LOC100130417	Uncharacterized LOC100130417	-2.06382
	Hs.259559	229222_at	ACSS3	acyl-CoA synthetase short-chain family member 3	-2.0495
	Hs.162016	224724_at	SULF2	sulfatase 2	-2.03122
	Hs.150122	219073_s_at	OSBPL10	oxysterol binding protein-like 10	-2.02856
	Hs.40510	1552774_a_at	SLC25A27	solute carrier family 25, member 27	-2.02556
	Hs.149940	235776_x_at	LINC00475	long intergenic non-protein coding RNA 475	-2.00957
	Hs.156727	220076_at	ANKH	ankylosis, progressive homolog (mouse)	-2.00707
	Hs.610520	201427_s_at	SEPP1	selenoprotein P, plasma, 1	-2.00477
	Hs.244940	226021_at	RDH10	retinol dehydrogenase 10 (all-trans)	-2.00389
	Hs.584776	206163_at	MAB21L1	mab-21-like 1 (C. elegans)	-1.9866
	Hs.655519	227662_at	SYNPO2	synaptopodin 2	-1.98124
	Hs.132576	231145_at	PAX9	paired box 9	-1.97275
	Hs.631789	238684_at	SETDB2	SET domain, bifurcated 2	-1.97241
	Hs.26670	221756_at	PIK3IP1	phosphoinositide-3-kinase interacting protein 1	-1.96702
	Hs.491805	204529_s_at	TOX	thymocyte selection-associated high mobility group box	-1.93329
	Hs.444414	227198_at	AFF3	AF4/FMR2 family, member 3	-1.90972
	Hs.253247	213568_at	OSR2	odd-skipped related 2 (Drosophila)	-1.90093
	---	229296_at	LOC100506119	uncharacterized LOC100506119	-1.89804
	Hs.27092	228739_at	CYS1	cystin 1	-1.89259
	Hs.659300	220441_at	DNAJC22	DnaJ (Hsp40) homolog, subfamily C, member 22	-1.88837
	Hs.631504	203185_at	RASSF2	Ras association (RalGDS/AF-6) domain family member 2	-1.88404
	Hs.471610	219636_s_at	ARMC9	armadillo repeat containing 9	-1.87615
	Hs.380094	234971_x_at	PLCD3	phospholipase C, delta 3	-1.86104
	Hs.197043	218918_at	MAN1C1	mannosidase, alpha, class 1C, member 1	-1.85674
	Hs.602792	240898_at	SPAG16	sperm associated antigen 16	-1.85511
	Hs.485104	216333_x_at	TNXA /// TNXB	tenascin XA (pseudogene) /// tenascin XB	-1.84567
	Hs.534612	230266_at	RAB7B	RAB7B, member RAS oncogene family	-1.84104
	Hs.438782	229408_at	HDAC5	histone deacetylase 5	-1.83494
	Hs.61329	1558507_at	C1orf53	chromosome 1 open reading frame 53	-1.83458
	Hs.633506	228347_at	SIX1	SIX homeobox 1	-1.8298
	Hs.592184	203106_s_at	VPS41	vacuolar protein sorting 41 homolog (S. cerevisiae)	-1.82834
	Hs.549204	229890_at	LOC100507547 /// PRRT1	uncharacterized LOC100507547 /// proline-rich transmembrane protein 1	-1.82565
	---	227115_at	LOC100506870	uncharacterized LOC100506870	-1.82245
	Hs.213137	242100_at	CHSY3	chondroitin sulfate synthase 3	-1.82083
	Hs.563205	219427_at	FAT4	FAT tumor suppressor homolog 4 (Drosophila)	-1.8136
	Hs.122055	229146_at	C7orf31	chromosome 7 open reading frame 31	-1.79684
	Hs.124537	241745_at	LOC100507557	uncharacterized LOC100507557	-1.79474
	Hs.567598	221011_s_at	LBH	limb bud and heart development homolog (mouse)	-1.7907
	Hs.284217	223282_at	TSHZ1	teashirt zinc finger homeobox 1	-1.78592
	Hs.503500	217525_at	OLFML1	olfactomedin-like 1	-1.77879
	Hs.740550	225056_at	SIPA1L2	signal-induced proliferation-associated 1 like 2	-1.77314

	Hs.312592	232531_at	EMX2OS	EMX2 opposite strand/antisense RNA (non-protein coding)	-1.75929
	Hs.42502	230147_at	F2RL2	coagulation factor II (thrombin) receptor-like 2	-1.7592
	Hs.632559	237116_at	LOC646903	uncharacterized LOC646903	-1.75868
	Hs.146180	213285_at	TMEM30B	transmembrane protein 30B	-1.75853
	Hs.1501	212157_at	SDC2	syndecan 2	-1.75843
	Hs.477375	1569956_at	MYLK	myosin light chain kinase	-1.75171
	Hs.154654	202437_s_at	CYP1B1	cytochrome P450, family 1, subfamily B, polypeptide 1	-1.7413
	Hs.296049	212713_at	MFAP4	microfibrillar-associated protein 4	-1.73732
	Hs.722375	244065_at	LOC643792	contactin associated protein-like 3 pseudogene	-1.72839
	Hs.360174	213139_at	SNAI2	snail homolog 2 (Drosophila)	-1.72335
	Hs.419	207147_at	DLX2	distal-less homeobox 2	-1.71505
	Hs.2799	205523_at	HAPLN1	hyaluronan and proteoglycan link protein 1	-1.71462
	Hs.284217	223283_s_at	TSHZ1	teashirt zinc finger homeobox 1	-1.71329
	Hs.657163	227611_at	TARSL2	threonyl-tRNA synthetase-like 2	-1.70918
	Hs.268515	205330_at	MN1	meningioma (disrupted in balanced translocation) 1	-1.70544
	Hs.1420	204379_s_at	FGFR3	fibroblast growth factor receptor 3	-1.70324
	Hs.64746	219529_at	CLIC3	chloride intracellular channel 3	-1.70227
	Hs.113577	210631_at	NF1	neurofibromin 1	-1.70105
	Hs.518989	225975_at	PCDH18	protocadherin 18	-1.6903
	Hs.200841	205116_at	LAMA2	laminin, alpha 2	-1.68055
	Hs.511265	226492_at	SEMA6D	sema domain, transmembrane domain (TM), and cytoplasmic domain, (semaphorin) 6D	-1.67935
	Hs.102735	238484_s_at	SSBP2	single-stranded DNA binding protein 2	-1.66961
	Hs.494538	209815_at	PTCH1	patched 1	-1.66722
	---	241418_at	LOC344887	NmrA-like family domain containing 1 pseudogene	-1.66606
	Hs.1501	212158_at	SDC2	syndecan 2	-1.66421
	Hs.348522	222379_at	KCNE4	potassium voltage-gated channel, Isk-related family, member 4	-1.66182
	Hs.654491	205805_s_at	ROR1	receptor tyrosine kinase-like orphan receptor 1	-1.65152
	Hs.151641	203835_at	LRRC32	leucine rich repeat containing 32	-1.65121
	Hs.106070	213182_x_at	CDKN1C	cyclin-dependent kinase inhibitor 1C (p57, Kip2)	-1.64929
	Hs.156727	223092_at	ANKH	ankylosis, progressive homolog (mouse)	-1.64759
	Hs.376206	220266_s_at	KLF4	Kruppel-like factor 4 (gut)	-1.64267
	Hs.282417	202990_at	PYGL	phosphorylase, glycogen, liver	-1.64005
	Hs.728967	226510_at	HEATR5A	HEAT repeat containing 5A	-1.63763
	Hs.406475	229554_at	LUM	lumican	-1.63531
	Hs.369201	238127_at	GAS6-AS1	GAS6 antisense RNA 1 (non-protein coding)	-1.63523
	Hs.436142	204201_s_at	PTPN13	protein tyrosine phosphatase, non-receptor type 13 (APO-1/CD95 (Fas)-associated phospha	-1.62875
	Hs.720935	1554609_at	LOC100287896	uncharacterized LOC100287896	-1.6265
	Hs.516173	206833_s_at	ACYP2	acylphosphatase 2, muscle type	-1.62571
	Hs.558009	213716_s_at	SECTM1	secreted and transmembrane 1	-1.62464
	Hs.144513	223557_s_at	TMEFF2	transmembrane protein with EGF-like and two follistatin-like domains 2	-1.62396
	Hs.102735	210829_s_at	SSBP2	single-stranded DNA binding protein 2	-1.62303
	Hs.731888	209826_at	EGFL8 /// PPT2 /// PPT2-EGFL8	EGF-like-domain, multiple 8 /// palmitoyl-protein thioesterase 2 /// PPT2-EGFL8 readthr	-1.6174
	Hs.477128	243864_at	CCDC80	coiled-coil domain containing 80	-1.61597
	Hs.40510	1554161_at	SLC25A27	solute carrier family 25, member 27	-1.61363
	Hs.467751	202478_at	TRIB2	tribbles homolog 2 (Drosophila)	-1.6132
	Hs.195710	227195_at	ZNF503	zinc finger protein 503	-1.61194
	Hs.202095	221950_at	EMX2	empty spiracles homeobox 2	-1.61178
	Hs.401954	224397_s_at	TMTC1	transmembrane and tetratricopeptide repeat containing 1	-1.61173
	Hs.654491	232060_at	ROR1	receptor tyrosine kinase-like orphan receptor 1	-1.61142
	Hs.464422	221019_s_at	COLEC12	collectin sub-family member 12	-1.6048
	Hs.484423	206377_at	FOXF2	forkhead box F2	-1.60471

	Hs.209151	225469_at	LYRM5	LYR motif containing 5	-1.60366
	Hs.655738	213386_at	TMEM246	transmembrane protein 246	-1.60332
	Hs.125056	228032_s_at	DENND1B	DENN/MADD domain containing 1B	-1.6031
	Hs.147765	205514_at	ZNF415	zinc finger protein 415	-1.60096
	Hs.60339	215743_at	NMT2	N-myristoyltransferase 2	-1.5994
	Hs.491172	221207_s_at	NBEA	neurobeachin	-1.59165
	Hs.192586	227522_at	CMBL	carboxymethylenebutenolidase homolog (Pseudomonas)	-1.59045
	Hs.709200	208134_x_at	PSG2	pregnancy specific beta-1-glycoprotein 2	-1.58979
	Hs.288954	226464_at	C3orf58	chromosome 3 open reading frame 58	-1.58617
	Hs.631988	208779_x_at	DDR1 /// MIR4640	discoidin domain receptor tyrosine kinase 1 /// microRNA 4640	-1.58323
	Hs.440955	213416_at	ITGA4	integrin, alpha 4 (antigen CD49D, alpha 4 subunit of VLA-4 receptor)	-1.58166
	Hs.128199	214293_at	sept-11	septin 11	-1.57886
	Hs.632832	230075_at	RAB39B	RAB39B, member RAS oncogene family	-1.57502
	Hs.87734	235365_at	DFNB59	deafness, autosomal recessive 59	-1.57315
	Hs.156352	236325_at	KIAA1377	KIAA1377	-1.57148
	Hs.434255	218613_at	PSD3	pleckstrin and Sec7 domain containing 3	-1.57059
	Hs.405156	212230_at	PPAP2B	phosphatidic acid phosphatase type 2B	-1.56807
	Hs.118127	205132_at	ACTC1	actin, alpha, cardiac muscle 1	-1.56772
	Hs.288741	227955_s_at	EFNA5	ephrin-A5	-1.56756
	Hs.173716	233868_x_at	ADAM33	ADAM metallopeptidase domain 33	-1.56596
	Hs.716678	244050_at	PTPLAD2	protein tyrosine phosphatase-like A domain containing 2	-1.56562
	Hs.650158	225295_at	SLC39A10	solute carrier family 39 (zinc transporter), member 10	-1.565
	Hs.471130	226431_at	FAM117B	family with sequence similarity 117, member B	-1.5645
	Hs.601314	203159_at	GLS	glutaminase	-1.56044
	Hs.653262	213001_at	ANGPTL2	angiopoietin-like 2	-1.55631
	Hs.445030	225202_at	RHOBTB3	Rho-related BTB domain containing 3	-1.55339
	Hs.152385	225327_at	FAM214A	family with sequence similarity 214, member A	-1.55314
	Hs.266175	225626_at	PAG1	phosphoprotein associated with glycosphingolipid microdomains 1	-1.55006
	Hs.596096	227568_at	HECTD2	HECT domain containing E3 ubiquitin protein ligase 2	-1.54991
	Hs.300701	214023_x_at	TUBB2B	tubulin, beta 2B class IIb	-1.54981
	Hs.21590	221595_at	RBM48	RNA binding motif protein 48	-1.54756
	Hs.507755	205399_at	DCLK1	doublecortin-like kinase 1	-1.54651
	Hs.221941	222453_at	CYBRD1	cytochrome b reductase 1	-1.54538
	Hs.503831	1556886_a_at	LAYN	Layilin	-1.54388
	Hs.719958	205200_at	CLEC3B /// EXOSC7	C-type lectin domain family 3, member B /// exosome component 7	-1.54206
	Hs.374774	229308_at	ANKRD29	ankyrin repeat domain 29	-1.54202
	Hs.400698	229603_at	BBS12	Bardet-Biedl syndrome 12	-1.54198
	Hs.156316	209335_at	DCN	decorin	-1.53969
	Hs.288741	214036_at	EFNA5	ephrin-A5	-1.53846
	Hs.437040	40524_at	PTPN21	protein tyrosine phosphatase, non-receptor type 21	-1.53732
	Hs.161000	230141_at	ARID4A	AT rich interactive domain 4A (RBP1-like)	-1.53435
	Hs.483993	218736_s_at	PALMD	palmdelphin	-1.53354
	Hs.164162	227148_at	PLEKHH2	pleckstrin homology domain containing, family H (with MyTH4 domain) member 2	-1.53298
	Hs.726427	235010_at	LOC729013	uncharacterized LOC729013	-1.52542
	Hs.353001	221900_at	COL8A2	collagen, type VIII, alpha 2	-1.52525
	Hs.197043	214180_at	MAN1C1	mannosidase, alpha, class 1C, member 1	-1.51928
	Hs.641481	231838_at	PABPC1L	poly(A) binding protein, cytoplasmic 1-like	-1.51787
	Hs.458312	227839_at	MBD5	methyl-CpG binding domain protein 5	-1.51673
	Hs.203965	215286_s_at	PHTF2	putative homeodomain transcription factor 2	-1.51481
	Hs.437609	226757_at	IFIT2	interferon-induced protein with tetratricopeptide repeats 2	-1.51381
	Hs.156352	235956_at	KIAA1377	KIAA1377	-1.51204
	Hs.425769	226019_at	OMA1	OMA1 zinc metallopeptidase homolog (S. cerevisiae)	-1.5117

	Hs.465433	234977_at	ZADH2	zinc binding alcohol dehydrogenase domain containing 2	-1.5115
	Hs.152944	210102_at	VWA5A	von Willebrand factor A domain containing 5A	-1.50928
	Hs.371609	227221_at	ZMAT3	zinc finger, matrin-type 3	-1.50826
	Hs.484195	225956_at	CREBRF	CREB3 regulatory factor	-1.5054
	Hs.554182	230228_at	SSC5D	scavenger receptor cysteine rich domain containing (5 domains)	-1.50466
	Hs.653262	213004_at	ANGPTL2	angiopoietin-like 2	-1.50419
	Hs.40510	230624_at	SLC25A27	solute carrier family 25, member 27	-1.50373
	Hs.740514	219032_x_at	OPN3	opsin 3	-1.50022
	Hs.732223	228702_at	FLJ43663	uncharacterized LOC378805	-1.49913
	Hs.140617	1568658_at	C2orf74 /// KIAA1841	chromosome 2 open reading frame 74 /// KIAA1841	-1.49909
	Hs.231883	241739_at	OGFOD1	2-oxoglutarate and iron-dependent oxygenase domain containing 1	-1.49869
	Hs.740551	235011_at	MAP3K2	mitogen-activated protein kinase kinase kinase 2	-1.49488
	Hs.149168	227812_at	TNFRSF19	tumor necrosis factor receptor superfamily, member 19	-1.49402
	Hs.58367	204984_at	GPC4	glypican 4	-1.49171
	Hs.509264	217906_at	KLHDC2	kelch domain containing 2	-1.49043
	Hs.435458	227478_at	SETBP1	SET binding protein 1	-1.49007
	Hs.293798	226113_at	ZNF436	zinc finger protein 436	-1.4893
	Hs.102308	205304_s_at	KCNJ8	potassium inwardly-rectifying channel, subfamily J, member 8	-1.48468
	Hs.700632	227548_at	ORMDL1	ORM1-like 1 (S. cerevisiae)	-1.48209
	Hs.98328	224463_s_at	C11orf70	chromosome 11 open reading frame 70	-1.48204
	Hs.740483	226119_at	PCMTD1	protein-L-isoaspartate (D-aspartate) O-methyltransferase domain containing 1	-1.47825
	Hs.190544	1552275_s_at	PXK	PX domain containing serine/threonine kinase	-1.47754
	Hs.577775	228574_at	TMTC2	transmembrane and tetratricopeptide repeat containing 2	-1.47454
	Hs.460923	1554052_at	CNOT1	CCR4-NOT transcription complex, subunit 1	-1.47401
	Hs.397729	221750_at	HMGCS1	3-hydroxy-3-methylglutaryl-CoA synthase 1 (soluble)	-1.46928
	---	228642_at	HOTAIRM1	HOXA transcript antisense RNA, myeloid-specific 1 (non-protein coding)	-1.46891
	Hs.293970	221589_s_at	ALDH6A1	aldehyde dehydrogenase 6 family, member A1	-1.46763
	Hs.288348	220911_s_at	NYNRIN	NYN domain and retroviral integrase containing	-1.46711
	Hs.202521	239297_at	KIAA1456	KIAA1456	-1.46579
	Hs.631957	231867_at	ODZ2	odz, odd Oz/ten-m homolog 2 (Drosophila)	-1.46458
	Hs.610508	235518_at	SLC8A1	solute carrier family 8 (sodium/calcium exchanger), member 1	-1.46411
	Hs.484738	223130_s_at	MYLIP	myosin regulatory light chain interacting protein	-1.46323
	Hs.530904	207030_s_at	CSRP2	cysteine and glycine-rich protein 2	-1.46156
	Hs.149103	1554030_at	ARSB	arylsulfatase B	-1.46151
	Hs.591085	204917_s_at	MLLT3	myeloid/lymphoid or mixed-lineage leukemia (trithorax homolog, Drosophila); translocate	-1.46142
	Hs.731767	202364_at	MXI1	MAX interactor 1	-1.4613
	Hs.15114	209885_at	RHOD	ras homolog family member D	-1.45964
	Hs.510989	207480_s_at	MEIS2	Meis homeobox 2	-1.45758
	Hs.407015	242138_at	DLX1	distal-less homeobox 1	-1.45634
	Hs.128576	230747_s_at	TTC39C	tetratricopeptide repeat domain 39C	-1.4513
	Hs.534052	201531_at	ZFP36	zinc finger protein 36, C3H type, homolog (mouse)	-1.45071
	Hs.710370	230435_at	FAM228B	family with sequence similarity 228, member B	-1.448
	Hs.102336	47069_at	PRR5	proline rich 5 (renal)	-1.44674
	Hs.23871	226311_at	ADAMTS2	ADAM metallopeptidase with thrombospondin type 1 motif, 2	-1.44544
	Hs.4276	213709_at	BHLHB9	basic helix-loop-helix domain containing, class B, 9	-1.44366
	Hs.130014	203799_at	CD302 /// LY75-CD302	CD302 molecule /// LY75-CD302 readthrough	-1.44328
	Hs.369232	225855_at	EPB41L5	erythrocyte membrane protein band 4.1 like 5	-1.44314
	Hs.534847	214428_x_at	C4A /// C4B /// LOC100293534	complement component 4A (Rodgers blood group) /// complement component 4B (Chido blood	-1.44308
	Hs.370725	208158_s_at	OSBPL1A	oxysterol binding protein-like 1A	-1.44303
	Hs.262480	229071_at	C17orf100	chromosome 17 open reading frame 100	-1.44291
	Hs.497626	227032_at	PLXNA2	plexin A2	-1.44284

	Hs.522019	239909_at	ADAMTSL1	ADAMTS-like 1	-1.44202
	Hs.726435	210129_s_at	ARPC4-TTLL3 /// TTLL3	ARPC4-TTLL3 readthrough /// tubulin tyrosine ligase-like family, member 3	-1.43854
	Hs.303208	219749_at	SH2D4A	SH2 domain containing 4A	-1.43806
	Hs.286073	204566_at	PPM1D	protein phosphatase, Mg2+/Mn2+ dependent, 1D	-1.43695
	Hs.370605	1554256_a_at	PCNXL2	pecanex-like 2 (Drosophila)	-1.43613
	Hs.235935	214321_at	NOV	nephroblastoma overexpressed	-1.43607
	Hs.650585	211651_s_at	LAMB1	laminin, beta 1	-1.43533
	Hs.522019	229585_at	ADAMTSL1	ADAMTS-like 1	-1.43464
	Hs.17546	231530_s_at	C11orf1	chromosome 11 open reading frame 1	-1.43439
	Hs.330073	203288_at	KIAA0355	KIAA0355	-1.43404
	Hs.153863	207069_s_at	SMAD6	SMAD family member 6	-1.43392
	Hs.484950	215071_s_at	HIST1H2AC	histone cluster 1, H2ac	-1.43323
	Hs.274415	225579_at	PQLC3	PQ loop repeat containing 3	-1.43184
	Hs.479403	220260_at	TBC1D19	TBC1 domain family, member 19	-1.43091
	Hs.475353	218574_s_at	LMCD1	LIM and cysteine-rich domains 1	-1.43085
	Hs.122927	226425_at	CLIP4	CAP-GLY domain containing linker protein family, member 4	-1.4306
	Hs.522484	205591_at	OLFM1	olfactomedin 1	-1.42957
	Hs.221436	235570_at	RBMS3	RNA binding motif, single stranded interacting protein 3	-1.42844
	Hs.573143	229487_at	EBF1	early B-cell factor 1	-1.42731
	Hs.659762	229685_at	LOC100134937	uncharacterized LOC100134937	-1.4256
	Hs.631650	227070_at	GLT8D2	glycosyltransferase 8 domain containing 2	-1.42474
	---	229190_at	LOC100507376	uncharacterized LOC100507376	-1.42404
	Hs.732252	218816_at	LRRC1	leucine rich repeat containing 1	-1.42343
	Hs.444959	205364_at	ACOX2	acyl-CoA oxidase 2, branched chain	-1.42128
	Hs.309288	202157_s_at	CELF2	CUGBP, Elav-like family member 2	-1.42067
	Hs.401954	226322_at	TMTC1	transmembrane and tetratricopeptide repeat containing 1	-1.4176
	Hs.731687	221895_at	MOSPD2	motile sperm domain containing 2	-1.41756
	Hs.1501	212154_at	SDC2	syndecan 2	-1.41635
	Hs.524250	208868_s_at	GABARAPL1	GABA(A) receptor-associated protein like 1	-1.41611
	Hs.476365	201339_s_at	SCP2	sterol carrier protein 2	-1.41236
	Hs.425769	226020_s_at	DAB1 /// OMA1	disabled homolog 1 (Drosophila) /// OMA1 zinc metallopeptidase homolog (S. cerevisiae)	-1.41067
	Hs.631650	221447_s_at	GLT8D2	glycosyltransferase 8 domain containing 2	-1.41044
	Hs.525324	211792_s_at	CDKN2C	cyclin-dependent kinase inhibitor 2C (p18, inhibits CDK4)	-1.41035
	Hs.478067	218729_at	LXN	latexin	-1.4074
	Hs.654742	232080_at	HECW2	HECT, C2 and WW domain containing E3 ubiquitin protein ligase 2	-1.40694
	Hs.75652	205752_s_at	GSTM5	glutathione S-transferase mu 5	-1.40616
	Hs.484068	204547_at	RAB40B	RAB40B, member RAS oncogene family	-1.40589
	Hs.740530	45714_at	HCFC1R1	host cell factor C1 regulator 1 (XPO1 dependent)	-1.4058
	Hs.128576	1552307_a_at	TTC39C	tetratricopeptide repeat domain 39C	-1.40575
	Hs.535394	230869_at	FAM155A	family with sequence similarity 155, member A	-1.40561
	Hs.7966	203870_at	USP46	ubiquitin specific peptidase 46	-1.40553
	Hs.55967	210135_s_at	SHOX2	short stature homeobox 2	-1.40506
	Hs.533336	203304_at	BAMBI	BMP and activin membrane-bound inhibitor homolog (Xenopus laevis)	-1.40481
	Hs.292986	226126_at	TBCK	TBC1 domain containing kinase	-1.40437
	Hs.657617	230174_at	LYPLAL1	lysophospholipase-like 1	-1.4032
	Hs.591968	218665_at	FZD4	frizzled family receptor 4	-1.40212
	Hs.501012	201753_s_at	ADD3	adducin 3 (gamma)	-1.40133
	Hs.517717	203408_s_at	SATB1	SATB homeobox 1	-1.40059
	Hs.147880	227058_at	C13orf33	chromosome 13 open reading frame 33	-1.40028
	Hs.654713	226657_at	C17orf103	chromosome 17 open reading frame 103	-1.39931
	Hs.190341	205618_at	PRRG1	proline rich Gla (G-carboxyglutamic acid) 1	-1.39895

	Hs.388613	225270_at	NEO1	neogenin 1	-1.39808
	Hs.171189	202820_at	AHR	aryl hydrocarbon receptor	-1.39769
	Hs.605775	226493_at	KCTD18	potassium channel tetramerisation domain containing 18	-1.3966
	Hs.519162	201236_s_at	BTG2	BTG family, member 2	-1.39534
	Hs.532824	202501_at	MAPRE2	microtubule-associated protein, RP/EB family, member 2	-1.39457
	Hs.731687	64883_at	MOSPD2	motile sperm domain containing 2	-1.39404
	Hs.40794	230475_at	C15orf59	chromosome 15 open reading frame 59	-1.39305
	Hs.507991	230135_at	HHIP	hedgehog interacting protein	-1.39284
	Hs.465433	227049_at	ZADH2	zinc binding alcohol dehydrogenase domain containing 2	-1.3912
	Hs.723178	209894_at	LEPR	leptin receptor	-1.39037
	Hs.499886	202053_s_at	ALDH3A2	aldehyde dehydrogenase 3 family, member A2	-1.38998
	Hs.380089	204718_at	EPHB6	EPH receptor B6	-1.38993
	Hs.333786	221573_at	C7orf25 /// PSMA2	chromosome 7 open reading frame 25 /// proteasome (prosome, macropain) subunit, alpha t	-1.38985
	Hs.124776	214803_at	CDH6	cadherin 6, type 2, K-cadherin (fetal kidney)	-1.38974
	Hs.522074	208763_s_at	TSC22D3	TSC22 domain family, member 3	-1.38961
	Hs.632629	224061_at	INMT	indolethylamine N-methyltransferase	-1.38951
	Hs.723297	226366_at	SHPRH	SNF2 histone linker PHD RING helicase, E3 ubiquitin protein ligase	-1.38891
	Hs.171311	235666_at	ITGA8	integrin, alpha 8	-1.3888
	Hs.435458	205933_at	SETBP1	SET binding protein 1	-1.38823
	Hs.131226	221478_at	BNIP3L	BCL2/adenovirus E1B 19kDa interacting protein 3-like	-1.38709
	Hs.613170	200632_s_at	NDRG1	N-myc downstream regulated 1	-1.38607
	Hs.619593	204215_at	C7orf23	chromosome 7 open reading frame 23	-1.38393
	Hs.266616	229817_at	ZNF608	zinc finger protein 608	-1.38317
	Hs.720221	226069_at	PRICKLE1	prickle homolog 1 (Drosophila)	-1.38127
	Hs.368944	225800_at	JAZF1	JAZF zinc finger 1	-1.37949
	Hs.271667	212653_s_at	EHBP1	EH domain binding protein 1	-1.37868
	Hs.371240	227529_s_at	AKAP12	A kinase (PRKA) anchor protein 12	-1.37777
	Hs.740456	202769_at	CCNG2	cyclin G2	-1.37658
	---	229354_at	AHRR	aryl-hydrocarbon receptor repressor	-1.37617
	Hs.709192	210195_s_at	PSG1	pregnancy specific beta-1-glycoprotein 1	-1.37561
	Hs.203830	231738_at	PCDHB7	protocadherin beta 7	-1.37518
	Hs.154163	221564_at	PRMT2	protein arginine methyltransferase 2	-1.37467
	Hs.465087	204790_at	SMAD7	SMAD family member 7	-1.37138
	Hs.127286	235205_at	OXR1	oxidation resistance 1	-1.37096
	---	229352_at	SPESP1	sperm equatorial segment protein 1	-1.3709
	Hs.655832	225806_at	AJUBA	ajuba LIM protein	-1.36986
	Hs.375092	1564383_s_at	FLJ35934	FLJ35934	-1.36874
	Hs.232021	208070_s_at	REV3L	REV3-like, polymerase (DNA directed), zeta, catalytic subunit	-1.36862
	Hs.603510	229092_at	NR2F2	nuclear receptor subfamily 2, group F, member 2	-1.36711
	Hs.731383	218309_at	CAMK2N1	calcium/calmodulin-dependent protein kinase II inhibitor 1	-1.36631
	Hs.102735	203787_at	SSBP2	single-stranded DNA binding protein 2	-1.3642
	Hs.600545	229674_at	SERTAD4	SERTA domain containing 4	-1.36348
	Hs.190544	225796_at	PXK	PX domain containing serine/threonine kinase	-1.3634
	Hs.643910	201417_at	SOX4	SRY (sex determining region Y)-box 4	-1.36294
	Hs.486357	213624_at	SMPDL3A	sphingomyelin phosphodiesterase, acid-like 3A	-1.36077
	Hs.61884	229086_at	C1orf213	chromosome 1 open reading frame 213	-1.36025
	Hs.107149	220992_s_at	TRMT1L	tRNA methyltransferase 1 homolog (S. cerevisiae)-like	-1.35951
	Hs.104305	218380_at	LOC728392	uncharacterized LOC728392	-1.35869
	Hs.507669	204072_s_at	FRY	furry homolog (Drosophila)	-1.35834
	Hs.731773	207621_s_at	PEMT	phosphatidylethanolamine N-methyltransferase	-1.35809
	Hs.654449	230108_at	ERCC6	excision repair cross-complementing rodent repair deficiency, complementation group 6	-1.35774
	Hs.485557	235405_at	GSTA4	glutathione S-transferase alpha 4	-1.35726

	Hs.79101	208796_s_at	CCNG1	cyclin G1	-1.3533
	Hs.379018	226157_at	TFDP2	transcription factor Dp-2 (E2F dimerization partner 2)	-1.3531
	Hs.482605	226352_at	JMY	junction mediating and regulatory protein, p53 cofactor	-1.35161
	Hs.515490	228228_at	DACT3	dapper, antagonist of beta-catenin, homolog 3 (Xenopus laevis)	-1.35155
	Hs.660396	244246_at	MIPOL1	mirror-image polydactyly 1	-1.35057
	Hs.29173	51192_at	SSH3	slingshot homolog 3 (Drosophila)	-1.35045
	Hs.1570	205579_at	HRH1	histamine receptor H1	-1.34842
	Hs.592184	210849_s_at	VPS41	vacuolar protein sorting 41 homolog (S. cerevisiae)	-1.3482
	Hs.654415	204830_x_at	PSG5	pregnancy specific beta-1-glycoprotein 5	-1.34767
	Hs.173716	232570_s_at	ADAM33	ADAM metallopeptidase domain 33	-1.34655
	Hs.593995	216037_x_at	TCF7L2	transcription factor 7-like 2 (T-cell specific, HMG-box)	-1.34506
	Hs.522412	204480_s_at	C9orf16	chromosome 9 open reading frame 16	-1.34428
	Hs.459534	240771_at	C1orf101	chromosome 1 open reading frame 101	-1.34322
	Hs.511991	235410_at	NPHP3	nephronophthisis 3 (adolescent)	-1.34178
	Hs.590944	206648_at	ZNF571	zinc finger protein 571	-1.34095
	Hs.655602	226334_s_at	AHSA2	AHA1, activator of heat shock 90kDa protein ATPase homolog 2 (yeast)	-1.34041
	Hs.435938	217920_at	MAN1A2	mannosidase, alpha, class 1A, member 2	-1.33984
	Hs.356247	225421_at	PM20D2	peptidase M20 domain containing 2	-1.33837
	Hs.253726	228569_at	PAPOLA	poly(A) polymerase alpha	-1.33824
	Hs.632595	236632_at	HHIP-AS1	HHIP antisense RNA 1 (non-protein coding)	-1.33809
	Hs.652324	226800_at	EFCAB7	EF-hand calcium binding domain 7	-1.33606
	Hs.592014	211458_s_at	GABARAPL1 /// GABARAPL3	GABA(A) receptor-associated protein like 1 /// GABA(A) receptors associated protein lik	-1.33426
	Hs.154163	228722_at	PRMT2	protein arginine methyltransferase 2	-1.33419
	Hs.567725	225162_at	SH3D19	SH3 domain containing 19	-1.33333
	Hs.370725	209485_s_at	OSBPL1A	oxysterol binding protein-like 1A	-1.33328
	Hs.526665	224492_s_at	ZNF627	zinc finger protein 627	-1.33326
	Hs.527412	213702_x_at	ASAH1	N-acylsphingosine amidohydrolase (acid ceramidase) 1	-1.33257
	Hs.518662	217967_s_at	FAM129A	family with sequence similarity 129, member A	-1.33228
	Hs.288912	1552426_a_at	TM2D3	TM2 domain containing 3	-1.3321
	Hs.709461	206857_s_at	FKBP1B	FK506 binding protein 1B, 12.6 kDa	-1.33209
	Hs.401798	212736_at	C16orf45	chromosome 16 open reading frame 45	-1.33207
	Hs.594287	235124_at	LOC645212	uncharacterized LOC645212	-1.33202
	Hs.573143	227646_at	EBF1	early B-cell factor 1	-1.33172
	Hs.653654	225927_at	MAP3K1	mitogen-activated protein kinase kinase kinase 1, E3 ubiquitin protein ligase	-1.33168
	Hs.24587	204400_at	EFS	embryonal Fyn-associated substrate	-1.33137
	Hs.609773	223125_s_at	C1orf21	chromosome 1 open reading frame 21	-1.3312
	Hs.525093	224799_at	NDFIP2	Nedd4 family interacting protein 2	-1.33062
	Hs.740376	212231_at	FBXO21	F-box protein 21	-1.33061
	Hs.512842	213765_at	MFAP5	microfibrillar associated protein 5	-1.33021
	Hs.507680	214748_at	N4BP2L2	NEDD4 binding protein 2-like 2	-1.32907
	Hs.696231	209147_s_at	PPAP2A	phosphatidic acid phosphatase type 2A	-1.329
	Hs.285051	214151_s_at	CCPG1 /// DYX1C1-CCPG1	cell cycle progression 1 /// DYX1C1-CCPG1 readthrough (non-protein coding)	-1.32856
	Hs.368325	211106_at	SUPT3H	suppressor of Ty 3 homolog (S. cerevisiae)	-1.32833
	Hs.535801	226125_at	LOC100288152	uncharacterized LOC100288152	-1.32825
	Hs.477475	221906_at	TXNRD3 /// TXNRD3NB	thioredoxin reductase 3 /// thioredoxin reductase 3 neighbor	-1.32803
	Hs.12907	209975_at	CYP2E1	cytochrome P450, family 2, subfamily E, polypeptide 1	-1.32791
	Hs.500409	200946_x_at	GLUD1	glutamate dehydrogenase 1	-1.32782
	Hs.49774	203329_at	PTPRM	protein tyrosine phosphatase, receptor type, M	-1.32661
	Hs.25391	228312_at	PI16	peptidase inhibitor 16	-1.32369
	Hs.535711	202080_s_at	TRAK1	trafficking protein, kinesin binding 1	-1.32254

	Hs.95260	203420_at	FAM8A1	family with sequence similarity 8, member A1	-1.32229
	Hs.434253	212848_s_at	C9orf3 /// LOC100507319	chromosome 9 open reading frame 3 /// uncharacterized LOC100507319	-1.32107
	Hs.28896	232087_at	CXorf23	chromosome X open reading frame 23	-1.32071
	Hs.26837	212436_at	TRIM33	tripartite motif containing 33	-1.32064
	Hs.13351	202020_s_at	LANCL1	LanC lantibiotic synthetase component C-like 1 (bacterial)	-1.32043
	Hs.591474	231773_at	ANGPTL1	angiopoietin-like 1	-1.31961
	Hs.356399	222605_at	RCOR3	REST corepressor 3	-1.31881
	Hs.405692	218628_at	CCDC53	coiled-coil domain containing 53	-1.3175
	Hs.524804	1556042_s_at	LOC338799	uncharacterized LOC338799	-1.31664
	Hs.461647	212056_at	KIAA0182	KIAA0182	-1.31552
	---	78383_at	LOC100129250	uncharacterized LOC100129250	-1.31471
	Hs.731605	204565_at	ACOT13	acyl-CoA thioesterase 13	-1.31413
	Hs.434255	203355_s_at	PSD3	pleckstrin and Sec7 domain containing 3	-1.31395
	Hs.643130	226231_at	PAWR	PRKC, apoptosis, WT1, regulator	-1.31288
	Hs.657131	228247_at	LOC283788	FSHD region gene 1 pseudogene	-1.31234
	Hs.84928	218127_at	NFYB	nuclear transcription factor Y, beta	-1.31209
	Hs.76224	201842_s_at	EFEMP1	EGF containing fibulin-like extracellular matrix protein 1	-1.31162
	Hs.410378	231130_at	FKBP7	FK506 binding protein 7	-1.31139
	Hs.239631	227089_at	COG5	component of oligomeric golgi complex 5	-1.31106
	Hs.660115	219729_at	PRRX2	paired related homeobox 2	-1.31042
	Hs.423163	203306_s_at	SLC35A1	solute carrier family 35 (CMP-sialic acid transporter), member A1	-1.31032
	Hs.435369	201540_at	FHL1	four and a half LIM domains 1	-1.31031
	---	236656_s_at	LOC100288911	uncharacterized LOC100288911	-1.30923
	Hs.162032	209102_s_at	HBP1	HMG-box transcription factor 1	-1.30917
	Hs.437241	213238_at	ATP10D	ATPase, class V, type 10D	-1.30805
	Hs.306764	213005_s_at	KANK1	KN motif and ankyrin repeat domains 1	-1.30789
	Hs.710546	224975_at	NFIA	nuclear factor I/A	-1.30699
	Hs.465433	227978_s_at	ZADH2	zinc binding alcohol dehydrogenase domain containing 2	-1.30692
	---	219880_at	LOC100507619	uncharacterized LOC100507619	-1.30654
	Hs.656199	206091_at	MATN3	matrilin 3	-1.30621
	Hs.465433	1554239_s_at	ZADH2	zinc binding alcohol dehydrogenase domain containing 2	-1.30613
	Hs.500466	204053_x_at	PTEN	phosphatase and tensin homolog	-1.30535
	Hs.305971	221024_s_at	SLC2A10	solute carrier family 2 (facilitated glucose transporter), member 10	-1.30464
	Hs.509067	202273_at	PDGFRB	platelet-derived growth factor receptor, beta polypeptide	-1.30458
	Hs.477475	59631_at	TXNRD3 /// TXNRD3NB	thioredoxin reductase 3 /// thioredoxin reductase 3 neighbor	-1.30376
	Hs.356061	208786_s_at	MAP1LC3B	microtubule-associated protein 1 light chain 3 beta	-1.30337
	Hs.20848	226668_at	WDSUB1	WD repeat, sterile alpha motif and U-box domain containing 1	-1.30193
	Hs.501289	226680_at	IKZF5	IKAROS family zinc finger 5 (Pegasus)	-1.3019
	Hs.93842	226390_at	STARD4	StAR-related lipid transfer (START) domain containing 4	-1.30101
	Hs.659311	213626_at	CBR4	carbonyl reductase 4	-1.30087
	Hs.114948	206315_at	CRLF1	cytokine receptor-like factor 1	-1.29986
	Hs.128576	238480_at	TTC39C	tetratricopeptide repeat domain 39C	-1.29948
	Hs.444668	223594_at	TMEM117	transmembrane protein 117	-1.29799
	Hs.709257	239442_at	CEP68	centrosomal protein 68kDa	-1.29737
	Hs.336768	209459_s_at	ABAT	4-aminobutyrate aminotransferase	-1.29726
	Hs.632238	202781_s_at	INPP5K	inositol polyphosphate-5-phosphatase K	-1.29714
	Hs.514950	218217_at	SCPEP1	serine carboxypeptidase 1	-1.29709
	Hs.514199	208626_s_at	VAT1	vesicle amine transport protein 1 homolog (T. californica)	-1.29701
	Hs.511504	208986_at	TCF12	transcription factor 12	-1.29658
	---	228284_at	TLE1	transducin-like enhancer of split 1 (E(sp1) homolog, Drosophila)	-1.29612

	Hs.513463	209230_s_at	NUPR1	nuclear protein, transcriptional regulator, 1	-1.2959
	Hs.409210	207232_s_at	DZIP3	DAZ interacting protein 3, zinc finger	-1.29549
	Hs.576320	224990_at	C4orf34	chromosome 4 open reading frame 34	-1.29501
	Hs.202522	1558692_at	C1orf85	chromosome 1 open reading frame 85	-1.29501
	Hs.525299	224484_s_at	BRMS1L	breast cancer metastasis-suppressor 1-like	-1.29443
	Hs.128199	201307_at	sept-11	septin 11	-1.29433
	Hs.553221	226759_at	IKZF4	IKAROS family zinc finger 4 (Eos)	-1.29411
	Hs.567502	225274_at	PCYOX1	prenylcysteine oxidase 1	-1.29357
	Hs.309489	227001_at	NIPAL2	NIPA-like domain containing 2	-1.29356
	Hs.241545	221565_s_at	CALHM2	calcium homeostasis modulator 2	-1.29308
	Hs.534322	208306_x_at	HLA-DRB1 /// LOC100507709 /// LOC100507714	major histocompatibility complex, class II, DR beta 1 /// HLA class II histocompatibili	-1.29298
	Hs.21160	204058_at	ME1	malic enzyme 1, NADP(+)-dependent, cytosolic	-1.29277
	---	236640_at	LOC100507165	uncharacterized LOC100507165	-1.29249
	Hs.108614	212308_at	CLASP2	cytoplasmic linker associated protein 2	-1.29209
	Hs.522484	213131_at	OLFM1	olfactomedin 1	-1.29164
	Hs.14745	224647_at	CCNY	cyclin Y	-1.29064
	Hs.471234	227280_s_at	CCNYL1	cyclin Y-like 1	-1.28994
	Hs.222055	1554010_at	NDST1	N-deacetylase/N-sulfotransferase (heparan glucosaminyl) 1	-1.28989
	Hs.198158	219543_at	PBLD	phenazine biosynthesis-like protein domain containing	-1.2891
	Hs.585006	1552698_at	TUBA3FP	tubulin, alpha 3f, pseudogene	-1.28905
	Hs.102914	202214_s_at	CUL4B	cullin 4B	-1.28882
	Hs.43728	213170_at	GPX7	glutathione peroxidase 7	-1.28804
	Hs.596314	209106_at	NCOA1	nuclear receptor coactivator 1	-1.28782
	Hs.336768	209460_at	ABAT	4-aminobutyrate aminotransferase	-1.28645
	Hs.118166	228551_at	DENND5B	DENN/MADD domain containing 5B	-1.28608
	Hs.513871	225804_at	CYB5D2	cytochrome b5 domain containing 2	-1.28561
	Hs.434993	227859_at	DNAJC27	DnaJ (Hsp40) homolog, subfamily C, member 27	-1.28548
	Hs.262960	224220_x_at	TRPC4	transient receptor potential cation channel, subfamily C, member 4	-1.28512
	Hs.526630	226245_at	KCTD1	potassium channel tetramerisation domain containing 1	-1.28507
	Hs.162989	227193_at	SLC30A4	solute carrier family 30 (zinc transporter), member 4	-1.28414
	Hs.241545	57715_at	CALHM2	calcium homeostasis modulator 2	-1.28413
	Hs.725347	235173_at	LOC401093	uncharacterized LOC401093	-1.28363
	Hs.520287	212179_at	PNISR	PNN-interacting serine/arginine-rich protein	-1.28329
	Hs.501200	204319_s_at	RGS10	regulator of G-protein signaling 10	-1.2822
	Hs.567828	226721_at	DPY19L4	dpy-19-like 4 (C. elegans)	-1.28198
	Hs.648565	222103_at	ATF1	activating transcription factor 1	-1.28189
	Hs.187635	228291_s_at	PLK1S1	polo-like kinase 1 substrate 1	-1.28028
	Hs.8715	223157_at	NOA1	nitric oxide associated 1	-1.28012
	Hs.643588	207558_s_at	PITX2	paired-like homeodomain 2	-1.28004
	Hs.153026	209306_s_at	SWAP70	SWAP switching B-cell complex 70kDa subunit	-1.27995
	Hs.333738	223227_at	BBS2	Bardet-Biedl syndrome 2	-1.27879
	Hs.522863	223646_s_at	TXLNG2P	taxilin gamma 2, pseudogene	-1.2774
	Hs.518200	223220_s_at	PARP9	poly (ADP-ribose) polymerase family, member 9	-1.2766
	Hs.50868	204981_at	SLC22A18	solute carrier family 22, member 18	-1.27635
	Hs.126558	235151_at	LOC283357	uncharacterized LOC283357	-1.27632
	Hs.435535	218149_s_at	ZNF395	zinc finger protein 395	-1.27611
	Hs.381167	213572_s_at	SERPINB1	serpin peptidase inhibitor, clade B (ovalbumin), member 1	-1.27522
	Hs.414809	204568_at	ATG14	autophagy related 14	-1.27471
	Hs.740366	217971_at	LAMTOR3	late endosomal/lysosomal adaptor, MAPK and MTOR activator 3	-1.27455
	---	205158_at	RNASE4	ribonuclease, RNase A family, 4	-1.27428

	Hs.493716	217492_s_at	PTEN /// PTENP1	phosphatase and tensin homolog /// phosphatase and tensin homolog pseudogene 1	-1.2742
	Hs.166017	207233_s_at	MITF	microphthalmia-associated transcription factor	-1.27407
	Hs.11614	222890_at	CCDC113	coiled-coil domain containing 113	-1.27363
	Hs.5741	219079_at	CYB5R4	cytochrome b5 reductase 4	-1.27343
	Hs.49774	1555579_s_at	PTPRM	protein tyrosine phosphatase, receptor type, M	-1.27331
	Hs.536663	201124_at	ITGB5	integrin, beta 5	-1.27182
	Hs.11637	226995_at	LOC642852	uncharacterized LOC642852	-1.27177
	Hs.684904	202275_at	G6PD	glucose-6-phosphate dehydrogenase	-1.27116
	Hs.593995	212761_at	TCF7L2	transcription factor 7-like 2 (T-cell specific, HMG-box)	-1.27111
	Hs.517830	214116_at	BTD	biotinidase	-1.27092
	---	213397_x_at	RNASE4	ribonuclease, RNase A family, 4	-1.27023
	Hs.41502	219563_at	LINC00341	long intergenic non-protein coding RNA 341	-1.26975
	Hs.616962	221577_x_at	GDF15	growth differentiation factor 15	-1.26948
	Hs.88297	226525_at	STK17B	serine/threonine kinase 17b	-1.26947
	Hs.157378	212798_s_at	ANKMY2	ankyrin repeat and MYND domain containing 2	-1.26943
	Hs.496267	202105_at	IGBP1	immunoglobulin (CD79A) binding protein 1	-1.26895
	Hs.387207	214492_at	SGCD	sarcoglycan, delta (35kDa dystrophin-associated glycoprotein)	-1.26795
	Hs.656313	227007_at	TMCO4	transmembrane and coiled-coil domains 4	-1.26666
	Hs.518545	229285_at	RNASEL	ribonuclease L (2',5'-oligoisoadenylate synthetase-dependent)	-1.26652
	Hs.546430	244834_at	RSG1	REM2 and RAB-like small GTPase 1	-1.26552
	Hs.401929	203912_s_at	DNASE1L1	deoxyribonuclease I-like 1	-1.26537
	Hs.12967	209447_at	SYNE1	spectrin repeat containing, nuclear envelope 1	-1.26422
	Hs.720151	210425_x_at	GOLGA8A /// GOLGA8B /// LOC100508892	golgin A8 family, member A /// golgin A8 family, member B /// uncharacterized LOC100508	-1.26338
	Hs.657347	203640_at	MBNL2	muscleblind-like splicing regulator 2	-1.26329
	Hs.156178	203501_at	CPQ	carboxypeptidase Q	-1.26292
	Hs.476636	206144_at	MAGI1	membrane associated guanylate kinase, WW and PDZ domain containing 1	-1.26275
	Hs.84549	232146_at	NDUFC1	NADH dehydrogenase (ubiquinone) 1, subcomplex unknown, 1, 6kDa	-1.26272
	Hs.189409	212288_at	FNBP1	formin binding protein 1	-1.26249
	Hs.372360	37549_g_at	BBS9	Bardet-Biedl syndrome 9	-1.26235
	Hs.433381	225729_at	C6orf89	chromosome 6 open reading frame 89	-1.26221
	Hs.95243	204045_at	TCEAL1	transcription elongation factor A (SII)-like 1	-1.26211
	Hs.512776	223690_at	LTBP2	latent transforming growth factor beta binding protein 2	-1.26071
	Hs.44685	219104_at	RNF141	ring finger protein 141	-1.26057
	Hs.161000	205062_x_at	ARID4A	AT rich interactive domain 4A (RBP1-like)	-1.26
	Hs.43233	209108_at	TSPAN6	tetraspanin 6	-1.25888
	Hs.121520	222108_at	AMIGO2	adhesion molecule with Ig-like domain 2	-1.2587
	Hs.47382	225373_at	C10orf54	chromosome 10 open reading frame 54	-1.2586
	Hs.522350	226249_at	SNX30	sorting nexin family member 30	-1.25825
	Hs.111867	228537_at	GLI2	GLI family zinc finger 2	-1.25791
	Hs.406787	238686_at	FBXO3	F-box protein 3	-1.25784
	Hs.371240	227530_at	AKAP12	A kinase (PRKA) anchor protein 12	-1.25769
	Hs.498892	225545_at	EEF2K	eukaryotic elongation factor-2 kinase	-1.25627
	Hs.6917	216862_s_at	MTCP1NB	mature T-cell proliferation 1 neighbor	-1.2561
	Hs.389452	223373_s_at	PLA2G12A	phospholipase A2, group XIIA	-1.25584
	Hs.391860	204497_at	ADCY9	adenylate cyclase 9	-1.2555
	Hs.505202	231964_at	BICD1	bicaudal D homolog 1 (Drosophila)	-1.25483
	Hs.432914	217890_s_at	PARVA	parvin, alpha	-1.25474
	---	227837_at	LOC729570	uncharacterized LOC729570	-1.25468
	Hs.7549	235346_at	FUNDC1	FUN14 domain containing 1	-1.25443
	Hs.709545	213878_at	PYROXD1	pyridine nucleotide-disulphide oxidoreductase domain 1	-1.25432
	Hs.81170	209193_at	PIM1	pim-1 oncogene	-1.25312

	Hs.466539	212358_at	CLIP3	CAP-GLY domain containing linker protein 3	-1.25286
	Hs.7200	212299_at	NEK9	NIMA (never in mitosis gene a)- related kinase 9	-1.25251
	Hs.596537	225409_at	COA5	cytochrome C oxidase assembly factor 5	-1.252
	Hs.288912	221702_s_at	TM2D3	TM2 domain containing 3	-1.25181
	Hs.288304	232048_at	FAM76B	family with sequence similarity 76, member B	-1.25093
	Hs.709187	244687_at	DBT	dihydrolipoamide branched chain transacylase E2	-1.24948
	Hs.503831	228080_at	LAYN	layilin	-1.2492
	Hs.656213	205842_s_at	JAK2	Janus kinase 2	-1.24879
	Hs.524625	204396_s_at	GRK5	G protein-coupled receptor kinase 5	-1.24842
	Hs.497332	228386_s_at	DDX59	DEAD (Asp-Glu-Ala-Asp) box polypeptide 59	-1.24836
	Hs.306083	225795_at	C22orf32	chromosome 22 open reading frame 32	-1.24782
	Hs.399891	226545_at	CD109	CD109 molecule	-1.2469
	Hs.531249	229198_at	USP35	ubiquitin specific peptidase 35	-1.24586
	Hs.558396	200832_s_at	SCD	stearoyl-CoA desaturase (delta-9-desaturase)	-1.24551
	Hs.409210	207231_at	DZIP3	DAZ interacting protein 3, zinc finger	-1.24546
	Hs.422986	201301_s_at	ANXA4	annexin A4	-1.24423
	Hs.276252	211852_s_at	ATRN	attractin	-1.24308
	Hs.285051	214152_at	CCPG1 /// DYX1C1-CCPG1	cell cycle progression 1 /// DYX1C1-CCPG1 readthrough (non-protein coding)	-1.24279
	Hs.446641	230652_at	ARAF	v-raf murine sarcoma 3611 viral oncogene homolog	-1.24219
	Hs.110364	204517_at	PPIC	peptidylprolyl isomerase C (cyclophilin C)	-1.24217
	Hs.356399	218344_s_at	RCOR3	REST corepressor 3	-1.24208
	Hs.602086	201798_s_at	MYOF	myoferlin	-1.24203
	Hs.591582	217837_s_at	CHMP3 /// RNF103-CHMP3	charged multivesicular body protein 3 /// RNF103-CHMP3 readthrough	-1.24199
	Hs.740486	224870_at	DANCR	differentiation antagonizing non-protein coding RNA	-1.24195
	Hs.292316	203428_s_at	ASF1A	ASF1 anti-silencing function 1 homolog A (S. cerevisiae)	-1.24179
	Hs.713574	218689_at	FANCF	Fanconi anemia, complementation group F	-1.2417
	Hs.709425	209984_at	KDM4C	lysine (K)-specific demethylase 4C	-1.24123
	Hs.600125	224593_at	ZNF664	zinc finger protein 664	-1.2411
	Hs.706662	218341_at	PPCS	phosphopantothenoylcysteine synthetase	-1.24084
	Hs.20107	225948_at	APOPT1 /// KLC1	apoptogenic 1, mitochondrial /// kinesin light chain 1	-1.24076
	Hs.440534	225302_at	TMX3	thioredoxin-related transmembrane protein 3	-1.24074
	Hs.520708	203695_s_at	DFNA5	deafness, autosomal dominant 5	-1.24049
	Hs.740577	213508_at	SPTSSA	serine palmitoyltransferase, small subunit A	-1.24038
	Hs.380627	223047_at	CMTM6	CKLF-like MARVEL transmembrane domain containing 6	-1.24023
	Hs.642842	218276_s_at	SAV1	salvador homolog 1 (Drosophila)	-1.23995
	Hs.180946	216044_x_at	FAM69A	family with sequence similarity 69, member A	-1.23928
	Hs.706828	202981_x_at	SIAH1	siah E3 ubiquitin protein ligase 1	-1.23924
	Hs.591289	236918_s_at	LRRC34	leucine rich repeat containing 34	-1.239
	Hs.365365	229696_at	FECH	ferrochelatase	-1.23872
	Hs.283416	226695_at	PRRX1	paired related homeobox 1	-1.23866
	Hs.529925	212760_at	UBR2	ubiquitin protein ligase E3 component n-recognin 2	-1.23862
	Hs.283652	204615_x_at	IDI1	isopentenyl-diphosphate delta isomerase 1	-1.23834
	Hs.592313	235635_at	ARHGAP5	Rho GTPase activating protein 5	-1.23809
	Hs.515016	221290_s_at	MUM1	melanoma associated antigen (mutated) 1	-1.2377
	Hs.585010	227411_at	WTIP	Wilms tumor 1 interacting protein	-1.23716
	Hs.482587	212425_at	SCAMP1	secretory carrier membrane protein 1	-1.23534
	Hs.370379	244007_at	ZNF462	zinc finger protein 462	-1.23483
	Hs.465985	202024_at	ASNA1	arsA arsenite transporter, ATP-binding, homolog 1 (bacterial)	-1.23439
	Hs.83734	229395_at	STX4	syntaxin 4	-1.23391
	---	227655_at	LOC100505806	uncharacterized LOC100505806	-1.2333

	Hs.247362	202262_x_at	DDAH2	dimethylarginine dimethylaminohydrolase 2	-1.23238
	Hs.643910	201416_at	SOX4	SRY (sex determining region Y)-box 4	-1.23223
	Hs.729098	209406_at	BAG2	BCL2-associated athanogene 2	-1.23205
	Hs.74615	203131_at	PDGFRA	platelet-derived growth factor receptor, alpha polypeptide	-1.23141
	Hs.463320	227946_at	OSBPL7	oxysterol binding protein-like 7	-1.23005
	Hs.94896	218477_at	TMEM14A	transmembrane protein 14A	-1.22943
	Hs.196102	202033_s_at	RB1CC1	RB1-inducible coiled-coil 1	-1.22892
	Hs.584744	226424_at	CAPS	calcyphosine	-1.22812
	Hs.374446	226638_at	ARHGAP23	Rho GTPase activating protein 23	-1.22729
	Hs.157078	221826_at	ANGEL2	angel homolog 2 (Drosophila)	-1.22691
	Hs.253305	1553167_a_at	SEPSECS	Sep (O-phosphoserine) tRNA:Sec (selenocysteine) tRNA synthase	-1.22683
	Hs.548197	225400_at	TSEN15	tRNA splicing endonuclease 15 homolog (S. cerevisiae)	-1.2256
	Hs.500822	221519_at	FBXW4	F-box and WD repeat domain containing 4	-1.22543
	Hs.115284	227207_x_at	ZNF213	zinc finger protein 213	-1.22527
	Hs.497575	1568955_at	SRGAP2 /// SRGAP2C	SLIT-ROBO Rho GTPase activating protein 2 /// SLIT-ROBO Rho GTPase activating protein 2	-1.22417
	Hs.132225	212239_at	PIK3R1	phosphoinositide-3-kinase, regulatory subunit 1 (alpha)	-1.22391
	Hs.497253	225675_at	C14orf101	chromosome 14 open reading frame 101	-1.22378
	Hs.121076	224364_s_at	PPIL3	peptidylprolyl isomerase (cyclophilin)-like 3	-1.2235
	Hs.591388	232641_at	ZNF596	zinc finger protein 596	-1.22314
	Hs.201918	226297_at	HIPK3	homeodomain interacting protein kinase 3	-1.22283
	Hs.709257	212675_s_at	CEP68	centrosomal protein 68kDa	-1.22226
	Hs.144502	218942_at	PIP4K2C	phosphatidylinositol-5-phosphate 4-kinase, type II, gamma	-1.22218
	Hs.371977	204866_at	PHF16	PHD finger protein 16	-1.22157
	Hs.283749	205141_at	ANG	angiogenin, ribonuclease, RNase A family, 5	-1.22077
	Hs.351798	238067_at	TBC1D8B	TBC1 domain family, member 8B (with GRAM domain)	-1.22072
	Hs.168762	204063_s_at	ULK2	unc-51-like kinase 2 (C. elegans)	-1.22016
	Hs.565319	237052_x_at	GIGYF2	GRB10 interacting GYF protein 2	-1.21958
	Hs.529989	217984_at	RNASET2	ribonuclease T2	-1.2193
	Hs.210283	203325_s_at	COL5A1	collagen, type V, alpha 1	-1.21909
	Hs.380138	212624_s_at	CHN1	chimerin (chimaerin) 1	-1.2189
	Hs.61329	1558508_a_at	C1orf53	chromosome 1 open reading frame 53	-1.21884
	Hs.482043	202784_s_at	NNT	nicotinamide nucleotide transhydrogenase	-1.21883
	Hs.23492	226509_at	ZNF641	zinc finger protein 641	-1.2188
	Hs.654560	228904_at	HOXB3	homeobox B3	-1.21864
	Hs.599469	239265_at	SLC35G1	solute carrier family 35, member G1	-1.21835
	Hs.12144	212795_at	KIAA1033	KIAA1033	-1.21803
	Hs.584884	209935_at	ATP2C1	ATPase, Ca++ transporting, type 2C, member 1	-1.21799
	Hs.573495	228486_at	SLC44A1	solute carrier family 44, member 1	-1.21792
	Hs.301685	38671_at	PLXND1	plexin D1	-1.21723
	Hs.659413	236777_at	LOC100129195	uncharacterized LOC100129195	-1.21696
	Hs.272328	228208_x_at	ZNF354C	zinc finger protein 354C	-1.21653
	Hs.477420	213069_at	HEG1	HEG homolog 1 (zebrafish)	-1.21575
	Hs.740582	213853_at	DNAJC24	DnaJ (Hsp40) homolog, subfamily C, member 24	-1.21571
	Hs.391464	202805_s_at	ABCC1	ATP-binding cassette, sub-family C (CFTR/MRP), member 1	-1.21501
	Hs.705431	210312_s_at	IFT20	intraflagellar transport 20 homolog (Chlamydomonas)	-1.21463
	Hs.607928	222480_at	UBE2Q1	ubiquitin-conjugating enzyme E2Q family member 1	-1.2141
	Hs.440776	222360_at	DPH5	DPH5 homolog (S. cerevisiae)	-1.21397
	Hs.303055	226120_at	TTC8	tetratricopeptide repeat domain 8	-1.21393
	Hs.283652	208881_x_at	IDI1	isopentenyl-diphosphate delta isomerase 1	-1.21335
	Hs.528019	221689_s_at	PIGP	phosphatidylinositol glycan anchor biosynthesis, class P	-1.21318
	Hs.105607	223748_at	SLC4A11	solute carrier family 4, sodium borate transporter, member 11	-1.21301
	Hs.724	35846_at	THRA	thyroid hormone receptor, alpha	-1.21289

	Hs.525063	221622_s_at	TMEM126B	transmembrane protein 126B	-1.21199
	Hs.520259	209626_s_at	OSBPL3	oxysterol binding protein-like 3	-1.21174
	Hs.444349	212217_at	PREPL	prolyl endopeptidase-like	-1.21153
	Hs.592078	218937_at	ZNF434	zinc finger protein 434	-1.21129
	Hs.593928	208647_at	FDFT1	farnesyl-diphosphate farnesyltransferase 1	-1.21074
	Hs.434966	212690_at	DDHD2	DDHD domain containing 2	-1.21035
	Hs.279840	206175_x_at	ZNF222	zinc finger protein 222	-1.2102
	Hs.480116	212606_at	WDFY3	WD repeat and FYVE domain containing 3	-1.20982
	Hs.389452	228084_at	PLA2G12A	phospholipase A2, group XIIA	-1.20976
	Hs.732083	203562_at	FEZ1	fasciculation and elongation protein zeta 1 (zygin I)	-1.20923
	Hs.108029	201311_s_at	SH3BGRL	SH3 domain binding glutamic acid-rich protein like	-1.2089
	Hs.44856	219815_at	GAL3ST4	galactose-3-O-sulfotransferase 4	-1.20867
	Hs.731575	235762_at	TAS2R14	taste receptor, type 2, member 14	-1.20842
	Hs.6434	218820_at	C14orf132	chromosome 14 open reading frame 132	-1.20807
	Hs.709348	235717_at	ZNF229	zinc finger protein 229	-1.20788
	Hs.61508	226924_at	LOC400657	uncharacterized LOC400657	-1.20774
	Hs.529857	226552_at	IER5L	immediate early response 5-like	-1.20747
	Hs.593645	226869_at	MEGF6	multiple EGF-like-domains 6	-1.20655
	Hs.554791	203421_at	TP53I11	tumor protein p53 inducible protein 11	-1.20634
	---	1558890_at	LOC100507054	uncharacterized LOC100507054	-1.20613
	Hs.708017	224481_s_at	HECTD1	HECT domain containing E3 ubiquitin protein ligase 1	-1.20609
	Hs.533597	225370_at	PYGO2	pygopus homolog 2 (Drosophila)	-1.20605
	Hs.302085	208131_s_at	PTGIS	prostaglandin I2 (prostacyclin) synthase	-1.20509
	Hs.494186	219147_s_at	NMRK1	nicotinamide riboside kinase 1	-1.20488
	Hs.156316	201893_x_at	DCN	decorin	-1.20472
	Hs.99488	227840_at	C2orf76	chromosome 2 open reading frame 76	-1.20461
	Hs.485915	223144_s_at	AKIRIN2	akirin 2	-1.20436
	Hs.444818	224599_at	CGGBP1	CGG triplet repeat binding protein 1	-1.20423
	Hs.82116	209124_at	MYD88	myeloid differentiation primary response gene (88)	-1.20418
	Hs.91747	204992_s_at	PFN2	profilin 2	-1.20397
	Hs.531704	213093_at	PRKCA	protein kinase C, alpha	-1.20319
	Hs.133135	225093_at	UTRN	utrophin	-1.20319
	---	215160_x_at	LOC100289097	protein FRG1-like	-1.20237
	Hs.410378	224002_s_at	FKBP7	FK506 binding protein 7	-1.20215
	Hs.253903	201060_x_at	STOM	stomatin	-1.20151
	Hs.631730	218983_at	C1RL	complement component 1, r subcomponent-like	-1.20054
	Hs.505339	224906_at	ANO6	anoctamin 6	-1.20047
	Hs.505729	225776_at	RBMS2	RNA binding motif, single stranded interacting protein 2	-1.20011
	Hs.502705	203103_s_at	PRPF19	PRP19/PSO4 pre-mRNA processing factor 19 homolog (S. cerevisiae)	1.20039
	Hs.26613	212714_at	LARP4	La ribonucleoprotein domain family, member 4	1.20046
	Hs.54609	205164_at	GCAT	glycine C-acetyltransferase	1.20061
	Hs.99196	224480_s_at	AGPAT9	1-acylglycerol-3-phosphate O-acyltransferase 9	1.20061
	Hs.740445	200995_at	IPO7	importin 7	1.20062
	Hs.349150	226762_at	PURB	purine-rich element binding protein B	1.20221
	Hs.181444	222987_s_at	TMEM9	transmembrane protein 9	1.20259
	Hs.418533	201457_x_at	BUB3	budding uninhibited by benzimidazoles 3 homolog (yeast)	1.20285
	Hs.48513	204434_at	SPATA2	spermatogenesis associated 2	1.20289
	Hs.327527	208794_s_at	SMARCA4	SWI/SNF related, matrix associated, actin dependent regulator of chromatin, subfamily a	1.20334
	Hs.647333	209836_x_at	BOLA2 /// BOLA2B	bolA homolog 2 (E. coli) /// bolA homolog 2B (E. coli)	1.20357
	Hs.89497	203276_at	LMNB1	lamin B1	1.20399
	Hs.137282	228229_at	ZNF526	zinc finger protein 526	1.20455

	Hs.7165	200054_at	ZNF259	zinc finger protein 259	1.20472
	Hs.132314	219134_at	ELTD1	EGF, latrophilin and seven transmembrane domain containing 1	1.20526
	Hs.9061	221637_s_at	C11orf48	chromosome 11 open reading frame 48	1.20557
	Hs.525198	205339_at	STIL	SCL/TAL1 interrupting locus	1.20578
	Hs.116665	239835_at	KBTBD8	kelch repeat and BTB (POZ) domain containing 8	1.20798
	Hs.679430	235512_at	CDKL1	cyclin-dependent kinase-like 1 (CDC2-related kinase)	1.20815
	Hs.408458	204022_at	WWP2	WW domain containing E3 ubiquitin protein ligase 2	1.20848
	Hs.143250	201645_at	TNC	tenascin C	1.20906
	Hs.632268	219512_at	DSN1	DSN1, MIND kinetochore complex component, homolog (S. cerevisiae)	1.20927
	Hs.431081	230083_at	USP53	ubiquitin specific peptidase 53	1.21016
	Hs.364544	217979_at	TSPAN13	tetraspanin 13	1.21167
	Hs.591671	201521_s_at	NCBP2	nuclear cap binding protein subunit 2, 20kDa	1.21171
	Hs.532793	208974_x_at	KPNB1	karyopherin (importin) beta 1	1.21207
	Hs.731750	203126_at	IMPA2	inositol(myo)-1(or 4)-monophosphatase 2	1.21223
	Hs.466714	202093_s_at	PAF1	Paf1, RNA polymerase II associated factor, homolog (S. cerevisiae)	1.21228
	Hs.655259	224333_s_at	MRPS5	mitochondrial ribosomal protein S5	1.2123
	Hs.495984	211208_s_at	CASK	calcium/calmodulin-dependent serine protein kinase (MAGUK family)	1.21242
	Hs.587054	201503_at	G3BP1	GTPase activating protein (SH3 domain) binding protein 1	1.21296
	Hs.35125	58780_s_at	ARHGEF40	Rho guanine nucleotide exchange factor (GEF) 40	1.21341
	Hs.22616	212456_at	KIAA0664	KIAA0664	1.21345
	Hs.631757	209965_s_at	RAD51D	RAD51 homolog D (S. cerevisiae)	1.21354
	Hs.719958	213648_at	EXOSC7	exosome component 7	1.21446
	Hs.215766	218238_at	GTPBP4	GTP binding protein 4	1.21462
	Hs.513268	218524_at	E4F1	E4F transcription factor 1	1.21567
	Hs.502659	200885_at	RHOC	ras homolog family member C	1.21602
	Hs.643464	219544_at	BORA	bora, aurora kinase A activator	1.21693
	Hs.276878	202188_at	NUP93	nucleoporin 93kDa	1.2179
	Hs.529451	209190_s_at	DIAPH1	diaphanous homolog 1 (Drosophila)	1.21805
	Hs.720208	204647_at	HOMER3	homer homolog 3 (Drosophila)	1.21833
	Hs.522675	208117_s_at	LAS1L	LAS1-like (S. cerevisiae)	1.21857
	Hs.515154	223419_at	FBXW9	F-box and WD repeat domain containing 9	1.21899
	Hs.27621	205405_at	SEMA5A	sema domain, seven thrombospondin repeats (type 1 and type 1-like), transmembrane domai	1.22033
	Hs.83765	202534_x_at	DHFR	dihydrofolate reductase	1.22048
	Hs.143703	229074_at	EHD4	EH-domain containing 4	1.22123
	Hs.731548	222692_s_at	FNDC3B	fibronectin type III domain containing 3B	1.22203
	Hs.584807	223274_at	TCF19	transcription factor 19	1.22218
	Hs.655373	224677_x_at	C11orf31	chromosome 11 open reading frame 31	1.22316
	Hs.69554	223332_x_at	RNF126	ring finger protein 126	1.22331
	Hs.720388	218112_at	MRPS34	mitochondrial ribosomal protein S34	1.22333
	Hs.9589	222991_s_at	UBQLN1	ubiquilin 1	1.22354
	Hs.280387	205282_at	LRP8	low density lipoprotein receptor-related protein 8, apolipoprotein e receptor	1.22359
	Hs.209989	222505_at	LMBR1	limb region 1 homolog (mouse)	1.22412
	Hs.1004	204481_at	BRPF1	bromodomain and PHD finger containing, 1	1.22449
	Hs.194754	204699_s_at	DIEXF	digestive organ expansion factor homolog (zebrafish)	1.22476
	Hs.368084	211615_s_at	LRPPRC	leucine-rich pentatricopeptide repeat containing	1.2248
	Hs.124299	226614_s_at	FAM167A	family with sequence similarity 167, member A	1.2248
	Hs.520506	218875_s_at	FBXO5	F-box protein 5	1.22497
	Hs.115474	204127_at	RFC3	replication factor C (activator 1) 3, 38kDa	1.22587
	Hs.592081	233049_x_at	STUB1	STIP1 homology and U-box containing protein 1, E3 ubiquitin protein ligase	1.22597
	Hs.534770	201251_at	PKM	pyruvate kinase, muscle	1.22602
	Hs.513379	223513_at	CENPJ	centromere protein J	1.2261
	Hs.517145	201231_s_at	ENO1	enolase 1, (alpha)	1.22644

	Hs.406307	244640_at	ZNF850	zinc finger protein 850	1.22692
	Hs.62604	235417_at	SPOCD1	SPOC domain containing 1	1.22828
	Hs.513470	212809_at	NFATC2IP	nuclear factor of activated T-cells, cytoplasmic, calcineurin-dependent 2 interacting p	1.2287
	Hs.16803	223546_x_at	LUC7L	LUC7-like (S. cerevisiae)	1.2291
	Hs.479602	40148_at	APBB2	amyloid beta (A4) precursor protein-binding, family B, member 2	1.22943
	Hs.397638	225170_at	WDR5	WD repeat domain 5	1.2299
	Hs.371001	208688_x_at	EIF3B	eukaryotic translation initiation factor 3, subunit B	1.23001
	Hs.127432	236649_at	DTWD1	DTW domain containing 1	1.231
	Hs.487341	221737_at	GNA12	guanine nucleotide binding protein (G protein) alpha 12	1.23123
	Hs.126221	215269_at	TRAPPC10	trafficking protein particle complex 10	1.23193
	Hs.485628	1556060_a_at	ZNF451	zinc finger protein 451	1.232
	Hs.505469	222077_s_at	RACGAP1	Rac GTPase activating protein 1	1.23216
	Hs.2399	160020_at	MMP14	matrix metallopeptidase 14 (membrane-inserted)	1.23263
	Hs.620541	227374_at	EARS2	glutamyl-tRNA synthetase 2, mitochondrial (putative)	1.23295
	Hs.520026	201797_s_at	VARS	valyl-tRNA synthetase	1.23355
	Hs.729312	219384_s_at	ADAT1	adenosine deaminase, tRNA-specific 1	1.23358
	Hs.90073	210766_s_at	CSE1L	CSE1 chromosome segregation 1-like (yeast)	1.2338
	Hs.145442	202670_at	MAP2K1	mitogen-activated protein kinase kinase 1	1.23523
	Hs.519972	221875_x_at	HLA-F	major histocompatibility complex, class I, F	1.23582
	Hs.515846	1559946_s_at	RUVBL2	RuvB-like 2 (E. coli)	1.23592
	Hs.370555	223166_x_at	RABL6	RAB, member RAS oncogene family-like 6	1.23592
	Hs.123253	219493_at	SHCBP1	SHC SH2-domain binding protein 1	1.2362
	Hs.407926	228248_at	RICTOR	RPTOR independent companion of MTOR, complex 2	1.2367
	Hs.21331	222606_at	ZWILCH	Zwilch, kinetochore associated, homolog (Drosophila)	1.23683
	Hs.46894	200959_at	FUS	fused in sarcoma	1.23697
	Hs.647156	208511_at	PTTG3P	pituitary tumor-transforming 3, pseudogene	1.23785
	Hs.449278	220155_s_at	BRD9	bromodomain containing 9	1.23819
	Hs.374950	204326_x_at	MT1X	metallothionein 1X	1.2396
	Hs.300624	200020_at	TARDBP	TAR DNA binding protein	1.24013
	Hs.250822	204092_s_at	AURKA	aurora kinase A	1.24058
	Hs.310458	227236_at	TSPAN2	tetraspanin 2	1.24064
	Hs.519672	202384_s_at	TCOF1	Treacher Collins-Franceschetti syndrome 1	1.24068
	Hs.373550	1566901_at	TGIF1	TGFB-induced factor homeobox 1	1.24103
	Hs.5719	201774_s_at	NCAPD2	non-SMC condensin I complex, subunit D2	1.2419
	Hs.463416	219401_at	XYLT2	xylosyltransferase II	1.24233
	Hs.489287	206688_s_at	CPSF4	cleavage and polyadenylation specific factor 4, 30kDa	1.24403
	Hs.740459	201364_s_at	OAZ2	ornithine decarboxylase antizyme 2	1.24456
	Hs.246506	202825_at	SLC25A4	solute carrier family 25 (mitochondrial carrier; adenine nucleotide translocator), memb	1.24576
	Hs.521924	209899_s_at	PUF60	poly-U binding splicing factor 60KDa	1.24581
	Hs.731801	209100_at	IFRD2	interferon-related developmental regulator 2	1.24657
	Hs.508829	1552613_s_at	CDC42SE2	CDC42 small effector 2	1.24673
	Hs.178728	202463_s_at	MBD3	methyl-CpG binding domain protein 3	1.24691
	Hs.23413	227700_x_at	ATAD3A	ATPase family, AAA domain containing 3A	1.24729
	Hs.374378	201897_s_at	CKS1B	CDC28 protein kinase regulatory subunit 1B	1.24752
	Hs.24763	202483_s_at	RANBP1	RAN binding protein 1	1.24866
	Hs.511903	227894_at	WDR90	WD repeat domain 90	1.24942
	Hs.730765	33307_at	RRP7A	ribosomal RNA processing 7 homolog A (S. cerevisiae)	1.24971
	Hs.731613	224232_s_at	PRELID1	PRELI domain containing 1	1.25
	Hs.93659	208658_at	PDIA4	protein disulfide isomerase family A, member 4	1.25001
	Hs.696684	201466_s_at	JUN	jun proto-oncogene	1.25021
	Hs.701398	209042_s_at	UBE2G2	ubiquitin-conjugating enzyme E2G 2	1.25023
	---	215395_x_at	PRSS3P2	protease, serine, 3 pseudogene 2	1.25043
	Hs.108080	200621_at	CSRP1	cysteine and glycine-rich protein 1	1.25305

	Hs.3887	201199_s_at	PSMD1	proteasome (prosome, macropain) 26S subunit, non-ATPase, 1	1.25373
	---	213164_at	SLC5A3	solute carrier family 5 (sodium/myo-inositol cotransporter), member 3	1.25627
	Hs.658939	219306_at	KIF15	kinesin family member 15	1.25633
	Hs.644056	212075_s_at	CSNK2A1 /// CSNK2A1P	casein kinase 2, alpha 1 polypeptide /// casein kinase 2, alpha 1 polypeptide pseudogen	1.25664
	Hs.369762	202589_at	TYMS	thymidylate synthetase	1.25751
	Hs.501928	212473_s_at	MICAL2	microtubule associated monoxygenase, calponin and LIM domain containing 2	1.25782
	Hs.654958	209246_at	ABCF2	ATP-binding cassette, sub-family F (GCN20), member 2	1.25828
	Hs.520063	209196_at	WDR46	WD repeat domain 46	1.25831
	Hs.675399	216176_at	HCRP1	hepatocellular carcinoma-related HCRP1	1.25893
	Hs.272062	200636_s_at	PTPRF	protein tyrosine phosphatase, receptor type, F	1.2595
	Hs.309231	225788_at	RRP36	ribosomal RNA processing 36 homolog (S. cerevisiae)	1.2599
	Hs.731908	203209_at	RFC5	replication factor C (activator 1) 5, 36.5kDa	1.26018
	Hs.365116	232141_at	U2AF1	U2 small nuclear RNA auxiliary factor 1	1.26203
	Hs.330663	220060_s_at	PARPBP	PARP1 binding protein	1.2624
	Hs.647620	1555783_x_at	PQLC2	PQ loop repeat containing 2	1.26259
	Hs.114033	226712_at	SSR1	signal sequence receptor, alpha	1.26265
	Hs.59425	1554082_a_at	NOL9	nucleolar protein 9	1.26297
	Hs.709864	223622_s_at	HYI	hydroxypyruvate isomerase (putative)	1.26459
	Hs.731917	223249_at	CLDN12	claudin 12	1.26491
	Hs.130849	219575_s_at	COG8 /// PDF	component of oligomeric golgi complex 8 /// peptide deformylase (mitochondrial)	1.26511
	Hs.272011	238987_at	B4GALT1	UDP-Gal:betaGlcNAc beta 1,4- galactosyltransferase, polypeptide 1	1.26734
	Hs.390567	216033_s_at	FYN	FYN oncogene related to SRC, FGR, YES	1.26743
	Hs.433203	221597_s_at	TMEM208	transmembrane protein 208	1.26775
	Hs.311100	229863_s_at	C3orf75	chromosome 3 open reading frame 75	1.26812
	Hs.82609	203040_s_at	HMBS	hydroxymethylbilane synthase	1.26899
	---	212944_at	SLC5A3	solute carrier family 5 (sodium/myo-inositol cotransporter), member 3	1.26917
	Hs.570855	218718_at	PDGFC	platelet derived growth factor C	1.26939
	Hs.129742	207601_at	SULT1B1	sulfotransferase family, cytosolic, 1B, member 1	1.27025
	Hs.632191	218069_at	DCTPP1	dCTP pyrophosphatase 1	1.27099
	Hs.311100	229864_at	C3orf75	chromosome 3 open reading frame 75	1.271
	Hs.569009	229099_at	C11orf83	chromosome 11 open reading frame 83	1.27159
	Hs.658304	226609_at	DCBLD1	discoidin, CUB and LCCL domain containing 1	1.27283
	Hs.690826	219582_at	OGFRL1	opioid growth factor receptor-like 1	1.27302
	Hs.654350	204618_s_at	GABPB1	GA binding protein transcription factor, beta subunit 1	1.27432
	Hs.83765	202533_s_at	DHFR	dihydrofolate reductase	1.27459
	Hs.208701	229442_at	C18orf54	chromosome 18 open reading frame 54	1.27466
	Hs.514505	212723_at	JMJD6	jumonji domain containing 6	1.27561
	Hs.263812	210574_s_at	NUDC	nuclear distribution C homolog (A. nidulans)	1.27631
	Hs.3104	236641_at	KIF14	kinesin family member 14	1.27668
	Hs.591495	229538_s_at	IQGAP3	IQ motif containing GTPase activating protein 3	1.2769
	Hs.594537	213790_at	ADAM12	ADAM metallopeptidase domain 12	1.27792
	Hs.520215	206860_s_at	MIOS	missing oocyte, meiosis regulator, homolog (Drosophila)	1.27814
	Hs.435215	209946_at	VEGFC	vascular endothelial growth factor C	1.27883
	Hs.374477	210011_s_at	EWSR1	Ewing sarcoma breakpoint region 1	1.28079
	Hs.533549	218488_at	EIF2B3	eukaryotic translation initiation factor 2B, subunit 3 gamma, 58kDa	1.28081
	Hs.287714	204214_s_at	RAB32	RAB32, member RAS oncogene family	1.28239
	Hs.370671	213300_at	ATG2A	autophagy related 2A	1.28281
	Hs.186486	203836_s_at	MAP3K5	mitogen-activated protein kinase kinase kinase 5	1.28386
	Hs.381178	220588_at	BCAS4	breast carcinoma amplified sequence 4	1.28769
	Hs.740467	207165_at	HMMR	hyaluronan-mediated motility receptor (RHAMM)	1.28775
	Hs.567378	211387_x_at	RNGTT	RNA guanylyltransferase and 5'-phosphatase	1.2882
	Hs.422662	203856_at	VRK1	vaccinia related kinase 1	1.2901

	Hs.121536	228069_at	FAM54A	family with sequence similarity 54, member A	1.29103
	Hs.517168	200916_at	TAGLN2	transgelin 2	1.29201
	Hs.445705	201477_s_at	RRM1	ribonucleotide reductase M1	1.29285
	Hs.368307	229649_at	NRXN3	neurexin 3	1.293
	Hs.614194	202519_at	MLXIP	MLX interacting protein	1.29417
	Hs.424312	211564_s_at	PDLIM4	PDZ and LIM domain 4	1.29431
	Hs.319334	201970_s_at	NASP	nuclear autoantigenic sperm protein (histone-binding)	1.29624
	Hs.39311	218800_at	SRD5A3	steroid 5 alpha-reductase 3	1.29677
	Hs.577404	1558369_at	MPHOSPH9	M-phase phosphoprotein 9	1.29783
	Hs.129055	210415_s_at	ODF2	outer dense fiber of sperm tails 2	1.29931
	Hs.376015	240402_at	KIRREL3	kin of IRRE like 3 (Drosophila)	1.29959
	Hs.184339	204825_at	MELK	maternal embryonic leucine zipper kinase	1.30081
	Hs.722525	232740_at	MCM3AP-AS1	MCM3AP antisense RNA 1 (non-protein coding)	1.30091
	Hs.9914	204948_s_at	FST	follistatin	1.30113
	Hs.119882	243000_at	CDK6	cyclin-dependent kinase 6	1.30144
	Hs.25300	215134_at	PI4K2A	phosphatidylinositol 4-kinase type 2 alpha	1.30201
	Hs.91586	209149_s_at	TM9SF1	transmembrane 9 superfamily member 1	1.30292
	Hs.520525	226930_at	FNDC1	fibronectin type III domain containing 1	1.30314
	Hs.189073	1555274_a_at	EPT1	ethanolaminephosphotransferase 1 (CDP-ethanolamine-specific)	1.30357
	Hs.592011	222767_s_at	C12orf49	chromosome 12 open reading frame 49	1.30508
	Hs.75573	205046_at	CENPE	centromere protein E, 312kDa	1.30513
	Hs.591040	212621_at	TMEM194A	transmembrane protein 194A	1.30543
	---	225767_at	RN45S	45S pre-ribosomal RNA	1.30581
	Hs.109059	203931_s_at	MRPL12	mitochondrial ribosomal protein L12	1.30624
	Hs.522933	229348_at	UBIAD1	UbiA prenyltransferase domain containing 1	1.30644
	Hs.433764	203602_s_at	ZBTB17	zinc finger and BTB domain containing 17	1.30712
	Hs.5662	222034_at	GNB2L1 /// LOC100289627 /// SNORD95 /// SNORD96A	guanine nucleotide binding protein (G protein), beta polypeptide 2-like 1 /// uncharact	1.30713
	Hs.164226	201109_s_at	THBS1	thrombospondin 1	1.30742
	Hs.133512	220104_at	ZC3HAV1	zinc finger CCCH-type, antiviral 1	1.30975
	Hs.731712	204033_at	TRIP13	thyroid hormone receptor interactor 13	1.31011
	---	205241_at	SCO2	SCO cytochrome oxidase deficient homolog 2 (yeast)	1.31013
	Hs.709550	221879_at	CALML4	calmodulin-like 4	1.31022
	Hs.531561	204975_at	EMP2	epithelial membrane protein 2	1.31027
	Hs.263812	201173_x_at	NUDC	nuclear distribution C homolog (A. nidulans)	1.31043
	Hs.444441	211136_s_at	CLPTM1	cleft lip and palate associated transmembrane protein 1	1.31288
	Hs.591908	202759_s_at	AKAP2 /// PALM2-AKAP2	A kinase (PRKA) anchor protein 2 /// PALM2-AKAP2 readthrough	1.31344
	Hs.311190	225201_s_at	MRPL14	mitochondrial ribosomal protein L14	1.31347
	Hs.374257	208322_s_at	ST3GAL1	ST3 beta-galactoside alpha-2,3-sialyltransferase 1	1.31352
	Hs.696283	214011_s_at	NOP16	NOP16 nucleolar protein homolog (yeast)	1.31403
	Hs.211571	203746_s_at	HCCS	holocytochrome c synthase	1.31471
	Hs.741061	223773_s_at	SNHG12 /// SNORA16A /// SNORA44 /// SNORA61	small nucleolar RNA host gene 12 (non-protein coding) /// small nucleolar RNA, H/ACA bo	1.31503
	Hs.732155	212660_at	PHF15	PHD finger protein 15	1.3156
	Hs.591110	202070_s_at	IDH3A	isocitrate dehydrogenase 3 (NAD+) alpha	1.31586
	Hs.9911	218214_at	C12orf44	chromosome 12 open reading frame 44	1.31718
	Hs.436896	231763_at	POLR3A	polymerase (RNA) III (DNA directed) polypeptide A, 155kDa	1.3191
	Hs.664877	232568_at	MGC24103	uncharacterized MGC24103	1.31997
	Hs.597484	224320_s_at	MCM8	minichromosome maintenance complex component 8	1.32096
	Hs.414795	202627_s_at	SERPINE1	serpin peptidase inhibitor, clade E (nexin, plasminogen activator inhibitor type 1), me	1.32268
	Hs.632041	218695_at	EXOSC4	exosome component 4	1.32599

	Hs.20136	205088_at	MAMLD1	mastermind-like domain containing 1	1.32612
	Hs.522255	234192_s_at	GKAP1	G kinase anchoring protein 1	1.3272
	Hs.432945	230972_at	ANKRD9	ankyrin repeat domain 9	1.32746
	Hs.164419	224612_s_at	DNAJC5	DnaJ (Hsp40) homolog, subfamily C, member 5	1.32796
	Hs.487294	213468_at	ERCC2	excision repair cross-complementing rodent repair deficiency, complementation group 2	1.32841
	Hs.368921	204345_at	COL16A1	collagen, type XVI, alpha 1	1.32972
	Hs.464210	201079_at	SYNGR2	synaptogyrin 2	1.33005
	Hs.35125	241627_x_at	ARHGEF40	Rho guanine nucleotide exchange factor (GEF) 40	1.33068
	Hs.497200	210145_at	PLA2G4A	phospholipase A2, group IVA (cytosolic, calcium-dependent)	1.33175
	Hs.366401	218009_s_at	PRC1	protein regulator of cytokinesis 1	1.33257
	Hs.567567	218663_at	NCAPG	non-SMC condensin I complex, subunit G	1.33337
	Hs.558536	218860_at	NOC4L	nucleolar complex associated 4 homolog (S. cerevisiae)	1.33348
	Hs.467304	206926_s_at	IL11	interleukin 11	1.33359
	Hs.505545	210047_at	SLC11A2	solute carrier family 11 (proton-coupled divalent metal ion transporters), member 2	1.33396
	Hs.271044	209528_s_at	TELO2	TEL2, telomere maintenance 2, homolog (S. cerevisiae)	1.33461
	Hs.740467	209709_s_at	HMMR	hyaluronan-mediated motility receptor (RHAMM)	1.33764
	Hs.521092	231876_at	TRIM56	tripartite motif containing 56	1.33765
	Hs.516105	202274_at	ACTG2	actin, gamma 2, smooth muscle, enteric	1.33829
	Hs.502244	215190_at	EIF3M	eukaryotic translation initiation factor 3, subunit M	1.33859
	Hs.514284	200759_x_at	NFE2L1	nuclear factor (erythroid-derived 2)-like 1	1.33967
	---	221649_s_at	PPAN /// PPAN-P2RY11	peter pan homolog (Drosophila) /// PPAN-P2RY11 readthrough	1.33987
	Hs.6638	232676_x_at	MYEF2	myelin expression factor 2	1.34004
	Hs.121536	234944_s_at	FAM54A	family with sequence similarity 54, member A	1.34008
	Hs.113876	209054_s_at	WHSC1	Wolf-Hirschhorn syndrome candidate 1	1.3415
	Hs.471873	203270_at	DTYMK	deoxythymidylate kinase (thymidylate kinase)	1.34276
	Hs.509229	242711_x_at	FANCM	Fanconi anemia, complementation group M	1.34291
	Hs.434494	240257_at	SYNJ2	synaptojanin 2	1.34304
	Hs.530381	224739_at	PIM3	pim-3 oncogene	1.34349
	Hs.380857	224578_at	RCC2	regulator of chromosome condensation 2	1.34407
	Hs.505575	219956_at	GALNT6	UDP-N-acetyl-alpha-D-galactosamine:polypeptide N-acetylgalactosaminyltransferase 6 (Gal	1.3451
	Hs.510172	212642_s_at	HIVEP2	human immunodeficiency virus type I enhancer binding protein 2	1.34572
	Hs.311609	201584_s_at	DDX39A	DEAD (Asp-Glu-Ala-Asp) box polypeptide 39A	1.34632
	Hs.119251	201903_at	UQCRC1	ubiquinol-cytochrome c reductase core protein I	1.34662
	Hs.29344	213191_at	TICAM1	toll-like receptor adaptor molecule 1	1.34787
	Hs.119882	224847_at	CDK6	cyclin-dependent kinase 6	1.34796
	Hs.530284	209161_at	PRPF4	PRP4 pre-mRNA processing factor 4 homolog (yeast)	1.34894
	Hs.22907	213725_x_at	XYLT1	xylosyltransferase I	1.34979
	Hs.407190	224840_at	FKBP5	FK506 binding protein 5	1.35069
	Hs.657377	210109_at	SND1-IT1	SND1 intronic transcript 1 (non-protein coding)	1.35084
	Hs.405925	201896_s_at	PSRC1	proline/serine-rich coiled-coil 1	1.35151
	Hs.34333	242324_x_at	CCBE1	collagen and calcium binding EGF domains 1	1.35164
	Hs.599966	202934_at	HK2	hexokinase 2	1.35184
	Hs.306051	221029_s_at	WNT5B	wingless-type MMTV integration site family, member 5B	1.35198
	Hs.166244	219709_x_at	FAM173A	family with sequence similarity 173, member A	1.35327
	Hs.534334	214427_at	NOP2	NOP2 nucleolar protein homolog (yeast)	1.35327
	Hs.194143	204531_s_at	BRCA1	breast cancer 1, early onset	1.35359
	Hs.9661	202659_at	PSMB10	proteasome (prosome, macropain) subunit, beta type, 10	1.35589
	Hs.632365	227617_at	TMEM201	transmembrane protein 201	1.35601
	Hs.471873	1553984_s_at	DTYMK	deoxythymidylate kinase (thymidylate kinase)	1.35616
	Hs.482910	219759_at	ERAP2	endoplasmic reticulum aminopeptidase 2	1.35804
	Hs.531856	228238_at	GAS5 /// SNORD44 /// SNORD47 ///	growth arrest-specific 5 (non-protein coding) /// small nucleolar RNA, C/D box 44 /// s	1.35888

			SNORD76 /// SNORD77 /// SNORD79 /// SNORD80 /// SNORD81		
	Hs.567567	218662_s_at	NCAPG	non-SMC condensin I complex, subunit G	1.3593
	Hs.2057	215165_x_at	UMPS	uridine monophosphate synthetase	1.35957
	Hs.38449	227487_s_at	SERPINE2	Serpin peptidase inhibitor, clade E (nexin, plasminogen activator inhibitor type 1), me	1.36038
	Hs.108106	225655_at	UHRF1	ubiquitin-like with PHD and ring finger domains 1	1.36209
	Hs.91586	238948_at	TM9SF1	Transmembrane 9 superfamily member 1	1.36368
	Hs.558764	201762_s_at	PSME2	proteasome (prosome, macropain) activator subunit 2 (PA28 beta)	1.36403
	Hs.332706	202074_s_at	OPTN	optineurin	1.3659
	Hs.351474	212858_at	PAQR4	progestin and adipoQ receptor family member IV	1.36603
	Hs.731673	209233_at	EMG1	EMG1 nucleolar protein homolog (S. cerevisiae)	1.36778
	Hs.515610	209229_s_at	PPP6R1	protein phosphatase 6, regulatory subunit 1	1.368
	Hs.272062	200637_s_at	PTPRF	protein tyrosine phosphatase, receptor type, F	1.36885
	Hs.253319	225903_at	PIGU	phosphatidylinositol glycan anchor biosynthesis, class U	1.36895
	Hs.732098	204023_at	RFC4	replication factor C (activator 1) 4, 37kDa	1.36912
	Hs.529618	207332_s_at	TFRC	transferrin receptor (p90, CD71)	1.37025
	Hs.474833	226858_at	CSNK1E	casein kinase 1, epsilon	1.37035
	Hs.726442	225687_at	FAM83D	family with sequence similarity 83, member D	1.3714
	Hs.42957	204027_s_at	METTL1	methyltransferase like 1	1.37216
	Hs.534339	205053_at	PRIM1	primase, DNA, polypeptide 1 (49kDa)	1.373
	Hs.446429	212187_x_at	PTGDS	prostaglandin D2 synthase 21kDa (brain)	1.37455
	Hs.647062	1053_at	RFC2	replication factor C (activator 1) 2, 40kDa	1.37548
	Hs.523710	212860_at	ZDHHC18	zinc finger, DHHC-type containing 18	1.37564
	Hs.78769	203235_at	THOP1	thimet oligopeptidase 1	1.37754
	Hs.277035	225102_at	MGLL	monoglyceride lipase	1.38075
	Hs.632310	221191_at	STAG3L1	stromal antigen 3-like 1	1.38266
	Hs.193832	224634_at	GPATCH4	G patch domain containing 4	1.38323
	Hs.160550	226629_at	SLC43A2	solute carrier family 43, member 2	1.38327
	Hs.514012	207667_s_at	MAP2K3	mitogen-activated protein kinase kinase 3	1.3835
	Hs.162777	205909_at	POLE2	polymerase (DNA directed), epsilon 2, accessory subunit	1.38549
	Hs.660810	201478_s_at	DKC1 /// SNORA56	dyskeratosis congenita 1, dyskerin /// small nucleolar RNA, H/ACA box 56	1.38651
	Hs.740534	225583_at	UXS1	UDP-glucuronate decarboxylase 1	1.38776
	Hs.513926	215113_s_at	SENP3 /// SENP3-EIF4A1	SUMO1/sentrin/SMT3 specific peptidase 3 /// SENP3-EIF4A1 readthrough	1.38777
	Hs.713611	220668_s_at	DNMT3B	DNA (cytosine-5-)-methyltransferase 3 beta	1.38784
	Hs.191539	227214_at	GOPC	golgi-associated PDZ and coiled-coil motif containing	1.38795
	Hs.356076	235222_x_at	XIAP	X-linked inhibitor of apoptosis	1.38826
	Hs.4055	208961_s_at	KLF6	Kruppel-like factor 6	1.38839
	Hs.740401	202095_s_at	BIRC5	baculoviral IAP repeat containing 5	1.38858
	Hs.436023	228964_at	PRDM1	PR domain containing 1, with ZNF domain	1.39024
	Hs.433422	226611_s_at	CENPV	centromere protein V	1.39028
	Hs.208912	218741_at	CENPM	centromere protein M	1.39034
	Hs.311187	219229_at	SLCO3A1	solute carrier organic anion transporter family, member 3A1	1.39091
	Hs.2128	209457_at	DUSP5	dual specificity phosphatase 5	1.39296
	Hs.643480	225898_at	WDR54	WD repeat domain 54	1.39334
	Hs.656	205167_s_at	CDC25C	cell division cycle 25 homolog C (S. pombe)	1.3935
	Hs.497741	209172_s_at	CENPF	centromere protein F, 350/400kDa (mitosin)	1.39371
	Hs.514012	215498_s_at	MAP2K3	mitogen-activated protein kinase kinase 3	1.39402
	Hs.488240	203234_at	UPP1	uridine phosphorylase 1	1.39459
	---	228879_at	SNORD104	small nucleolar RNA, C/D box 104	1.39631
	Hs.231750	218587_s_at	POGLUT1	protein O-glucosyltransferase 1	1.39847

	Hs.510172	212641_at	HIVEP2	human immunodeficiency virus type I enhancer binding protein 2	1.39886
	Hs.607318	204318_s_at	GTSE1	G-2 and S-phase expressed 1	1.39886
	Hs.183817	226035_at	USP31	ubiquitin specific peptidase 31	1.40114
	Hs.239499	212424_at	PDCD11	programmed cell death 11	1.40194
	Hs.607318	215942_s_at	GTSE1	G-2 and S-phase expressed 1	1.40245
	Hs.82502	239816_at	POLD3	polymerase (DNA-directed), delta 3, accessory subunit	1.40314
	Hs.173135	202969_at	DYRK2	dual-specificity tyrosine-(Y)-phosphorylation regulated kinase 2	1.4037
	Hs.154510	205379_at	CBR3	carbonyl reductase 3	1.40383
	Hs.348920	207590_s_at	CENPI	centromere protein I	1.40441
	Hs.643599	228128_x_at	PAPPA	pregnancy-associated plasma protein A, pappalysin 1	1.40452
	Hs.256301	224468_s_at	C19orf48	chromosome 19 open reading frame 48	1.40457
	Hs.523774	209037_s_at	EHD1	EH-domain containing 1	1.40615
	Hs.187763	202102_s_at	BRD4	bromodomain containing 4	1.4069
	Hs.370834	228401_at	ATAD2	ATPase family, AAA domain containing 2	1.40733
	Hs.524399	1568596_a_at	TROAP	trophinin associated protein (tastin)	1.40783
	Hs.658046	229759_s_at	VEPH1	ventricular zone expressed PH domain homolog 1 (zebrafish)	1.40844
	Hs.289052	233110_s_at	BCL2L12	BCL2-like 12 (proline rich)	1.4093
	Hs.80305	212738_at	ARHGAP19	Rho GTPase activating protein 19	1.41165
	Hs.591308	1553810_a_at	KIAA1524	KIAA1524	1.41457
	Hs.526902	226989_at	RGMB	RGM domain family, member B	1.41505
	Hs.631886	206036_s_at	REL	v-rel reticuloendotheliosis viral oncogene homolog (avian)	1.4154
	Hs.643599	224941_at	PAPPA	pregnancy-associated plasma protein A, pappalysin 1	1.41542
	Hs.284491	202671_s_at	PDXK	pyridoxal (pyridoxine, vitamin B6) kinase	1.41633
	Hs.656047	1554283_at	CCRN4L	CCR4 carbon catabolite repression 4-like (S. cerevisiae)	1.41667
	Hs.153479	204817_at	ESPL1	extra spindle pole bodies homolog 1 (S. cerevisiae)	1.41735
	Hs.595391	1556601_a_at	SPATA13	Spermatogenesis associated 13	1.41912
	Hs.280387	228955_at	LRP8	low density lipoprotein receptor-related protein 8, apolipoprotein e receptor	1.41976
	Hs.370834	222740_at	ATAD2	ATPase family, AAA domain containing 2	1.42077
	Hs.277035	211026_s_at	MGLL	monoglyceride lipase	1.42133
	Hs.23642	205449_at	SAC3D1	SAC3 domain containing 1	1.42263
	Hs.607822	212022_s_at	MKI67	antigen identified by monoclonal antibody Ki-67	1.42439
	Hs.122908	228868_x_at	CDT1	Chromatin licensing and DNA replication factor 1	1.42525
	Hs.83169	204475_at	MMP1	matrix metallopeptidase 1 (interstitial collagenase)	1.42643
	Hs.177926	208107_s_at	LOC81691	exonuclease NEF-sp	1.42792
	Hs.477015	223395_at	ABI3BP	ABI family, member 3 (NESH) binding protein	1.42956
	Hs.130098	201440_at	DDX23	DEAD (Asp-Glu-Ala-Asp) box polypeptide 23	1.43051
	Hs.596464	231810_at	BRI3BP	BRI3 binding protein	1.43254
	Hs.532968	218726_at	HJURP	Holliday junction recognition protein	1.43317
	Hs.135094	222039_at	KIF18B	kinesin family member 18B	1.43381
	Hs.369998	238508_at	DBF4B	DBF4 homolog B (S. cerevisiae)	1.43844
	Hs.517617	205193_at	MAFF	v-maf musculoaponeurotic fibrosarcoma oncogene homolog F (avian)	1.4405
	Hs.215766	228002_at	GTPBP4 /// IDI2	GTP binding protein 4 /// isopentenyl-diphosphate delta isomerase 2	1.44074
	Hs.224607	201286_at	SDC1	syndecan 1	1.44096
	Hs.86368	205830_at	CLGN	calmegin	1.44125
	Hs.518450	202390_s_at	HTT	huntingtin	1.44196
	Hs.374201	226003_at	KIF21A	kinesin family member 21A	1.44262
	Hs.595793	39549_at	NPAS2	neuronal PAS domain protein 2	1.44263
	Hs.526902	227339_at	RGMB	RGM domain family, member B	1.44271
	Hs.526464	211013_x_at	PML	promyelocytic leukemia	1.44618
	Hs.533273	200964_at	UBA1	ubiquitin-like modifier activating enzyme 1	1.45496
	Hs.103755	209544_at	RIPK2	receptor-interacting serine-threonine kinase 2	1.45516
	Hs.517033	211003_x_at	TGM2	transglutaminase 2 (C polypeptide, protein-glutamine-gamma-glutamyltransferase)	1.45885

	Hs.375684	238670_at	RAD18	RAD18 homolog (S. cerevisiae)	1.45961
	Hs.396393	202779_s_at	UBE2S	ubiquitin-conjugating enzyme E2S	1.46009
	Hs.720829	218296_x_at	MSTO1 /// MSTO2P	misato homolog 1 (Drosophila) /// misato homolog 2 pseudogene	1.46111
	Hs.81892	211713_x_at	KIAA0101	KIAA0101	1.46199
	Hs.248815	241937_s_at	WDR4	WD repeat domain 4	1.46276
	Hs.597057	236243_at	ZCCHC6	Zinc finger, CCHC domain containing 6	1.46392
	Hs.489615	217739_s_at	NAMPT	nicotinamide phosphoribosyltransferase	1.46517
	Hs.2057	202706_s_at	UMPS	uridine monophosphate synthetase	1.46551
	Hs.1973	204826_at	CCNF	cyclin F	1.46623
	Hs.720061	209408_at	KIF2C	kinesin family member 2C	1.4663
	Hs.436446	202655_at	MANF	mesencephalic astrocyte-derived neurotrophic factor	1.47114
	Hs.436912	209680_s_at	KIFC1	kinesin family member C1	1.47379
	Hs.567319	204835_at	POLA1	polymerase (DNA directed), alpha 1, catalytic subunit	1.47536
	Hs.517582	201755_at	MCM5	minichromosome maintenance complex component 5	1.47749
	Hs.410596	236134_at	DCAF7	DDB1 and CUL4 associated factor 7	1.48061
	Hs.303787	230150_at	BCAP29	B-cell receptor-associated protein 29	1.48083
	Hs.517617	36711_at	MAFF	v-maf musculoaponeurotic fibrosarcoma oncogene homolog F (avian)	1.48203
	Hs.198612	209990_s_at	GABBR2	gamma-aminobutyric acid (GABA) B receptor, 2	1.48224
	Hs.368912	211478_s_at	DPP4	dipeptidyl-peptidase 4	1.48538
	Hs.390736	210564_x_at	CFLAR	CASP8 and FADD-like apoptosis regulator	1.48647
	Hs.526902	242450_at	RGMB	RGM domain family, member B	1.48785
	Hs.740700	220346_at	MTHFD2L	methylenetetrahydrofolate dehydrogenase (NADP+ dependent) 2-like	1.49159
	Hs.494557	229551_x_at	ZNF367	zinc finger protein 367	1.49551
	Hs.591087	231041_at	POLR1E	polymerase (RNA) I polypeptide E, 53kDa	1.49871
	Hs.736548	1565786_x_at	FLJ45482	uncharacterized LOC645566	1.49985
	Hs.442609	225103_at	MRPL38	mitochondrial ribosomal protein L38	1.5006
	Hs.567229	207071_s_at	ACO1	aconitase 1, soluble	1.50164
	Hs.390736	209939_x_at	CFLAR	CASP8 and FADD-like apoptosis regulator	1.50613
	Hs.458922	219066_at	PPCDC	phosphopantothenoylcysteine decarboxylase	1.50975
	Hs.595793	213462_at	NPAS2	neuronal PAS domain protein 2	1.51018
	Hs.513126	213008_at	FANCI	Fanconi anemia, complementation group I	1.51268
	Hs.730607	205086_s_at	NCAPH2	non-SMC condensin II complex, subunit H2	1.51343
	Hs.514843	232030_at	EPG5	ectopic P-granules autophagy protein 5 homolog (C. elegans)	1.51504
	Hs.99004	241749_at	MURC	muscle-related coiled-coil protein	1.51721
	Hs.482291	237411_at	ADAMTS6	ADAM metallopeptidase with thrombospondin type 1 motif, 6	1.51847
	Hs.353773	1558847_at	LINC00565	long intergenic non-protein coding RNA 565	1.52139
	Hs.591054	211725_s_at	BID	BH3 interacting domain death agonist	1.52781
	Hs.71827	209567_at	RRS1	RRS1 ribosome biogenesis regulator homolog (S. cerevisiae)	1.52828
	Hs.460184	212141_at	MCM4	minichromosome maintenance complex component 4	1.5288
	Hs.303116	218681_s_at	SDF2L1	stromal cell-derived factor 2-like 1	1.52973
	Hs.741061	228990_at	SNHG12 /// SNORA16A /// SNORA44 /// SNORA61	small nucleolar RNA host gene 12 (non-protein coding) /// small nucleolar RNA, H/ACA bo	1.53017
	Hs.655830	223556_at	HELLS	helicase, lymphoid-specific	1.53311
	Hs.95008	205563_at	KISS1	KiSS-1 metastasis-suppressor	1.53509
	Hs.280387	208433_s_at	LRP8	low density lipoprotein receptor-related protein 8, apolipoprotein e receptor	1.53606
	Hs.725208	205733_at	BLM	Bloom syndrome, RecQ helicase-like	1.53609
	Hs.501928	236475_at	MICAL2	Microtubule associated monoxygenase, calponin and LIM domain containing 2	1.53738
	Hs.568818	225167_at	FRMD4A	FERM domain containing 4A	1.53761
	Hs.444947	202241_at	TRIB1	tribbles homolog 1 (Drosophila)	1.54096
	Hs.729825	217553_at	STEAP1B	STEAP family member 1B	1.54113
	Hs.654542	205870_at	BDKRB2	bradykinin receptor B2	1.5416
	Hs.643599	201981_at	PAPPA	pregnancy-associated plasma protein A, pappalysin 1	1.54341

	Hs.483793	226955_at	AFAP1L1	actin filament associated protein 1-like 1	1.55501
	Hs.505575	228303_at	GALNT6	UDP-N-acetyl-alpha-D-galactosamine:polypeptide N-acetylgalactosaminyltransferase 6 (Gal	1.55698
	Hs.489615	217738_at	NAMPT	nicotinamide phosphoribosyltransferase	1.5582
	Hs.195080	201749_at	ECE1	endothelin converting enzyme 1	1.55827
	Hs.434886	224753_at	CDCA5	cell division cycle associated 5	1.56143
	Hs.481720	244350_at	MYO10	myosin X	1.56274
	Hs.740401	210334_x_at	BIRC5	baculoviral IAP repeat containing 5	1.57067
	Hs.434247	219474_at	C3orf52	chromosome 3 open reading frame 52	1.57357
	Hs.523252	225582_at	ITPRIP	inositol 1,4,5-trisphosphate receptor interacting protein	1.57567
	Hs.127473	244552_at	ZNF788	zinc finger family member 788	1.57707
	Hs.465643	227420_at	TNFAIP8L1	tumor necrosis factor, alpha-induced protein 8-like 1	1.58015
	Hs.319334	201969_at	NASP	nuclear autoantigenic sperm protein (histone-binding)	1.58102
	Hs.521693	205034_at	CCNE2	cyclin E2	1.58161
	Hs.591642	205345_at	BARD1	BRCA1 associated RING domain 1	1.58908
	Hs.546467	235276_at	EPSTI1	epithelial stromal interaction 1 (breast)	1.58959
	Hs.355141	243423_at	TNIP1	TNFAIP3 interacting protein 1	1.59095
	Hs.567488	219920_s_at	AMIGO3 /// GMPPB	adhesion molecule with Ig-like domain 3 /// GDP-mannose pyrophosphorylase B	1.59316
	Hs.201897	204441_s_at	POLA2	polymerase (DNA directed), alpha 2, accessory subunit	1.59615
	Hs.31442	213520_at	RECQL4	RecQ protein-like 4	1.59833
	Hs.466871	210845_s_at	PLAUR	plasminogen activator, urokinase receptor	1.59994
	Hs.128903	235609_at	BRIP1	BRCA1 interacting protein C-terminal helicase 1	1.60048
	Hs.444403	201957_at	PPP1R12B	protein phosphatase 1, regulatory subunit 12B	1.60333
	Hs.193268	231984_at	MTAP	methylthioadenosine phosphorylase	1.60614
	Hs.464438	225059_at	AGTRAP	angiotensin II receptor-associated protein	1.60744
	Hs.268874	241981_at	FAM20A	family with sequence similarity 20, member A	1.61328
	Hs.684549	1566968_at	SPRY4-IT1	SPRY4 intronic transcript 1 (non-protein coding)	1.61955
	Hs.414795	202628_s_at	SERPINE1	serpin peptidase inhibitor, clade E (nexin, plasminogen activator inhibitor type 1), me	1.61957
	Hs.22678	218590_at	C10orf2	chromosome 10 open reading frame 2	1.62153
	Hs.516217	232180_at	UGP2	UDP-glucose pyrophosphorylase 2	1.62774
	Hs.404088	226308_at	HAUS8	HAUS augmin-like complex, subunit 8	1.6305
	Hs.212360	243938_x_at	DNAH5	dynein, axonemal, heavy chain 5	1.63187
	Hs.501890	207992_s_at	AMPD3	adenosine monophosphate deaminase 3	1.63189
	Hs.730686	211139_s_at	NAB1	NGFI-A binding protein 1 (EGR1 binding protein 1)	1.6323
	Hs.522568	218951_s_at	PLCXD1	phosphatidylinositol-specific phospholipase C, X domain containing 1	1.63352
	Hs.655672	206298_at	ARHGAP22	Rho GTPase activating protein 22	1.63711
	Hs.438720	208795_s_at	MCM7	minichromosome maintenance complex component 7	1.63805
	Hs.80976	212023_s_at	MKI67	antigen identified by monoclonal antibody Ki-67	1.6393
	Hs.198363	222962_s_at	MCM10	minichromosome maintenance complex component 10	1.64666
	Hs.476218	204136_at	COL7A1	collagen, type VII, alpha 1	1.65029
	Hs.740377	209387_s_at	TM4SF1	transmembrane 4 L six family member 1	1.65034
	Hs.721963	235758_at	PNMA6A /// PNMA6B /// PNMA6C /// PNMA6D	paraneoplastic Ma antigen family member 6A /// paraneoplastic Ma antigen family member	1.65334
	Hs.466039	202910_s_at	CD97	CD97 molecule	1.65907
	Hs.471200	214632_at	NRP2	neuropilin 2	1.66027
	Hs.226390	201890_at	RRM2	ribonucleotide reductase M2	1.66426
	Hs.34333	243805_at	CCBE1	collagen and calcium binding EGF domains 1	1.66435
	Hs.607822	212021_s_at	MKI67	antigen identified by monoclonal antibody Ki-67	1.6657
	Hs.50640	210001_s_at	SOCS1	suppressor of cytokine signaling 1	1.67331
	Hs.405958	203967_at	CDC6	cell division cycle 6 homolog (S. cerevisiae)	1.68253
	Hs.409065	204767_s_at	FEN1	flap structure-specific endonuclease 1	1.68277
	Hs.201398	220975_s_at	C1QTNF1	C1q and tumor necrosis factor related protein 1	1.68414

	Hs.268874	242945_at	FAM20A	family with sequence similarity 20, member A	1.69493
	Hs.442658	209464_at	AURKB	aurora kinase B	1.71085
	Hs.129944	208394_x_at	ESM1	endothelial cell-specific molecule 1	1.71591
	Hs.61635	205542_at	STEAP1	six transmembrane epithelial antigen of the prostate 1	1.71788
	Hs.438231	209277_at	TFPI2	tissue factor pathway inhibitor 2	1.71929
	Hs.470654	224428_s_at	CDCA7	cell division cycle associated 7	1.72155
	Hs.740377	215034_s_at	TM4SF1	transmembrane 4 L six family member 1	1.72548
	Hs.80642	206118_at	STAT4	signal transducer and activator of transcription 4	1.72734
	Hs.181855	1552682_a_at	CASC5	cancer susceptibility candidate 5	1.73896
	Hs.55999	209706_at	NKX3-1	NK3 homeobox 1	1.74147
	Hs.474217	204126_s_at	CDC45	cell division cycle 45 homolog (S. cerevisiae)	1.75848
	Hs.405958	203968_s_at	CDC6	cell division cycle 6 homolog (S. cerevisiae)	1.76045
	Hs.75514	201695_s_at	PNP	purine nucleoside phosphorylase	1.76326
	Hs.195155	234973_at	SLC38A5	solute carrier family 38, member 5	1.76363
	Hs.568818	225163_at	FRMD4A	FERM domain containing 4A	1.76518
	Hs.355141	207196_s_at	TNIP1	TNFAIP3 interacting protein 1	1.76818
	Hs.65758	201189_s_at	ITPR3	inositol 1,4,5-trisphosphate receptor, type 3	1.7691
	Hs.409065	204768_s_at	FEN1	flap structure-specific endonuclease 1	1.7727
	Hs.731569	226118_at	CENPO	centromere protein O	1.77328
	Hs.523526	219990_at	E2F8	E2F transcription factor 8	1.78243
	Hs.248114	230090_at	GDNF	glial cell derived neurotrophic factor	1.78468
	Hs.654557	215966_x_at	GK3P	glycerol kinase 3 pseudogene	1.79927
	Hs.740377	209386_at	TM4SF1	transmembrane 4 L six family member 1	1.80327
	Hs.272011	201883_s_at	B4GALT1	UDP-Gal:betaGlcNAc beta 1,4- galactosyltransferase, polypeptide 1	1.81654
	Hs.495710	209170_s_at	GPM6B	glycoprotein M6B	1.82187
	Hs.477481	202107_s_at	MCM2	minichromosome maintenance complex component 2	1.8372
	Hs.302903	233360_at	UBE2I	ubiquitin-conjugating enzyme E2I	1.83785
	Hs.19322	225777_at	SAPCD2	suppressor APC domain containing 2	1.84473
	Hs.446091	210285_x_at	WTAP	Wilms tumor 1 associated protein	1.84965
	Hs.210995	204508_s_at	CA12	carbonic anhydrase XII	1.8534
	Hs.80976	212020_s_at	MKI67	antigen identified by monoclonal antibody Ki-67	1.86065
	Hs.656573	1560031_at	FRMD4A	FERM domain containing 4A	1.86617
	Hs.482291	1570351_at	ADAMTS6	ADAM metallopeptidase with thrombospondin type 1 motif, 6	1.8766
	Hs.647370	217546_at	MT1M	metallothionein 1M	1.88875
	Hs.575032	229304_s_at	MLF1IP	MLF1 interacting protein	1.89673
	Hs.499952	227804_at	TLCD1	TLC domain containing 1	1.91325
	Hs.708195	227125_at	IFNAR2	interferon (alpha, beta and omega) receptor 2	1.91877
	Hs.708195	204786_s_at	IFNAR2	interferon (alpha, beta and omega) receptor 2	1.92091
	Hs.583348	227140_at	INHBA	inhibin, beta A	1.92415
	Hs.432132	213524_s_at	G0S2	G0/G1switch 2	1.93032
	Hs.642042	204558_at	RAD54L	RAD54-like (S. cerevisiae)	1.94394
	Hs.440829	213006_at	CEBPD	CCAAT/enhancer binding protein (C/EBP), delta	1.95973
	Hs.476306	226355_at	POC1A	POC1 centriolar protein homolog A (Chlamydomonas)	1.9662
	Hs.521989	227458_at	CD274	CD274 molecule	2.00191
	Hs.369265	213817_at	IRAK3	interleukin-1 receptor-associated kinase 3	2.00363
	Hs.521212	201272_at	AKR1B1	aldo-keto reductase family 1, member B1 (aldose reductase)	2.00491
	Hs.591742	226218_at	IL7R	interleukin 7 receptor	2.04689
	Hs.731427	213112_s_at	SQSTM1	sequestosome 1	2.05908
	Hs.458276	203927_at	NFKBIE	nuclear factor of kappa light polypeptide gene enhancer in B-cells inhibitor, epsilon	2.06227
	Hs.259599	233002_at	PPP4R4	protein phosphatase 4, regulatory subunit 4	2.06663
	Hs.730686	209272_at	NAB1	NGFI-A binding protein 1 (EGR1 binding protein 1)	2.09081
	Hs.656436	213712_at	ELOVL2	ELOVL fatty acid elongase 2	2.10373
	Hs.159226	230372_at	HAS2	hyaluronan synthase 2	2.11046

	Hs.143250	216005_at	TNC	Tenascin C	2.16162
	Hs.583348	210511_s_at	INHBA	inhibin, beta A	2.24872
	Hs.445447	220658_s_at	ARNTL2	aryl hydrocarbon receptor nuclear translocator-like 2	2.24876
	Hs.740403	213338_at	TMEM158	transmembrane protein 158 (gene/pseudogene)	2.2642
	Hs.511251	217995_at	SQRDL	sulfide quinone reductase-like (yeast)	2.26974
	Hs.159226	206432_at	HAS2	hyaluronan synthase 2	2.29456
	Hs.505545	203123_s_at	SLC11A2	solute carrier family 11 (proton-coupled divalent metal ion transporters), member 2	2.35232
	Hs.374191	218717_s_at	LEPREL1	leprecan-like 1	2.38342
	Hs.256278	203508_at	TNFRSF1B	tumor necrosis factor receptor superfamily, member 1B	2.41406
	Hs.372578	227654_at	FAM65C	family with sequence similarity 65, member C	2.42588
	Hs.376289	231899_at	ZC3H12C	zinc finger CCCH-type containing 12C	2.42593
	Hs.404741	204702_s_at	NFE2L3	nuclear factor (erythroid-derived 2)-like 3	2.43735
	Hs.143674	205801_s_at	RASGRP3	RAS guanyl releasing protein 3 (calcium and DAG-regulated)	2.4456
	Hs.244378	220091_at	SLC2A6	solute carrier family 2 (facilitated glucose transporter), member 6	2.4753
	Hs.76095	201631_s_at	IER3	immediate early response 3	2.48042
	Hs.437322	206026_s_at	TNFAIP6	tumor necrosis factor, alpha-induced protein 6	2.49373
	Hs.186649	213142_x_at	PION	pigeon homolog (Drosophila)	2.63161
	Hs.491232	212110_at	SLC39A14	solute carrier family 39 (zinc transporter), member 14	2.66456
	Hs.196384	1554997_a_at	PTGS2	prostaglandin-endoperoxide synthase 2 (prostaglandin G/H synthase and cyclooxygenase)	2.70371
	Hs.525607	202510_s_at	TNFAIP2	tumor necrosis factor, alpha-induced protein 2	2.74942
	Hs.584921	1567080_s_at	CLN6	ceroid-lipofuscinosis, neuronal 6, late infantile, variant	2.76809
	Hs.442619	209928_s_at	MSC	musculin	2.77444
	Hs.25590	204597_x_at	STC1	stanniocalcin 1	2.82574
	Hs.319171	223218_s_at	NFKBIZ	nuclear factor of kappa light polypeptide gene enhancer in B-cells inhibitor, zeta	2.85356
	Hs.437322	206025_s_at	TNFAIP6	tumor necrosis factor, alpha-induced protein 6	2.90676
	Hs.25590	204595_s_at	STC1	stanniocalcin 1	3.06913
	Hs.3781	209840_s_at	LRRN3	leucine rich repeat neuronal 3	3.07459
	Hs.477015	220518_at	ABI3BP	ABI family, member 3 (NESH) binding protein	3.15721
	Hs.381072	201490_s_at	PPIF	peptidylprolyl isomerase F	3.24199
	Hs.81328	201502_s_at	NFKBIA	nuclear factor of kappa light polypeptide gene enhancer in B-cells inhibitor, alpha	3.28789
	Hs.530443	227099_s_at	C11orf96	chromosome 11 open reading frame 96	3.47496
	Hs.186649	222150_s_at	PION	pigeon homolog (Drosophila)	3.59461
	Hs.122908	209832_s_at	CDT1	chromatin licensing and DNA replication factor 1	3.65197
	Hs.86724	204224_s_at	GCH1	GTP cyclohydrolase 1	3.93666
	Hs.654402	205205_at	RELB	v-rel reticuloendotheliosis viral oncogene homolog B	4.11439
	Hs.181301	202902_s_at	CTSS	cathepsin S	4.49863
	Hs.468675	221898_at	PDPN	podoplanin	4.92404
	Hs.127799	210538_s_at	BIRC3	baculoviral IAP repeat containing 3	5.20979
	Hs.211600	202644_s_at	TNFAIP3	tumor necrosis factor, alpha-induced protein 3	5.55381
	Hs.439060	222549_at	CLDN1	claudin 1	5.5729
	Hs.487046	216841_s_at	LOC100129518 /// SOD2	uncharacterized LOC100129518 /// superoxide dismutase 2, mitochondrial	5.809
	Hs.487046	221477_s_at	LOC100129518 /// SOD2	uncharacterized LOC100129518 /// superoxide dismutase 2, mitochondrial	6.85487
	Hs.375129	205828_at	MMP3	matrix metallopeptidase 3 (stromelysin 1, progelatinase)	7.15274
	Hs.303649	216598_s_at	CCL2	chemokine (C-C motif) ligand 2	7.18404
	Hs.211600	202643_s_at	TNFAIP3	tumor necrosis factor, alpha-induced protein 3	9.17506
	Hs.69771	202357_s_at	CFB	complement factor B	9.37489
	Hs.487046	215223_s_at	LOC100129518 /// SOD2	uncharacterized LOC100129518 /// superoxide dismutase 2, mitochondrial	9.75648
	Hs.288034	209267_s_at	SLC39A8	solute carrier family 39 (zinc transporter), member 8	13.0218
	Hs.529053	217767_at	C3	complement component 3	13.2918
	Hs.643447	202637_s_at	ICAM1	intercellular adhesion molecule 1	14.9248
	Hs.654458	205207_at	IL6	interleukin 6 (interferon, beta 2)	17.3638

	Hs.89714	214974_x_at	CXCL5	chemokine (C-X-C motif) ligand 5	22.1835
	Hs.164021	206336_at	CXCL6	chemokine (C-X-C motif) ligand 6 (granulocyte chemotactic protein 2)	22.9749
	Hs.643447	202638_s_at	ICAM1	intercellular adhesion molecule 1	30.7033
	Hs.789	204470_at	CXCL1	chemokine (C-X-C motif) ligand 1 (melanoma growth stimulating activity, alpha)	33.0198
	Hs.624	202859_x_at	IL8	interleukin 8	41.0083
	Hs.89690	207850_at	CXCL3	chemokine (C-X-C motif) ligand 3	50.0736
	Hs.75765	209774_x_at	CXCL2	chemokine (C-X-C motif) ligand 2	52.0387
	Hs.112242	223484_at	C15orf48	chromosome 15 open reading frame 48	60.4786
	Hs.624	211506_s_at	IL8	interleukin 8	114.641

Supplemental Table 3. Genes differentially expressed in primed stromal cells compared with unprimed stromal cells. Green line indicates genes overexpressed in unprimed stromal cells and red line indicates genes overexpressed in primed stromal cells. Fold Change corresponds to the ratio of median expression in PMN-primed / unprimed stroma.

UniGene ID	Probeset ID	Gene Symbol	Gene Title	FoldChange
Hs,280781	220301_at	CCDC102B	coiled-coil domain containing 102B	-3,57953
Hs,406013	201596_x_at	KRT18	keratin 18	-2,96748
Hs,471610	219636_s_at	ARMC9	armadillo repeat containing 9	-2,12238
Hs,102735	238861_at	SSBP2	single-stranded DNA binding protein 2	-1,83589
Hs,486508	244353_s_at	SLC2A12	solute carrier family 2 (facilitated gl	-1,82481
Hs,655519	227662_at	SYNPO2	synaptopodin 2	-1,80744
Hs,234478	228503_at	RPS6KA6	ribosomal protein S6 kinase, 90kDa, pol	-1,80272
Hs,156727	223092_at	ANKH	ankylosis, progressive homolog (mouse)	-1,79367
Hs,714330	206481_s_at	LDB2	LIM domain binding 2	-1,77008
Hs,531005	241359_at	TLCD2	TLC domain containing 2	-1,66124
Hs,632079	1568598_at	KAZALD1	Kazal-type serine peptidase inhibitor d	-1,6485
Hs,40510	1552774_a_at	SLC25A27	solute carrier family 25, member 27	-1,64698
Hs,293798	226113_at	ZNF436	zinc finger protein 436	-1,64313
Hs,592184	203106_s_at	VPS41	vacuolar protein sorting 41 homolog (S,	-1,63009
Hs,728944	207417_s_at	ZNF177 /// ZNF559-ZNF177	zinc finger protein 177 /// ZNF559-ZNF1	-1,62344
Hs,102735	210829_s_at	SSBP2	single-stranded DNA binding protein 2	-1,61799
Hs,108049	223279_s_at	UACA	uveal autoantigen with coiled-coil doma	-1,61589
Hs,716678	244050_at	PTPLAD2	protein tyrosine phosphatase-like A dom	-1,61411
Hs,435458	227478_at	SETBP1	SET binding protein 1	-1,60415
Hs,582993	231175_at	BEND6	BEN domain containing 6	-1,598
Hs,567679	1552370_at	C4orf33	chromosome 4 open reading frame 33	-1,58144
Hs,503297	32502_at	GDPD5	glycerophosphodiester phosphodiesterase	-1,54544
Hs,360174	213139_at	SNAI2	snail homolog 2 (Drosophila)	-1,54497
Hs,192586	227522_at	CMBL	carboxymethylenebutenolidase homolog (P	-1,53599
Hs,709356	210306_at	L3MBTL1	l(3)mbt-like 1 (Drosophila)	-1,53364
Hs,460857	227889_at	LPCAT2	lysophosphatidylcholine acyltransferase	-1,52782
Hs,494997	205500_at	C5	complement component 5	-1,52696
Hs,638960 /// Hs,732670	1562876_s_at	LOC541471	Uncharacterized LOC541471	-1,52229
Hs,369201	238127_at	GAS6-AS1	GAS6 antisense RNA 1 (non-protein codin	-1,5141
Hs,301526	242056_at	TRIM45	tripartite motif containing 45	-1,51382
Hs,666807	240429_at	ZNF546	zinc finger protein 546	-1,50975
Hs,102735	203787_at	SSBP2	single-stranded DNA binding protein 2	-1,50709
Hs,592549	213685_at	LOC100506963	uncharacterized LOC100506963	-1,50671
Hs,183983	230076_at	PITPNM3	PITPNM family member 3	-1,48763
Hs,125056	219696_at	DENND1B	DENN/MADD domain containing 1B	-1,48712
Hs,125056	228032_s_at	DENND1B	DENN/MADD domain containing 1B	-1,48609
Hs,84928	218128_at	NFYB	nuclear transcription factor Y, beta	-1,4851
Hs,446946	230121_at	C1orf133	chromosome 1 open reading frame 133	-1,47353
Hs,309489	227001_at	NIPAL2	NIPA-like domain containing 2	-1,46684
Hs,435458	205933_at	SETBP1	SET binding protein 1	-1,46545
Hs,154163	221564_at	PRMT2	protein arginine methyltransferase 2	-1,45545
Hs,132225	212239_at	PIK3R1	phosphoinositide-3-kinase, regulatory s	-1,45164
Hs,335034	204646_at	DPYD	dihydropyrimidine dehydrogenase	-1,45099
Hs,529551	223444_at	SENP7	SUMO1/sentrin specific peptidase 7	-1,44364
Hs,105134	229498_at	MBNL3	muscleblind-like splicing regulator 3	-1,44093
Hs,436142	204201_s_at	PTPN13	protein tyrosine phosphatase, non-recep	-1,42724
---	229190_at	LOC100507376	uncharacterized LOC100507376	-1,42691

	Hs,330073	203288_at	KIAA0355	KIAA0355	-1,42667
	Hs,518927	1569713_at	SEC24B-AS1	SEC24B antisense RNA 1 (non-protein cod	-1,4256
	Hs,12326	236076_at	LOC257396	uncharacterized LOC257396	-1,42412
	Hs,533055	203845_at	KAT2B	K(lysine) acetyltransferase 2B	-1,42032
	Hs,484195	235556_at	CREBRF	CREB3 regulatory factor	-1,41799
	Hs,600086	212936_at	FAM172A	family with sequence similarity 172, me	-1,39589
	Hs,293970	221589_s_at	ALDH6A1	aldehyde dehydrogenase 6 family, member	-1,39358
	Hs,11637	226995_at	LOC642852	uncharacterized LOC642852	-1,39208
	Hs,497492	225742_at	MDM4	Mdm4 p53 binding protein homolog (mouse	-1,38646
	Hs,209151	225469_at	LYRM5	LYR motif containing 5	-1,38503
	Hs,484195	225956_at	CREBRF	CREB3 regulatory factor	-1,37818
	Hs,498892	225545_at	EEF2K	eukaryotic elongation factor-2 kinase	-1,37385
	Hs,731467	228791_at	LOC100129502	uncharacterized LOC100129502	-1,37314
	Hs,731699	212665_at	TIPARP	TCDD-inducible poly(ADP-ribose) polymer	-1,36782
	Hs,577252	226169_at	SBF2	SET binding factor 2	-1,36648
	Hs,482868	203049_s_at	TTC37	tetratricopeptide repeat domain 37	-1,36558
	Hs,593422	1555037_a_at	IDH1	isocitrate dehydrogenase 1 (NADP+), sol	-1,36366
	Hs,554791	203421_at	TP53I11	tumor protein p53 inducible protein 11	-1,36094
	Hs,201858	201152_s_at	MBNL1	muscleblind-like splicing regulator 1	-1,36094
	Hs,397729	221750_at	HMGCS1	3-hydroxy-3-methylglutaryl-CoA synthase	-1,3587
	Hs,425769	226019_at	OMA1	OMA1 zinc metallopeptidase homolog (S,	-1,35833
	Hs,212332	203324_s_at	CAV2	caveolin 2	-1,35632
	Hs,409210	207232_s_at	DZIP3	DAZ interacting protein 3, zinc finger	-1,35359
	Hs,477375	1569956_at	MYLK	myosin light chain kinase	-1,35312
	Hs,497253	219757_s_at	C14orf101	chromosome 14 open reading frame 101	-1,35204
	Hs,483239	208950_s_at	ALDH7A1	aldehyde dehydrogenase 7 family, member	-1,35189
	Hs,84928	218127_at	NFYB	nuclear transcription factor Y, beta	-1,35003
	Hs,586279	227866_at	LOC100505519 /// TIAM2	uncharacterized LOC100505519 /// T-cell	-1,34958
	Hs,122927	226425_at	CLIP4	CAP-GLY domain containing linker protei	-1,34931
	Hs,485865	206006_s_at	KIAA1009	KIAA1009	-1,34251
	Hs,408577	213463_s_at	FAM149B1	family with sequence similarity 149, me	-1,34223
	Hs,422986	201302_at	ANXA4	annexin A4	-1,3391
	Hs,29173	51192_at	SSH3	slingshot homolog 3 (Drosophila)	-1,33794
	Hs,149252	204920_at	CPS1	carbamoyl-phosphate synthase 1, mitocho	-1,33712
	Hs,516159	65472_at	C2orf68	chromosome 2 open reading frame 68	-1,33711
	Hs,403594	238458_at	EFHA2	EF-hand domain family, member A2	-1,33623
	Hs,496139	219026_s_at	RASAL2	RAS protein activator like 2	-1,3346
	Hs,509414	214709_s_at	KTN1	kinectin 1 (kinesin receptor)	-1,33399
	Hs,142245	220387_s_at	HHLA3	HERV-H LTR-associating 3	-1,3337
	Hs,718875	203724_s_at	RUFY3	RUN and FYVE domain containing 3	-1,33242
	Hs,555902	206414_s_at	ASAP2	ArfGAP with SH3 domain, ankyrin repeat	-1,33153
	---	228718_at	ZNF44	zinc finger protein 44	-1,32898
	Hs,506558	219484_at	HCFC2	host cell factor C2	-1,32851
	Hs,59504	228174_at	SCAI	suppressor of cancer cell invasion	-1,32851
	Hs,21938	218047_at	OSBPL9	oxysterol binding protein-like 9	-1,32648
	Hs,408461	204909_at	DDX6	DEAD (Asp-Glu-Ala-Asp) box helicase 6	-1,3259
	Hs,483239	208951_at	ALDH7A1	aldehyde dehydrogenase 7 family, member	-1,32447
	Hs,11637	243656_at	LOC642852	uncharacterized LOC642852	-1,32397
	Hs,88297	226525_at	STK17B	serine/threonine kinase 17b	-1,32277
	Hs,650158	225295_at	SLC39A10	solute carrier family 39 (zinc transpor	-1,32137
	Hs,524809	210716_s_at	CLIP1	CAP-GLY domain containing linker protei	-1,32029
	Hs,475872	214436_at	FBXL2	F-box and leucine-rich repeat protein 2	-1,32009

	Hs,512180	227823_at	RGAG4	retrotransposon gag domain containing 4	-1,3176
	Hs,711490	212593_s_at	MIR4680 /// PDCD4	microRNA 4680 /// programmed cell death	-1,31721
	Hs,306691	244128_x_at	GLIS1	GLIS family zinc finger 1	-1,31651
	Hs,593422	201193_at	IDH1	isocitrate dehydrogenase 1 (NADP+), sol	-1,31555
	Hs,335139	217894_at	KCTD3	potassium channel tetramerisation domai	-1,31494
	Hs,293970	221588_x_at	ALDH6A1	aldehyde dehydrogenase 6 family, member	-1,31348
	Hs,546711	1559950_at	FAM66C /// FAM66D	family with sequence similarity 66, mem	-1,3133
	Hs,149387	203216_s_at	MYO6	myosin VI	-1,31294
	Hs,699548	241393_at	IPP	intracisternal A particle-promoted poly	-1,31201
	Hs,486434	227572_at	USP30	ubiquitin specific peptidase 30	-1,3107
	Hs,502756	220016_at	AHNAK	AHNAK nucleoprotein	-1,30886
	Hs,719172	205571_at	LIPT1	lipoyltransferase 1	-1,30819
	Hs,497575	227649_s_at	SRGAP2	SLIT-ROBO Rho GTPase activating protein	-1,30819
	Hs,422986	201301_s_at	ANXA4	annexin A4	-1,30799
	Hs,485784	202319_at	SENP6	SUMO1/sentrin specific peptidase 6	-1,30647
	Hs,521800	223214_s_at	ZHX1	zinc fingers and homeoboxes 1	-1,30451
	Hs,525518	234929_s_at	SPATA7	spermatogenesis associated 7	-1,30398
	Hs,731687	221895_at	MOSPD2	motile sperm domain containing 2	-1,30315
	Hs,47382	225373_at	C10orf54	chromosome 10 open reading frame 54	-1,30236
	Hs,661604	1563498_s_at	SLC25A45	solute carrier family 25, member 45	-1,30187
	Hs,409210	213186_at	DZIP3	DAZ interacting protein 3, zinc finger	-1,30118
	Hs,5009	225341_at	MTERFD3	MTERF domain containing 3	-1,2994
	Hs,732093	219029_at	C5orf28	chromosome 5 open reading frame 28	-1,29777
	Hs,165762	228220_at	FCHO2	FCH domain only 2	-1,29624
	Hs,15243	36030_at	IFFO1	intermediate filament family orphan 1	-1,29593
	Hs,94896	218477_at	TMEM14A	transmembrane protein 14A	-1,29409
	Hs,711490	212594_at	MIR4680 /// PDCD4	microRNA 4680 /// programmed cell death	-1,29405
	Hs,425769	226020_s_at	DAB1 /// OMA1	disabled homolog 1 (Drosophila) /// OMA	-1,28754
	Hs,127675	229958_at	CLN8	ceroid-lipofuscinosis, neuronal 8 (epil	-1,28612
	Hs,568986	228882_at	TUB	tubby homolog (mouse)	-1,28418
	Hs,445129	218614_at	C12orf35	chromosome 12 open reading frame 35	-1,28333
	Hs,79101	208796_s_at	CCNG1	cyclin G1	-1,28263
	Hs,591692	228536_at	PRMT10	protein arginine methyltransferase 10 (	-1,2821
	Hs,709591	205087_at	RWDD3	RWD domain containing 3	-1,28183
	Hs,292986	226126_at	TBCK	TBC1 domain containing kinase	-1,28068
	Hs,15243	209721_s_at	IFFO1	intermediate filament family orphan 1	-1,28003
	Hs,731687	64883_at	MOSPD2	motile sperm domain containing 2	-1,27939
	Hs,477475	59631_at	TXNRD3 /// TXNRD3NB	thioredoxin reductase 3 /// thioredoxin	-1,27662
	Hs,509447	229394_s_at	ARHGAP35	Rho GTPase activating protein 35	-1,27506
	Hs,567367	225885_at	EEA1	early endosome antigen 1	-1,26699
	Hs,268939	228012_at	MATR3 /// SNHG4	matrin 3 /// small nucleolar RNA host g	-1,26603
	Hs,546711	1559952_x_at	FAM66C /// FAM66D	family with sequence similarity 66, mem	-1,26501
	Hs,648770	232794_at	LOC153682	Uncharacterized LOC153682	-1,26424
	Hs,78060	202739_s_at	PHKB	phosphorylase kinase, beta	-1,26305
	Hs,157078	221826_at	ANGEL2	angel homolog 2 (Drosophila)	-1,26218
	Hs,740530	218537_at	HCFC1R1	host cell factor C1 regulator 1 (XPO1 d	-1,26184
	Hs,605388	212851_at	DCUN1D4	DCN1, defective in cullin neddylation 1	-1,25933
	Hs,306221	241720_at	ZNF326	zinc finger protein 326	-1,25788
	Hs,408142	212779_at	KIAA1109	KIAA1109	-1,25764
	Hs,605775	226493_at	KCTD18	potassium channel tetramerisation domai	-1,25598
	Hs,15106	202562_s_at	C14orf1	chromosome 14 open reading frame 1	-1,25412

Hs,454528	222388_s_at	VPS35	vacuolar protein sorting 35 homolog (S,	-1,25327
Hs,7921	213248_at	LOC730101	uncharacterized LOC730101	-1,25324
Hs,298990	217707_x_at	SMARCA2	SWI/SNF related, matrix associated, act	-1,25242
---	241910_x_at	LOC100507904	uncharacterized LOC100507904	-1,25051
Hs,379548	230029_x_at	UBR3	ubiquitin protein ligase E3 component n	-1,25047
Hs,467740	212276_at	LPIN1	lipin 1	-1,25036
Hs,149367	212633_at	UFL1	UFM1-specific ligase 1	-1,24974
Hs,131887	230893_at	DNAJC21	DnaJ (Hsp40) homolog, subfamily C, memb	-1,24811
Hs,675132	1553193_at	ZNF441	zinc finger protein 441	-1,24761
Hs,414809	204568_at	ATG14	autophagy related 14	-1,24749
Hs,372082	240044_x_at	TNRC6B	Trinucleotide repeat containing 6B	-1,24735
Hs,425144	205076_s_at	MTMR11	myotubularin related protein 11	-1,2471
Hs,503043	203633_at	CPT1A	carnitine palmitoyltransferase 1A (live	-1,24606
Hs,507584	224874_at	POLR1D	polymerase (RNA) I polypeptide D, 16kDa	-1,2449
Hs,405692	218628_at	CCDC53	coiled-coil domain containing 53	-1,24308
Hs,159188	235360_at	PLEKHM3	pleckstrin homology domain containing,	-1,24285
Hs,593995	212761_at	TCF7L2	transcription factor 7-like 2 (T-cell s	-1,24263
Hs,677935	229891_x_at	KIAA1704	KIAA1704	-1,2401
Hs,655189	212637_s_at	WWP1	WW domain containing E3 ubiquitin prote	-1,2392
Hs,496658	1557411_s_at	SLC25A43	solute carrier family 25, member 43	-1,23776
Hs,444446	238148_s_at	ZNF818P	zinc finger protein 818, pseudogene	-1,23577
Hs,525061	208089_s_at	TDRD3	tudor domain containing 3	-1,2344
Hs,731996	228027_at	ARMCX5-GPRASP2 /// GPRASP2	ARMCX5-GPRASP2 readthrough /// G protei	-1,23351
Hs,477475	221906_at	TXNRD3 /// TXNRD3NB	thioredoxin reductase 3 /// thioredoxin	-1,23237
Hs,448979	226894_at	SLC35A3	solute carrier family 35 (UDP-N-acetylg	-1,23054
Hs,434340	227471_at	HACE1	HECT domain and ankyrin repeat containi	-1,22906
Hs,201858	201153_s_at	MBNL1	muscleblind-like splicing regulator 1	-1,22822
Hs,125038	228011_at	FAM92A1	family with sequence similarity 92, mem	-1,22564
Hs,478199	213518_at	PRKCI	protein kinase C, iota	-1,2252
Hs,709416	220233_at	FBXO17 /// SARS2	F-box protein 17 /// seryl-tRNA synthet	-1,22514
Hs,555989	231850_x_at	CEP44	centrosomal protein 44kDa	-1,22327
Hs,396358	218930_s_at	TMEM106B	transmembrane protein 106B	-1,22322
Hs,372309	234947_s_at	FAM204A	family with sequence similarity 204, me	-1,22282
Hs,333738	223227_at	BBS2	Bardet-Biedl syndrome 2	-1,22172
Hs,632272	235057_at	ITCH	itchy E3 ubiquitin protein ligase	-1,22165
Hs,524899	208740_at	SAP18	Sin3A-associated protein, 18kDa	-1,22144
Hs,130746	32088_at	BLZF1	basic leucine zipper nuclear factor 1	-1,22029
Hs,577053	203620_s_at	FCHSD2	FCH and double SH3 domains 2	-1,21932
Hs,115284	227207_x_at	ZNF213	zinc finger protein 213	-1,21871
Hs,74615	203131_at	PDGFRA	platelet-derived growth factor receptor	-1,21848
Hs,655344	242313_at	LOC728730	Uncharacterized LOC728730	-1,21742
Hs,407918	219441_s_at	LRRK1	leucine-rich repeat kinase 1	-1,21635
Hs,437338	217286_s_at	NDRG3	NDRG family member 3	-1,21498
Hs,493808	207839_s_at	TMEM8B	transmembrane protein 8B	-1,21498
Hs,485527	202960_s_at	MUT	methylmalonyl CoA mutase	-1,21471
Hs,656902	202956_at	ARFGEF1	ADP-ribosylation factor guanine nucleot	-1,21334
Hs,592095	206600_s_at	SLC16A5	solute carrier family 16, member 5 (mon	-1,21176
Hs,521800	223213_s_at	ZHX1	zinc fingers and homeoboxes 1	-1,21022
Hs,156928	1556301_at	LOC100287015	Uncharacterized LOC100287015	-1,20909
Hs,602792	219109_at	SPAG16	sperm associated antigen 16	-1,2088
Hs,485910	225264_at	RARS2	arginyl-tRNA synthetase 2, mitochondria	-1,2067

	Hs,47649	218440_at	MCCC1	methylcrotonoyl-CoA carboxylase 1 (alph	-1,20632
	Hs,567828	226721_at	DPY19L4	dpy-19-like 4 (C, elegans)	-1,20559
	Hs,585006	1552698_at	TUBA3FP	tubulin, alpha 3f, pseudogene	-1,20502
	Hs,166551	218588_s_at	FAM114A2	family with sequence similarity 114, me	-1,20471
	Hs,35758	212462_at	KAT6B	K(lysine) acetyltransferase 6B	-1,20339
	Hs,740366	217971_at	LAMTOR3	late endosomal/lysosomal adaptor, MAPK	-1,20318
	Hs,167700	225223_at	SMAD5	SMAD family member 5	-1,20191
	Hs,410889	224952_at	TANC2	tetratricopeptide repeat, ankyrin repea	-1,2018
	Hs,34906	225049_at	BLOC1S2	biogenesis of lysosomal organelles comp	-1,20179
	Hs,410228	210028_s_at	ORC3	origin recognition complex, subunit 3	-1,20131
	Hs,541894	238464_at	ANKRD36 /// ANKRD36C /// LOC100134365	ankyrin repeat domain 36 /// ankyrin re	-1,20073
	Hs,256747	206816_s_at	SPAG8	sperm associated antigen 8	-1,20057
	Hs,159118	201196_s_at	AMD1	adenosylmethionine decarboxylase 1	1,20012
	Hs,730672	218680_x_at	C15orf63 /// MIR1282 /// SERF2 /// SERF2-C15ORF63	chromosome 15 open reading frame 63 ///	1,20062
	Hs,520619	218984_at	PUS7	pseudouridylate synthase 7 homolog (S,	1,20114
	Hs,656547	222584_at	MSTO1	misato homolog 1 (Drosophila)	1,20184
	Hs,445977	238880_at	GTF3A	general transcription factor IIIA	1,2028
	Hs,174050	209059_s_at	EDF1	endothelial differentiation-related fac	1,20439
	Hs,143250	201645_at	TNC	tenascin C	1,20497
	Hs,731396	201997_s_at	SPEN	spen homolog, transcriptional regulator	1,20506
	Hs,370262	220097_s_at	TMEM104	transmembrane protein 104	1,20527
	Hs,643566	201460_at	MAPKAPK2	mitogen-activated protein kinase-activa	1,20691
	Hs,512465	222979_s_at	SURF4	surfeit 4	1,20738
	Hs,182626	202027_at	TMEM184B	transmembrane protein 184B	1,20754
	Hs,458917	218143_s_at	SCAMP2	secretory carrier membrane protein 2	1,20795
	Hs,13543	226566_at	TRIM11	tripartite motif containing 11	1,20842
	Hs,9196	47530_at	C9orf156	chromosome 9 open reading frame 156	1,20918
	Hs,42957	204027_s_at	METTL1	methyltransferase like 1	1,20923
	Hs,655969	203643_at	ERF	Ets2 repressor factor	1,21126
	Hs,380857	224578_at	RCC2	regulator of chromosome condensation 2	1,21127
	Hs,107845	219774_at	CCDC93	coiled-coil domain containing 93	1,21138
	Hs,708195	204785_x_at	IFNAR2	interferon (alpha, beta and omega) rece	1,21473
	Hs,465607	215148_s_at	APBA3	amyloid beta (A4) precursor protein-bin	1,21663
	Hs,248785	223184_s_at	AGPAT3	1-acylglycerol-3-phosphate O-acyltransf	1,2172
	Hs,413045	213165_at	CEP350	centrosomal protein 350kDa	1,21834
	Hs,731917	223249_at	CLDN12	claudin 12	1,21851
	Hs,655014	224500_s_at	MON1A	MON1 homolog A (yeast)	1,21906
	Hs,29344	213191_at	TICAM1	toll-like receptor adaptor molecule 1	1,21961
	Hs,571797	230241_at	TOR1AIP2	torsin A interacting protein 2	1,21999
	Hs,4900	208968_s_at	CIAPIN1	cytokine induced apoptosis inhibitor 1	1,22178
	Hs,464333	217796_s_at	NPLOC4	nuclear protein localization 4 homolog	1,22271
	Hs,12341	201786_s_at	ADAR	adenosine deaminase, RNA-specific	1,22388
	Hs,696283	214011_s_at	NOP16	NOP16 nucleolar protein homolog (yeast)	1,22426
	Hs,512465	222977_at	SURF4	surfeit 4	1,22554
	Hs,5019	212313_at	CHMP7	charged multivesicular body protein 7	1,22816
	Hs,458487	208805_at	KIAA0391 /// PSMA6	KIAA0391 /// proteasome (prosome, macro	1,22947
	Hs,46679	227667_at	CUEDC1	CUE domain containing 1	1,2298
	Hs,647333	209836_x_at	BOLA2 /// BOLA2B	bolA homolog 2 (E, coli) /// bolA homol	1,23118
	Hs,500375	201704_at	ENTPD6	ectonucleoside triphosphate diphosphohy	1,23194

	Hs,541177	230528_s_at	LOC100287195 /// LOC100289034 /// LOC100653047 /// LOC1	60S ribosomal protein L23a-like /// rib	1,23263
	Hs,731396	1556059_s_at	SPEN	spen homolog, transcriptional regulator	1,23279
	Hs,129055	210415_s_at	ODF2	outer dense fiber of sperm tails 2	1,23481
	Hs,655396	208776_at	PSMD11	proteasome (prosome, macropain) 26S sub	1,23637
	Hs,13034	230739_at	FAM210A	family with sequence similarity 210, me	1,23882
	Hs,631742	222653_at	PNPO	pyridoxamine 5'-phosphate oxidase	1,23979
	Hs,371001	208688_x_at	EIF3B	eukaryotic translation initiation facto	1,24011
	Hs,730659	202128_at	KIAA0317	KIAA0317	1,24149
	Hs,591908	226694_at	AKAP2 /// PALM2-AKAP2	A kinase (PRKA) anchor protein 2 /// PA	1,24374
	Hs,67896	210443_x_at	OGFR	opioid growth factor receptor	1,24447
	Hs,515154	223419_at	FBXW9	F-box and WD repeat domain containing 9	1,24557
	Hs,648448	214434_at	HSPA12A	heat shock 70kDa protein 12A	1,24953
	Hs,368077	206034_at	SERPINB8	serpin peptidase inhibitor, clade B (ov	1,24991
	Hs,248785	223182_s_at	AGPAT3	1-acylglycerol-3-phosphate O-acyltransf	1,25049
	Hs,390736	214486_x_at	CFLAR	CASP8 and FADD-like apoptosis regulator	1,25237
	Hs,520094	224708_at	KIAA2013	KIAA2013	1,2533
	Hs,67896	202841_x_at	OGFR	opioid growth factor receptor	1,25488
	Hs,513926	215113_s_at	SENP3 /// SENP3-EIF4A1	SUMO1/sentrin/SMT3 specific peptidase 3	1,25606
	Hs,460468	211982_x_at	XPO6	exportin 6	1,25727
	Hs,256301	224468_s_at	C19orf48	chromosome 19 open reading frame 48	1,25903
	Hs,517543	202212_at	PES1	pescadillo ribosomal biogenesis factor	1,25928
	Hs,654958	209246_at	ABCF2	ATP-binding cassette, sub-family F (GCN	1,25988
	Hs,14846	212292_at	SLC7A1	solute carrier family 7 (cationic amino	1,2606
	Hs,336810	221012_s_at	TRIM8	tripartite motif containing 8	1,26419
	Hs,54609	36475_at	GCAT	glycine C-acetyltransferase	1,26535
	Hs,128096	227477_at	ZMYND19	zinc finger, MYND-type containing 19	1,26751
	Hs,591931	204977_at	DDX10	DEAD (Asp-Glu-Ala-Asp) box polypeptide	1,27129
	Hs,603629	226748_at	LYSMD2	LysM, putative peptidoglycan-binding, d	1,27246
	Hs,150423	203198_at	CDK9	cyclin-dependent kinase 9	1,27251
	Hs,468972	212152_x_at	ARID1A	AT rich interactive domain 1A (SWI-like	1,27292
	Hs,735595	211576_s_at	SLC19A1	solute carrier family 19 (folate transp	1,27436
	Hs,409876	1554037_a_at	ZBTB24	zinc finger and BTB domain containing 2	1,275
	Hs,2399	160020_at	MMP14	matrix metallopeptidase 14 (membrane-in	1,27568
	Hs,614194	202519_at	MLXIP	MLX interacting protein	1,27856
	Hs,184877	209003_at	SLC25A11	solute carrier family 25 (mitochondrial	1,28107
	Hs,455109	222191_s_at	B4GALT7	xylosylprotein beta 1,4-galactosyltrans	1,28152
	Hs,516788	212818_s_at	ASB1	ankyrin repeat and SOCS box containing	1,28282
	Hs,632365	227617_at	TMEM201	transmembrane protein 201	1,28549
	Hs,506759	209186_at	ATP2A2	ATPase, Ca++ transporting, cardiac musc	1,28824
	Hs,632041	58696_at	EXOSC4	exosome component 4	1,28935
	Hs,284491	222492_at	PDXK	pyridoxal (pyridoxine, vitamin B6) kina	1,29024
	Hs,731416	223991_s_at	GALNT2	UDP-N-acetyl-alpha-D-galactosamine:poly	1,29054
	Hs,183817	226035_at	USP31	ubiquitin specific peptidase 31	1,29059
	Hs,157394	205012_s_at	HAGH	hydroxyacylglutathione hydrolase	1,29303
	Hs,460645	219540_at	ZNF267	zinc finger protein 267	1,2944
	Hs,413045	204373_s_at	CEP350	centrosomal protein 350kDa	1,29494
	Hs,390736	208485_x_at	CFLAR	CASP8 and FADD-like apoptosis regulator	1,29523
	Hs,54609	205164_at	GCAT	glycine C-acetyltransferase	1,29657
	Hs,731388	203089_s_at	HTRA2	HtrA serine peptidase 2	1,30133

Hs,720829	218296_x_at	MSTO1 /// MSTO2P	misato homolog 1 (Drosophila) /// misat	1,30234
Hs,740396	202896_s_at	SIRPA	signal-regulatory protein alpha	1,3025
Hs,471405	203702_s_at	TTLL4	tubulin tyrosine ligase-like family, me	1,30365
Hs,701718	233803_s_at	MYBBP1A	MYB binding protein (P160) 1a	1,30379
Hs,10094	209402_s_at	SLC12A4	solute carrier family 12 (potassium/chl	1,3041
Hs,321045	214398_s_at	IKBKE	inhibitor of kappa light polypeptide ge	1,30465
Hs,524183	200895_s_at	FKBP4	FK506 binding protein 4, 59kDa	1,30561
Hs,145442	202670_at	MAP2K1	mitogen-activated protein kinase kinase	1,30593
Hs,523710	212860_at	ZDHHC18	zinc finger, DHHC-type containing 18	1,30707
Hs,22678	218590_at	C10orf2	chromosome 10 open reading frame 2	1,31098
Hs,514284	200758_s_at	NFE2L1	nuclear factor (erythroid-derived 2)-li	1,31315
Hs,311190	225201_s_at	MRPL14	mitochondrial ribosomal protein L14	1,31744
Hs,632453	206972_s_at	GPR161	G protein-coupled receptor 161	1,32221
Hs,370671	213300_at	ATG2A	autophagy related 2A	1,32284
Hs,517981	203273_s_at	TUSC2	tumor suppressor candidate 2	1,32414
Hs,284491	218018_at	PDXK	pyridoxal (pyridoxine, vitamin B6) kina	1,3268
Hs,164419	224612_s_at	DNAJC5	DnaJ (Hsp40) homolog, subfamily C, memb	1,3304
Hs,244723	213523_at	CCNE1	cyclin E1	1,33241
Hs,740679	226077_at	RNF145	ring finger protein 145	1,33359
Hs,424312	211564_s_at	PDLIM4	PDZ and LIM domain 4	1,33873
Hs,523715	1560060_s_at	VPS37C	vacuolar protein sorting 37 homolog C (	1,33899
Hs,52788	35265_at	FXR2	fragile X mental retardation, autosomal	1,33929
Hs,731388	211152_s_at	HTRA2	HtrA serine peptidase 2	1,3394
Hs,26010	201037_at	PFKP	phosphofructokinase, platelet	1,33958
Hs,706868	217635_s_at	POLG	polymerase (DNA directed), gamma	1,34018
Hs,193163	202931_x_at	BIN1	bridging integrator 1	1,34113
Hs,361323	202394_s_at	ABCF3	ATP-binding cassette, sub-family F (GCN	1,34128
Hs,355753	224776_at	AGPAT6	1-acylglycerol-3-phosphate O-acyltransf	1,34681
Hs,144073	227146_at	QSOX2	quiescin Q6 sulfhydryl oxidase 2	1,3497
Hs,521151	218068_s_at	ZNF672	zinc finger protein 672	1,34974
Hs,371001	203462_x_at	EIF3B	eukaryotic translation initiation facto	1,35266
Hs,522933	229348_at	UBIAD1	UbiA prenyltransferase domain containin	1,35415
Hs,731801	209100_at	IFRD2	interferon-related developmental regula	1,35464
Hs,64691	1555970_at	FBXO28	F-box protein 28	1,35544
Hs,250493	219314_s_at	ZNF219	zinc finger protein 219	1,35861
Hs,171426	225344_at	NCOA7	nuclear receptor coactivator 7	1,3632
Hs,731427	201471_s_at	SQSTM1	sequestosome 1	1,36484
Hs,579079	213009_s_at	TRIM37	tripartite motif containing 37	1,36544
Hs,284284	225733_at	B3GALT6	UDP-Gal:betaGal beta 1,3-galactosyltran	1,36693
Hs,512799	226871_s_at	ATG4D	autophagy related 4D, cysteine peptidas	1,36786
Hs,517948	204355_at	DHX30	DEAH (Asp-Glu-Ala-His) box polypeptide	1,37421
Hs,368921	204345_at	COL16A1	collagen, type XVI, alpha 1	1,37629
Hs,433203	221597_s_at	TMEM208	transmembrane protein 208	1,37653
Hs,655285	200045_at	ABCF1	ATP-binding cassette, sub-family F (GCN	1,3766
Hs,377484	202387_at	BAG1	BCL2-associated athanogene	1,37712
Hs,740534	219675_s_at	UXS1	UDP-glucuronate decarboxylase 1	1,38105
Hs,643480	225898_at	WDR54	WD repeat domain 54	1,38124
Hs,515610	209229_s_at	PPP6R1	protein phosphatase 6, regulatory subun	1,38192
Hs,591451	225323_at	CC2D1B	coiled-coil and C2 domain containing 1B	1,38213
Hs,91586	238948_at	TM9SF1	Transmembrane 9 superfamily member 1	1,38533
Hs,513797	201195_s_at	SLC7A5	solute carrier family 7 (amino acid tra	1,38572
Hs,107382	223364_s_at	DHX37	DEAH (Asp-Glu-Ala-His) box polypeptide	1,38688

	Hs,713636	223172_s_at	MTFP1	mitochondrial fission process 1	1,38927
	Hs,731702	233430_at	TBC1D22B	TBC1 domain family, member 22B	1,39226
	Hs,522255	234192_s_at	GKAP1	G kinase anchoring protein 1	1,39344
	Hs,377001	202771_at	PIEZO1	piezo-type mechanosensitive ion channel	1,39371
	Hs,147381	242866_x_at	POU2F2	POU class 2 homeobox 2	1,39373
	Hs,522933	219131_at	UBIAD1	UbiA prenyltransferase domain containin	1,39417
	Hs,532492	202767_at	ACP2	acid phosphatase 2, lysosomal	1,39481
	Hs,23111	202159_at	FARSA	phenylalanyl-tRNA synthetase, alpha sub	1,39953
	Hs,160550	226629_at	SLC43A2	solute carrier family 43, member 2	1,40268
	Hs,521092	231876_at	TRIM56	tripartite motif containing 56	1,40484
	Hs,130759	202446_s_at	PLSCR1	phospholipid scramblase 1	1,40524
	Hs,130759	202430_s_at	PLSCR1	phospholipid scramblase 1	1,40822
	Hs,436446	202655_at	MANF	mesencephalic astrocyte-derived neurotr	1,40939
	Hs,6638	232676_x_at	MYEF2	myelin expression factor 2	1,41038
	Hs,530712	239188_at	PPP2R3C	protein phosphatase 2, regulatory subun	1,41395
	Hs,284491	218019_s_at	PDXK	pyridoxal (pyridoxine, vitamin B6) kina	1,41405
	Hs,632367	227970_at	GPR157	G protein-coupled receptor 157	1,41637
	Hs,655455	203149_at	PVRL2	poliovirus receptor-related 2 (herpesvi	1,42014
	Hs,505545	203125_x_at	SLC11A2	solute carrier family 11 (proton-couple	1,42017
	Hs,567488	219920_s_at	AMIGO3 /// GMPPB	adhesion molecule with Ig-like domain 3	1,4251
	Hs,5476	225212_at	SLC25A25	solute carrier family 25 (mitochondrial	1,4304
	Hs,318547	227396_at	LOC100287223 /// PTPRJ	uncharacterized LOC100287223 /// protei	1,43341
	Hs,580681	204502_at	SAMHD1	SAM domain and HD domain 1	1,43986
	Hs,193268	231984_at	MTAP	methylthioadenosine phosphorylase	1,44492
	Hs,522378	225020_at	DAB2IP	DAB2 interacting protein	1,44671
	Hs,558764	201762_s_at	PSME2	proteasome (prosome, macropain) activat	1,44861
	Hs,377992	203573_s_at	RABGGTA	Rab geranylgeranyltransferase, alpha su	1,45335
	Hs,304249	224967_at	UGCG	UDP-glucose ceramide glucosyltransferas	1,45389
	Hs,408730	219400_at	CNTNAP1	contactin associated protein 1	1,45551
	Hs,386567	242907_at	GBP2	guanylate binding protein 2, interferon	1,45571
	Hs,514284	200759_x_at	NFE2L1	nuclear factor (erythroid-derived 2)-li	1,46503
	Hs,521482	204656_at	SHB	Src homology 2 domain containing adapto	1,47285
	Hs,514012	207667_s_at	MAP2K3	mitogen-activated protein kinase kinase	1,48337
	Hs,520414	202727_s_at	IFNGR1	interferon gamma receptor 1	1,48741
	Hs,587054	225007_at	G3BP1	GTPase activating protein (SH3 domain)	1,49054
	Hs,336916	216038_x_at	DAXX	death-domain associated protein	1,49271
	Hs,596514	200078_s_at	ATP6V0B	ATPase, H+ transporting, lysosomal 21kD	1,49306
	Hs,740534	225583_at	UXS1	UDP-glucuronate decarboxylase 1	1,49376
	Hs,4859	1555827_at	CCNL1	Cyclin L1	1,49741
	Hs,591976	204715_at	PANX1	pannexin 1	1,50209
	Hs,378821	225955_at	METRNL	meteorin, glial cell differentiation re	1,5036
	Hs,523715	219053_s_at	VPS37C	vacuolar protein sorting 37 homolog C (	1,50525
	---	1554640_at	PALM2	paralemmin 2	1,51748
	Hs,406530	223113_at	TMEM138	transmembrane protein 138	1,5195
	Hs,702167	201482_at	QSOX1	quiescin Q6 sulfhydryl oxidase 1	1,52193
	Hs,390736	210564_x_at	CFLAR	CASP8 and FADD-like apoptosis regulator	1,52246
	Hs,534330	212859_x_at	LOC100505584 /// MT1E	metallothionein-2-like /// metallothion	1,52977
	Hs,500761	202856_s_at	SLC16A3	solute carrier family 16, member 3 (mon	1,53712
	Hs,708195	204786_s_at	IFNAR2	interferon (alpha, beta and omega) rece	1,53816
	Hs,408702	1554016_a_at	C16orf57	chromosome 16 open reading frame 57	1,53923
	Hs,524910	214211_at	FTH1	ferritin, heavy polypeptide 1	1,54171
	Hs,466871	210845_s_at	PLAUR	plasminogen activator, urokinase recept	1,54489

	Hs,514284	214179_s_at	NFE2L1	nuclear factor (erythroid-derived 2)-li	1,54569
	Hs,477015	223395_at	ABI3BP	ABI family, member 3 (NESH) binding pro	1,54977
	Hs,488827	222995_s_at	RHBDD2	rhomboid domain containing 2	1,55591
	Hs,390736	211317_s_at	CFLAR	CASP8 and FADD-like apoptosis regulator	1,55623
	Hs,502	204769_s_at	TAP2	transporter 2, ATP-binding cassette, su	1,56214
	Hs,310591	205896_at	SLC22A4	solute carrier family 22 (organic catio	1,56309
	Hs,466871	211924_s_at	PLAUR	plasminogen activator, urokinase recept	1,56613
	Hs,352018	202307_s_at	TAP1	transporter 1, ATP-binding cassette, su	1,56618
	Hs,567229	207071_s_at	ACO1	aconitase 1, soluble	1,5738
	Hs,6638	222771_s_at	MYEF2	myelin expression factor 2	1,57427
	Hs,494457	203045_at	NINJ1	ninjurin 1	1,58456
	Hs,440025	202181_at	KIAA0247	KIAA0247	1,59089
	Hs,471991	205323_s_at	MTF1	metal-regulatory transcription factor 1	1,59259
	Hs,740543	219247_s_at	ZDHHC14	zinc finger, DHHC-type containing 14	1,59839
	Hs,473420	213134_x_at	BTG3	BTG family, member 3	1,60038
	Hs,534330	216336_x_at	LOC100505584 /// MT1E	metallothionein-2-like /// metallothion	1,6036
	Hs,596464	231810_at	BRI3BP	BRI3 binding protein	1,60866
	Hs,473420	205548_s_at	BTG3	BTG family, member 3	1,60984
	Hs,657355	227334_at	USP54	ubiquitin specific peptidase 54	1,62418
	Hs,510528	221571_at	TRAF3	TNF receptor-associated factor 3	1,62841
	Hs,129955	206756_at	CHST7	carbohydrate (N-acetylglucosamine 6-O)	1,63138
	Hs,2128	209457_at	DUSP5	dual specificity phosphatase 5	1,63207
	Hs,655177	225316_at	MFSD2A	major facilitator superfamily domain co	1,63617
	Hs,708195	227125_at	IFNAR2	interferon (alpha, beta and omega) rece	1,63703
	Hs,702167	230523_at	QSOX1	quiescin Q6 sulfhydryl oxidase 1	1,63801
	Hs,522507	223050_s_at	FBXW5	F-box and WD repeat domain containing 5	1,64388
	Hs,160786	207076_s_at	ASS1	argininosuccinate synthase 1	1,66559
	Hs,487046	215078_at	LOC100129518 /// SOD2	uncharacterized LOC100129518 /// supero	1,67511
	Hs,488240	203234_at	UPP1	uridine phosphorylase 1	1,67631
	Hs,499952	227804_at	TLCD1	TLC domain containing 1	1,69623
	Hs,648434	226810_at	OGFRL1	opioid growth factor receptor-like 1	1,70054
	Hs,481720	244350_at	MYO10	myosin X	1,7018
	Hs,561514	215411_s_at	TRAF3IP2	TRAF3 interacting protein 2	1,7073
	Hs,464438	225059_at	AGTRAP	angiotensin II receptor-associated prot	1,7122
	Hs,370937	208829_at	TAPBP	TAP binding protein (tapasin)	1,71801
	Hs,310640	226117_at	TIFA	TRAF-interacting protein with forkhead-	1,72797
	Hs,502	225973_at	TAP2	transporter 2, ATP-binding cassette, su	1,72975
	Hs,513626	213629_x_at	MT1F	metallothionein 1F	1,73127
	Hs,580681	234987_at	SAMHD1	SAM domain and HD domain 1	1,73192
	Hs,162125	229435_at	GLIS3	GLIS family zinc finger 3	1,74913
	Hs,481720	236718_at	MYO10	myosin X	1,76014
	Hs,524183	200894_s_at	FKBP4	FK506 binding protein 4, 59kDa	1,76472
	Hs,436061	202531_at	IRF1	interferon regulatory factor 1	1,77297
	Hs,408702	218060_s_at	C16orf57	chromosome 16 open reading frame 57	1,77379
	Hs,147381	235661_at	POU2F2	POU class 2 homeobox 2	1,77522
	Hs,591054	227143_s_at	BID	BH3 interacting domain death agonist	1,77945
	Hs,1027	204802_at	RRAD	Ras-related associated with diabetes	1,78114
	Hs,444947	202241_at	TRIB1	tribbles homolog 1 (Drosophila)	1,7876
	Hs,195155	234973_at	SLC38A5	solute carrier family 38, member 5	1,79629
	Hs,513626	217165_x_at	MT1F	metallothionein 1F	1,8029
	Hs,446091	229630_s_at	WTAP	Wilms tumor 1 associated protein	1,80433
	Hs,591054	204493_at	BID	BH3 interacting domain death agonist	1,80845

Hs,601143	223290_at	PDXP /// SH3BP1	pyridoxal (pyridoxine, vitamin B6) phos	1,81161
Hs,1027	204803_s_at	RRAD	Ras-related associated with diabetes	1,86979
Hs,336916	201763_s_at	DAXX	death-domain associated protein	1,89146
Hs,511251	217995_at	SQRDL	sulfide quinone reductase-like (yeast)	1,91378
Hs,12646	218543_s_at	PARP12	poly (ADP-ribose) polymerase family, me	1,91472
Hs,517617	36711_at	MAFF	v-maf musculoaponeurotic fibrosarcoma o	1,91472
Hs,269128	223430_at	SIK2	salt-inducible kinase 2	1,9168
Hs,591054	211725_s_at	BID	BH3 interacting domain death agonist	1,91701
Hs,445447	223586_at	ARNTL2	aryl hydrocarbon receptor nuclear trans	1,91774
Hs,561514	202987_at	TRAF3IP2	TRAF3 interacting protein 2	1,91907
Hs,404741	204702_s_at	NFE2L3	nuclear factor (erythroid-derived 2)-li	1,92366
Hs,293660	225868_at	TRIM47	tripartite motif containing 47	1,9328
Hs,730686	211139_s_at	NAB1	NGFI-A binding protein 1 (EGR1 binding	1,93602
Hs,630884	218980_at	FHOD3	formin homology 2 domain containing 3	1,93629
Hs,321045	204549_at	IKBKE	inhibitor of kappa light polypeptide ge	1,93802
Hs,517617	205193_at	MAFF	v-maf musculoaponeurotic fibrosarcoma o	1,93993
Hs,631562	209785_s_at	PLA2G4C	phospholipase A2, group IVC (cytosolic,	1,94765
Hs,55999	209706_at	NKX3-1	NK3 homeobox 1	1,94841
Hs,711617	220739_s_at	CNNM3	cyclin M3	1,96179
Hs,237856	219593_at	SLC15A3	solute carrier family 15, member 3	1,96533
Hs,445447	224204_x_at	ARNTL2	aryl hydrocarbon receptor nuclear trans	1,96852
Hs,368912	203716_s_at	DPP4	dipeptidyl-peptidase 4	1,99573
Hs,446091	210285_x_at	WTAP	Wilms tumor 1 associated protein	1,99748
Hs,500761	202855_s_at	SLC16A3	solute carrier family 16, member 3 (mon	2,00899
Hs,355141	207196_s_at	TNIP1	TNFAIP3 interacting protein 1	2,01154
Hs,3781	209841_s_at	LRRN3	leucine rich repeat neuronal 3	2,04729
Hs,99962	213113_s_at	SLC43A3	solute carrier family 43, member 3	2,06136
Hs,440829	213006_at	CEBPD	CCAAT/enhancer binding protein (C/EBP),	2,07226
Hs,505545	203123_s_at	SLC11A2	solute carrier family 11 (proton-couple	2,0784
Hs,381058	228325_at	KIAA0146	KIAA0146	2,09041
Hs,180903	217497_at	TYMP	thymidine phosphorylase	2,11308
Hs,518451	203879_at	PIK3CD	phosphoinositide-3-kinase, catalytic, d	2,11498
Hs,446091	203137_at	WTAP	Wilms tumor 1 associated protein	2,12896
Hs,256278	203508_at	TNFRSF1B	tumor necrosis factor receptor superfam	2,13245
Hs,505545	203124_s_at	SLC11A2	solute carrier family 11 (proton-couple	2,13315
Hs,730686	209272_at	NAB1	NGFI-A binding protein 1 (EGR1 binding	2,13385
Hs,632258	209417_s_at	IFI35	interferon-induced protein 35	2,13828
Hs,458276	203927_at	NFKBIE	nuclear factor of kappa light polypepti	2,16375
Hs,248114	230090_at	GDNF	glial cell derived neurotrophic factor	2,2166
Hs,731813	229450_at	IFIT3	interferon-induced protein with tetratr	2,2449
Hs,181301	202901_x_at	CTSS	cathepsin S	2,26978
Hs,654542	205870_at	BDKRB2	bradykinin receptor B2	2,2792
Hs,129798	226064_s_at	DGAT2	diacylglycerol O-acyltransferase 2	2,30942
Hs,489051	225871_at	STEAP2	STEAP family member 2, metalloreductase	2,31757
Hs,190622	218943_s_at	DDX58	DEAD (Asp-Glu-Ala-Asp) box polypeptide	2,34402
Hs,3781	209840_s_at	LRRN3	leucine rich repeat neuronal 3	2,35756
Hs,647370	217546_at	MT1M	metallothionein 1M	2,35896
Hs,445447	220658_s_at	ARNTL2	aryl hydrocarbon receptor nuclear trans	2,39169
Hs,201398	220975_s_at	C1QTNF1	C1q and tumor necrosis factor related p	2,51528
Hs,319171	223218_s_at	NFKBIZ	nuclear factor of kappa light polypepti	2,53523
Hs,376289	231899_at	ZC3H12C	zinc finger CCCH-type containing 12C	2,53541
Hs,61635	205542_at	STEAP1	six transmembrane epithelial antigen of	2,53839
Hs,76095	201631_s_at	IER3	immediate early response 3	2,54668

	Hs,484047	236725_at	WWC1	WW and C2 domain containing 1	2,59684
	Hs,244378	220091_at	SLC2A6	solute carrier family 2 (facilitated gl	2,64016
	Hs,521212	201272_at	AKR1B1	aldo-keto reductase family 1, member B1	2,65977
	Hs,731813	204747_at	IFIT3	interferon-induced protein with tetratr	2,66992
	Hs,730030	229872_s_at	LOC100132999 /// LOC642441 /// LOC730256	uncharacterized LOC100132999 /// unchar	2,68411
	Hs,129944	208394_x_at	ESM1	endothelial cell-specific molecule 1	2,69884
	Hs,458485	205483_s_at	ISG15	ISG15 ubiquitin-like modifier	2,77387
	Hs,132441	213256_at	MARCH3	membrane-associated ring finger (C3HC4)	2,78153
	Hs,518055	211596_s_at	LRIG1	leucine-rich repeats and immunoglobulin	2,79975
	Hs,163173	219209_at	IFIH1	interferon induced with helicase C doma	2,80944
	Hs,532634	202411_at	IFI27	interferon, alpha-inducible protein 27	2,83624
	Hs,131180	220987_s_at	AKIP1 /// NUAK2	A kinase (PRKA) interacting protein 1 /	2,83785
	Hs,449207	231779_at	IRAK2	interleukin-1 receptor-associated kinas	2,86602
	Hs,489615	217739_s_at	NAMPT	nicotinamide phosphoribosyltransferase	2,87713
	Hs,369265	213817_at	IRAK3	interleukin-1 receptor-associated kinas	2,93579
	Hs,527778	203904_x_at	CD82	CD82 molecule	2,94451
	Hs,147381	227749_at	POU2F2	POU class 2 homeobox 2	2,96601
	Hs,660998	241986_at	BMPER	BMP binding endothelial regulator	2,97012
	Hs,442619	209928_s_at	MSC	musculin	2,97198
	Hs,491232	212110_at	SLC39A14	solute carrier family 39 (zinc transpor	2,98056
	Hs,525634	218627_at	DRAM1	DNA-damage regulated autophagy modulato	3,06634
	Hs,489615	217738_at	NAMPT	nicotinamide phosphoribosyltransferase	3,1677
	Hs,181301	232617_at	CTSS	cathepsin S	3,17809
	Hs,180903	204858_s_at	TYMP	thymidine phosphorylase	3,197
	Hs,484047	213085_s_at	WWC1	WW and C2 domain containing 1	3,20234
	Hs,89714	215101_s_at	CXCL5	chemokine (C-X-C motif) ligand 5	3,30204
	Hs,319171	223217_s_at	NFKBIZ	nuclear factor of kappa light polypepti	3,31037
	Hs,448520	225516_at	SLC7A2	solute carrier family 7 (cationic amino	3,33496
	Hs,525607	202510_s_at	TNFAIP2	tumor necrosis factor, alpha-induced pr	3,41043
	Hs,181301	202902_s_at	CTSS	cathepsin S	3,4444
	Hs,467304	206924_at	IL11	interleukin 11	3,70843
	Hs,489615	1555167_s_at	NAMPT	nicotinamide phosphoribosyltransferase	3,78612
	Hs,81328	201502_s_at	NFKBIA	nuclear factor of kappa light polypepti	3,94985
	Hs,118552	218834_s_at	TMEM132A	transmembrane protein 132A	3,9741
	Hs,196384	204748_at	PTGS2	prostaglandin-endoperoxide synthase 2 (	4,01328
	Hs,73853	205289_at	BMP2	bone morphogenetic protein 2	4,36982
	Hs,528634	218400_at	OAS3	2'-5'-oligoadenylate synthetase 3, 100k	4,39633
	Hs,196384	1554997_a_at	PTGS2	prostaglandin-endoperoxide synthase 2 (	4,65879
	Hs,654402	205205_at	RELB	v-rel reticuloendotheliosis viral oncog	5,12017
	Hs,529317	219352_at	HERC6	HECT and RLD domain containing E3 ubiqu	5,29281
	Hs,25590	204597_x_at	STC1	stanniocalcin 1	5,33836
	Hs,656294	218810_at	ZC3H12A	zinc finger CCCH-type containing 12A	5,47868
	Hs,25590	204595_s_at	STC1	stanniocalcin 1	5,48652
	Hs,211600	202644_s_at	TNFAIP3	tumor necrosis factor, alpha-induced pr	5,5126
	Hs,86724	204224_s_at	GCH1	GTP cyclohydrolase 1	6,02488
	Hs,127799	210538_s_at	BIRC3	baculoviral IAP repeat containing 3	6,86989
	Hs,735831	232504_at	LOC285628 /// MIR146A	uncharacterized LOC285628 /// microRNA	7,48035
	Hs,303649	216598_s_at	CCL2	chemokine (C-C motif) ligand 2	7,49042
	Hs,211600	202643_s_at	TNFAIP3	tumor necrosis factor, alpha-induced pr	8,11744
	Hs,487046	216841_s_at	LOC100129518 /// SOD2	uncharacterized LOC100129518 /// supero	9,07478
	Hs,517307	202086_at	MX1	myxovirus (influenza virus) resistance	9,3158

	Hs,487046	221477_s_at	LOC100129518 /// SOD2	uncharacterized LOC100129518 /// supero	9,6412
	Hs,439060	222549_at	CLDN1	claudin 1	10,8917
	Hs,654458	205207_at	IL6	interleukin 6 (interferon, beta 2)	11,0765
	Hs,288034	209267_s_at	SLC39A8	solute carrier family 39 (zinc transpor	12,314
	Hs,69771	202357_s_at	CFB	complement factor B	13,9312
	Hs,643447	202637_s_at	ICAM1	intercellular adhesion molecule 1	14,2218
	Hs,487046	215223_s_at	LOC100129518 /// SOD2	uncharacterized LOC100129518 /// supero	15,2587
	Hs,529053	217767_at	C3	complement component 3	18,4476
	Hs,1695	204580_at	MMP12	matrix metallopeptidase 12 (macrophage	20,1285
	Hs,164021	206336_at	CXCL6	chemokine (C-X-C motif) ligand 6 (granu	20,3977
	Hs,643447	202638_s_at	ICAM1	intercellular adhesion molecule 1	22,6303
	Hs,75765	209774_x_at	CXCL2	chemokine (C-X-C motif) ligand 2	33,0477
	Hs,89714	214974_x_at	CXCL5	chemokine (C-X-C motif) ligand 5	36,827
	Hs,112242	223484_at	C15orf48	chromosome 15 open reading frame 48	39,9899
	Hs,789	204470_at	CXCL1	chemokine (C-X-C motif) ligand 1 (melan	45,7707
	Hs,89690	207850_at	CXCL3	chemokine (C-X-C motif) ligand 3	58,8017
	Hs,624	202859_x_at	IL8	interleukin 8	61,9013
	Hs,624	211506_s_at	IL8	interleukin 8	153,58

Supplemental Table 4. Common Genes expressed in primed stromal cells. Green line indicates genes overexpressed in unprimed stromal cells. Red line indicates genes overexpressed in primed stromal cells. Fold Change corresponds to the ratio of median expression in PMN-primed / unprimed stroma.

	Gene Symbol	Gene Title	MSCs fold change	Resto cells fold change
	**DCLK1**	doublecortin-like kinase 1	-3,54742	-1,54651
	**EBF1**	Early B-cell factor 1	-3,054	-1,42731
	**CCDC80**	coiled-coil domain containing 80	-2,77843	-1,61597
	**EHBP1**	EH domain binding protein 1	-2,53303	-1,37868
	**PPAP2B**	phosphatidic acid phosphatase type 2B	-2,49452	-1,56807
	**PXK**	PX domain containing serine/threonine kinase	-2,14798	-1,47754
	**LOC100506870**	uncharacterized LOC100506870	-2,03306	-1,82245
	**CDH6**	cadherin 6, type 2, K-cadherin (fetal kidney)	-1,91918	-1,38974
	**RCAN2**	regulator of calcineurin 2	-1,86533	-2,11033
	**DBT**	dihydrolipoamide branched chain transacylase E2	-1,84993	-1,24948
	**LOC100130417**	Uncharacterized LOC100130417	-1,84707	-2,06382
	**SEMA6D**	sema domain, transmembrane domain (TM), and cytoplasmic domain, (semaphorin) 6D	-1,74576	-1,67935
	**PIK3R1**	phosphoinositide-3-kinase, regulatory subunit 1 (alpha)	-1,72176	-1,22391
	**ALDH6A1**	aldehyde dehydrogenase 6 family, member A1	-1,71783	-1,46763
	**FHL1**	four and a half LIM domains 1	-1,68084	-1,31031
	**SSBP2**	single-stranded DNA binding protein 2	-1,61296	-1,62303
	**JAZF1**	JAZF zinc finger 1	-1,60595	-1,37949
	**LOC642852**	uncharacterized LOC642852	-1,5765	-1,27177
	**PLA2G12A**	phospholipase A2, group XIIA	-1,56299	-1,25584
	**RHOBTB3**	Rho-related BTB domain containing 3	-1,55988	-1,55339
	**OXR1**	oxidation resistance 1	-1,55976	-1,37096
	**LOC401093**	uncharacterized LOC401093	-1,54678	-1,28363
	**PRMT2**	protein arginine methyltransferase 2	-1,54097	-1,37467
	**SLC2A12**	solute carrier family 2 (facilitated glucose transporter), member 12	-1,53139	-2,17444
	**PRRX1**	paired related homeobox 1	-1,51919	-1,23866
	**SRGAP2 /// SRGAP2C**	SLIT-ROBO Rho GTPase activating protein 2 /// SLIT-ROBO Rho GTPase activating protein 2	-1,51304	-1,22417
	**ZMAT3**	zinc finger, matrin-type 3	-1,49879	-1,50826
	**ADD3**	adducin 3 (gamma)	-1,45827	-1,40133
	**DPH5**	DPH5 homolog (S, cerevisiae)	-1,44722	-1,21397
	**CCNYL1**	cyclin Y-like 1	-1,43583	-1,28994
	**HAPLN1**	hyaluronan and proteoglycan link protein 1	-1,43052	-1,71462
	**KIAA0355**	KIAA0355	-1,41934	-1,43404
	**PRICKLE1**	prickle homolog 1 (Drosophila)	-1,40629	-1,38127
	**FBXO3**	F-box protein 3	-1,28637	-1,25784
	**DENND1B**	DENN/MADD domain containing 1B	-1,37761	-1,6031
	**ULK2**	unc-51-like kinase 2 (C, elegans)	-1,36556	-1,22016
	**OGFOD1**	2-oxoglutarate and iron-dependent oxygenase domain containing 1	-1,36299	-1,49869
	**NNT**	nicotinamide nucleotide transhydrogenase	-1,35705	-1,21883
	**GLUD1**	glutamate dehydrogenase 1	-1,35523	-1,32782
	**BICD1**	bicaudal D homolog 1 (Drosophila)	-1,3481	-1,25483
	**VPS41**	vacuolar protein sorting 41 homolog (S, cerevisiae)	-1,34364	-1,3482
	**FAM155A**	family with sequence similarity 155, member A	-1,33919	-1,40561

	**SLC25A27**	solute carrier family 25, member 27	-1,33916	-2,02556
	**THRA**	thyroid hormone receptor associated protein 3	-1,36446	-1,21289
	**TCF7L2**	transcription factor 7-like 2 (T-cell specific, HMG-box)	-1,32201	-1,27111
	**ANGEL2**	angel homolog 2 (Drosophila)	-1,29848	-1,22691
	**LOC100288911**	uncharacterized LOC100288911	-1,29334	-1,30923
	**HOXB3**	homeobox B3	-1,2851	-1,21864
	**SYNPO2**	synaptopodin 2	-1,28436	-1,98124
	**RNASE4**	ribonuclease, RNase A family, 4	-1,28086	-1,27428
	**CLIP4**	CAP-GLY domain containing linker protein family, member 4	-1,27264	-1,4306
	**FAM76B**	family with sequence similarity 76, member B	-1,25721	-1,25093
	**ORMDL1**	ORM1-like 1 (S, cerevisiae)	-1,25244	-1,48209
	**C22orf32**	chromosome 22 open reading frame 32	-1,24565	-1,24782
	**CYBRD1**	cytochrome b reductase 1	-1,24032	-1,54538
	**DLX1**	distal-less homeobox 1	-1,23223	-1,45634
	**ZADH2**	zinc binding alcohol dehydrogenase domain containing 2	-1,22849	-1,5115
	**SWAP70**	SWAP switching B-cell complex 70kDa subunit	-1,22529	-1,27995
	**RBMS3**	RNA binding motif, single stranded interacting protein 3	-1,22263	-1,42844
	**PTPRM**	protein tyrosine phosphatase, receptor type, M	-1,21721	-1,32661
	**PDGFRA**	platelet-derived growth factor receptor, alpha polypeptide	-1,20563	-1,23141
	**FNBP1**	formin binding protein 1	-1,20456	-1,26249
	**TNC**	tenascin C	1,20089	1,20906
	**USP31**	ubiquitin specific peptidase 31	1,20486	1,40114
	**EWSR1**	Ewing sarcoma breakpoint region 1	1,21709	1,28079
	**PDIA4**	protein disulfide isomerase family A, member 4	1,21789	1,25001
	**SERPINE1**	serpin peptidase inhibitor, clade E (nexin, plasminogen activator inhibitor type 1), me	1,21986	1,32268
	**CSNK1E**	casein kinase 1, epsilon	1,23112	1,37035
	**RRM2**	ribonucleotide reductase M2	1,23537	1,66426
	**NOC4L**	nucleolar complex associated 4 homolog (S, cerevisiae)	1,23984	1,33348
	**ZDHHC18**	zinc finger, DHHC-type containing 18	1,24193	1,37564
	**GOPC**	golgi-associated PDZ and coiled-coil motif containing	1,24646	1,38795
	**DDX23**	DEAD (Asp-Glu-Ala-Asp) box polypeptide 23	1,25485	1,43051
	**SQSTM1**	sequestosome 1	1,26578	2,05908
	**FBXW9**	F-box and WD repeat domain containing 9	1,27274	1,21899
	**AMIGO3 /// GMPPB**	adhesion molecule with Ig-like domain 3 /// GDP-mannose pyrophosphorylase B	1,27476	1,59316
	**BRD4**	bromodomain containing 4	1,27698	1,4069
	**MTAP**	methylthioadenosine phosphorylase	1,29988	1,60614
	**MRPL14**	mitochondrial ribosomal protein L14	1,32147	1,31347
	**SPATA2**	spermatogenesis associated 2	1,33672	1,20289
	**FRMD4A**	FERM domain containing 4A	1,34285	1,53761
	**PPIF**	peptidylprolyl isomerase F	1,34597	3,24199
	**MANF**	mesencephalic astrocyte-derived neurotrophic factor	1,35023	1,47114
	**LRP8**	low density lipoprotein receptor-related protein 8, apolipoprotein e receptor	1,35629	1,41976
	**MBD3**	methyl-CpG binding domain protein 3	1,37232	1,24691
	**PDLIM4**	PDZ and LIM domain 4	1,38467	1,29431
	**PDXK**	pyridoxal (pyridoxine, vitamin B6) kinase	1,68762	1,41633
	**PPP6R1**	protein phosphatase 6, regulatory subunit 1	1,39599	1,368
	**IFNAR2**	interferon (alpha, beta and omega) receptor 2	1,39667	1,91877
	**GCAT**	glycine C-acetyltransferase	1,40021	1,20061
	**REL**	v-rel reticuloendotheliosis viral oncogene homolog (avian)	1,40689	1,4154

	**IDH3A**	isocitrate dehydrogenase 3 (NAD+) alpha	1,40741	1,31586
	**NFE2L1**	nuclear factor (erythroid-derived 2)-like 1	1,7417	1,33967
	**MCM7**	minichromosome maintenance complex component 7	1,45517	1,63805
	**HYI**	hydroxypyruvate isomerase (putative)	1,46527	1,26459
	**IFRD2**	interferon-related developmental regulator 2	1,47208	1,24657
	**HLA-F**	major histocompatibility complex, class I, F	1,52075	1,23582
	**TMEM158**	Glutaminase	1,52234	2,2642
	**PSME2**	proteasome (prosome, macropain) activator subunit 2 (PA28 beta)	1,53842	1,36403
	**PTGDS**	prostaglandin D2 synthase 21kDa (brain)	1,54077	1,37455
	**NAB1**	NGFI-A binding protein 1 (EGR1 binding protein 1)	1,57531	1,6323
	**MAP2K3**	mitogen-activated protein kinase kinase 3	1,59045	1,3835
	**ZC3HAV1**	zinc finger CCCH-type, antiviral 1	1,60137	1,30975
	**NPAS2**	neuronal PAS domain protein 2	1,69659	1,44263
	**PIM3**	pim-3 oncogene	1,70921	1,34349
	**DTWD1**	DTW domain containing 1	1,82506	1,231
	**MGC24103**	uncharacterized MGC24103	1,98479	1,31997
	**NFKBIZ**	nuclear factor of kappa light polypeptide gene enhancer in B-cells inhibitor, zeta	2,25228	2,85356
	**TNIP1**	TNFAIP3 interacting protein 1	2,28841	1,59095
	**MAFF**	v-maf musculoaponeurotic fibrosarcoma oncogene homolog F (avian)	2,61253	1,4405
	**PION**	pigeon homolog (Drosophila)	2,83149	2,63161
	**CA12**	carbonic anhydrase XII	2,83741	1,8534
	**MSC**	musculin	3,18359	2,77444
	**TGIF1**	TGFB-induced factor homeobox 1	3,33847	1,24103
	**BDKRB2**	bradykinin receptor B2	3,36972	1,5416
	**COL7A1**	collagen, type VII, alpha 1	3,39883	1,65029
	**EPSTI1**	epithelial stromal interaction 1 (breast)	4,0458	1,58959
	**NRP2**	neuropilin 2	4,14217	1,66027
	**TNFAIP2**	tumor necrosis factor, alpha-induced protein 2	4,23039	2,74942
	**NFKBIA**	nuclear factor of kappa light polypeptide gene enhancer in B-cells inhibitor, alpha	4,74509	3,28789
	**TNFAIP6**	nuclear factor of kappa light polypeptide gene enhancer in B-cells inhibitor, alpha	4,74509	3,28789
	**TNFAIP3**	tumor necrosis factor, alpha-induced protein 3	5,47167	5,55381
	**RELB**	v-rel reticuloendotheliosis viral oncogene homolog B	6,37181	4,11439
	**CCL2**	chemokine (C-C motif) ligand 2	7,80961	7,18404
	**STC1**	stanniocalcin 1	9,80787	2,82574
	**LOC100129518 /// SOD2**	stanniocalcin 1	9,80787	2,82574
	**ICAM1**	intercellular adhesion molecule 1	16,68	14,9248
	**CFB**	complement factor B	20,7019	9,37489
	**CXCL2**	chemokine (C-X-C motif) ligand 2	20,9874	52,0387
	**C3**	complement component 3	25,6036	13,2918
	**CXCL1**	chemokine (C-X-C motif) ligand 1 (melanoma growth stimulating activity, alpha)	63,446	33,0198
	**CXCL3**	chemokine (C-X-C motif) ligand 3	69,0519	50,0736
	**IL8**	interleukin 8	93,437	41,0083

Supplemental Figure 1

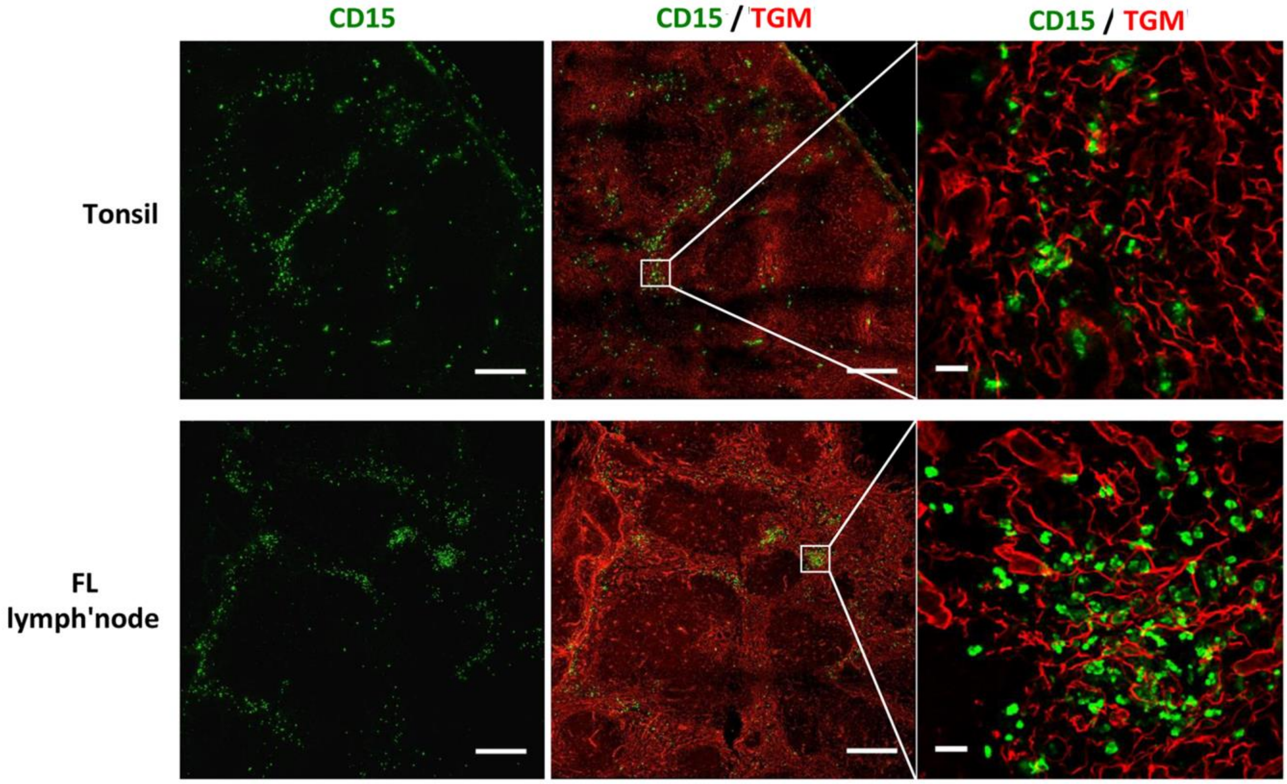

Figure S1. Visualization of infiltrating neutrophils *in situ*.
Tissues were snap frozen in OCT (Tissue-Tek OCT Compound). Sixteen micrometers-thick sections were fixed in 4% PFA, incubated for 1 hour with a blocking solution (PBS, 10% BSA, 10% Donkey serum, 0.1% Saponin) then incubated overnight at 4°C with the following primary antibodies: CD15 (mouse IgM, dilution 1/50, SouthernBiotech) and transglutaminase (mouse IgG1, dilution 1/50, Abcam). After washes, slides were incubated with secondary antibodies: donkey anti-mouse IgM Alexa Fluor 488 and donkey anti-mouse IgG1 Alexa Fluor 594 (Jackson ImmunoResearch). Finally, sections were mounted in Mowiol antifade reagent containing Sytox Blue (Life technologies) for nuclei staining and analyzed by confocal microscopy on a Leica SP5. ImageJ software was used for image analysis.
Neutrophils are stained by anti-CD15 (green) and FRC by anti-transglutaminase (TGM, red). Scale bars, 20µm (left and middle) and 400µm (right).

Supplemental Figure 2

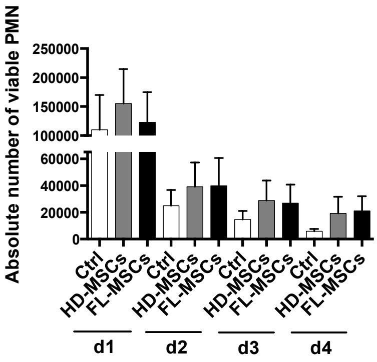

Figure S2. FL-MSCs sustain neutrophil survival.
Purified peripheral blood neutrophils were cultured alone (Ctrl), or in the presence of HD-MSCs (n=4), or FL-MSCs (n=4). The absolute number of CD66b^{pos}CD105^{neg}TOPRO-3^{neg} viable neutrophils was assessed using flow count beads. Results are represented as means ± SD.

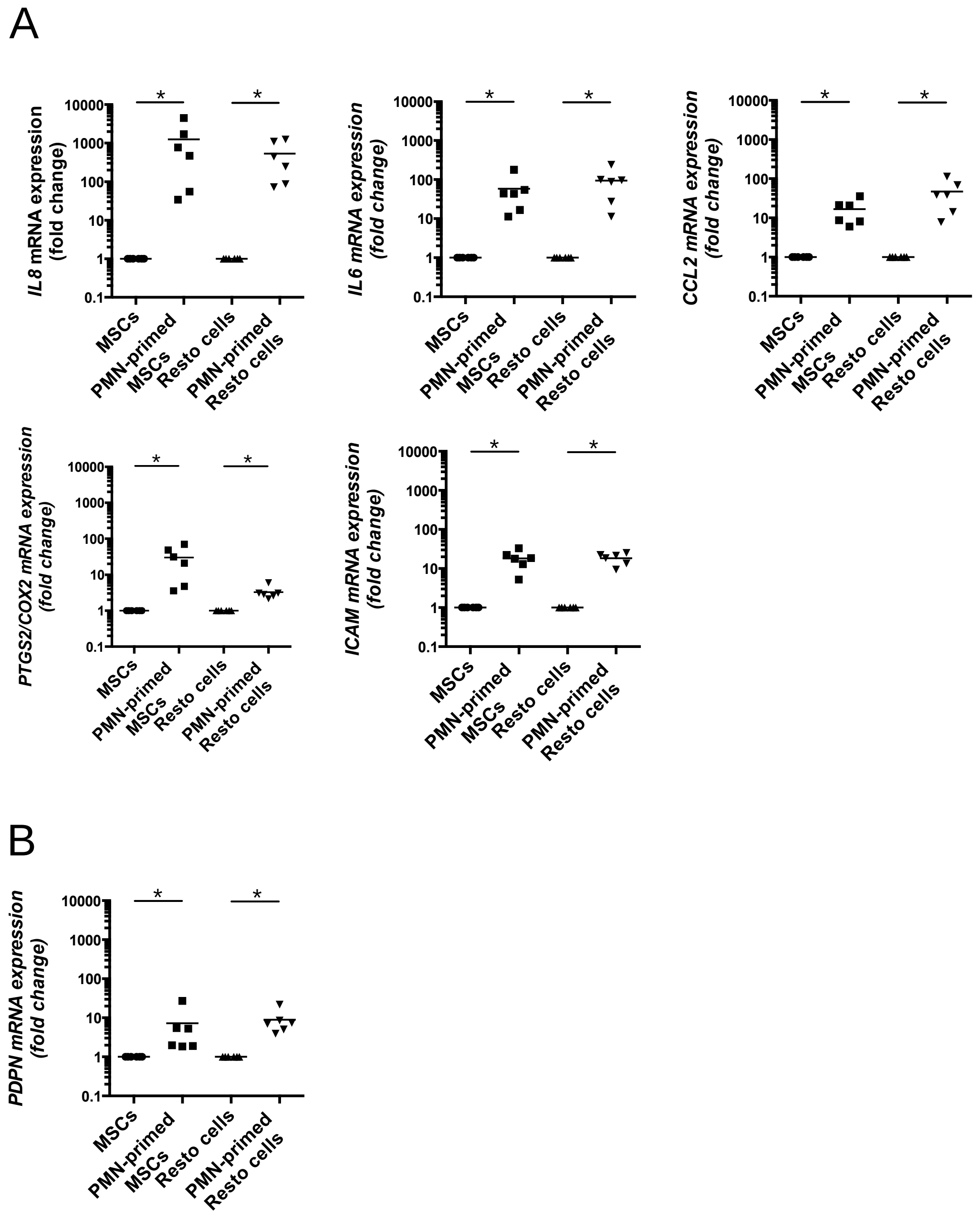

Figure S3. Expression of PMN-induced genes in stromal cells

A. Expression of *IL8*, *IL6*, *ICAM*, *CCL2* and *PTGS2/COX2* was quantified by RQ-PCR in stromal cells primed with neutrophils compared to unprimed stromal cells. Each sample was normalized to *PUM1*, and the arbitrary value of 1 was assigned to the median expression of unprimed stromal cells. Results represent the mean ± SD from 6 experiments. * $P < .05$. **B.** Expression of *PDPN* was quantified by RQ-PCR in stromal cells primed with neutrophils compared to unprimed stromal cells and normalized to *PUM1*, and the arbitrary value of 1 was assigned to the median expression of unprimed stromal cells. Results represent the mean ± SD from 6 experiments. * $P < .05$.
